# Inactivation of *LACCASE8* and *LACCASE5* genes in *Brachypodium distachyon* leads to severe decrease in lignin content and high increase in saccharification yield without impacting plant integrity

**DOI:** 10.1186/s13068-019-1525-5

**Published:** 2019-07-15

**Authors:** Philippe Le Bris, Yin Wang, Clément Barbereau, Sébastien Antelme, Laurent Cézard, Frédéric Legée, Angelina D’Orlando, Marion Dalmais, Abdelhafid Bendahmane, Mathias Schuetz, Lacey Samuels, Catherine Lapierre, Richard Sibout

**Affiliations:** 1 Institut Jean-Pierre Bourgin, INRA, AgroParisTech, CNRS, Université Paris-Saclay, Versailles, France; 2 Institute of Plant Sciences Paris Saclay IPS2, CNRS, INRA, Université Paris-Sud, Université Evry, Université Paris-Saclay, Batiment 630, 91405 Orsay, France; 3 Institute of Plant Sciences Paris-Saclay IPS2, Paris Diderot, Sorbonne Paris-Cité, Bâtiment 630, 91405 Orsay, France; 4 Department of Botany, University of British Columbia, Vancouver, BC V6T 1Z4 Canada; 5 UR1268 BIA (Biopolymères Interactions Assemblages), INRA, 44300 Nantes, France

**Keywords:** Laccase, Oxidation, Lignin, Saccharification, Ferulic acid, Grass crop, Fibers, Xylem, *Brachypodium*

## Abstract

**Background:**

Dedicated lignocellulosic feedstock from grass crops for biofuel production is extensively increasing. However, the access to fermentable cell wall sugars by carbohydrate degrading enzymes is impeded by lignins. These complex polymers are made from reactive oxidized monolignols in the cell wall. Little is known about the laccase-mediated oxidation of monolignols in grasses, and inactivation of the monolignol polymerization mechanism might be a strategy to increase the yield of fermentable sugars.

**Results:**

LACCASE5 and LACCASE8 are inactivated in a *Brachypodium* double mutant. Relative to the wild type, the lignin content of extract-free mature culms is decreased by 20–30% and the saccharification yield is increased by 140%. Release of ferulic acid by mild alkaline hydrolysis is also 2.5-fold higher. Interfascicular fibers are mainly affected while integrity of vascular bundles is not impaired. Interestingly, there is no drastic impact of the double mutation on plant growth.

**Conclusion:**

This work shows that two *Brachypodium* laccases with clearly identified orthologs in crops are involved in lignification of this model plant. Lignification in interfascicular fibers and metaxylem cells is partly uncoupled in *Brachypodium*. Orthologs of these laccases are promising targets for improving grass feedstock for cellulosic biofuel production.

**Electronic supplementary material:**

The online version of this article (10.1186/s13068-019-1525-5) contains supplementary material, which is available to authorized users.

## Background

The optimum utilization of plant biomass as an alternative to fossil carbon in order to produce biomolecules, bioenergy and biomaterials is a major challenge for the next decade [1]. Lignocellulose biomass is mainly composed of hemicellulose and cellulose polysaccharides, and phenolic polymers called lignins. It is well established that lignins negatively affect the cellulose-to-ethanol conversion process [2], but lignins also represent potential sources of high-value products [1]. As grasses, such as *Miscanthus* spp. and switchgrass (*Panicum virgatum*) are considered promising bioenergy crops, there is increasing interest in their lignin composition and deposition. Lignins are polymerized by the oxidative coupling of *p*-coumaryl, coniferyl and sinapyl alcohols (monolignols), which give rise to *p*-hydroxyphenyl (H), guaiacyl (G) and syringyl (S) units within the lignin polymer, respectively. In addition, lignins formed in grasses (Poaceae) have additional features, the main ones being their association to ferulic acid (FA) and *p*-coumaric acid (CA) [3–8].

The polymerization of monolignols is facilitated by peroxidase and laccase proteins [9]. In planta, the role of laccases in lignification was first revealed in Arabidopsis and poplar plants [10–12]. Less is known about laccases in monocotyledonous plants [13, 14], although recent mutant analyses in *Brachypodium distachyon* (*Brachypodium*) have proposed roles in lignification for LACCASE5 (*LAC5*, Bradi1g66720) and potentially LACCASE6 (LAC6, Bradi1g74320) [15]. While the lignin contents of the mature culms from wild type (WT) and *lac6* mutant were similar, the *lac5* mutant had a lower lignin content (~ 10% lower) [15]. Considering the limited reductions in the *lac5* mutant, we continued to search for additional components that facilitate lignin formation in the model *Brachypodium* plant. The present study identifies and characterizes the function of LACCASE8 (*LAC8*, Bradi2g23370), which has an important function in the lignification of cell walls in *Brachypodium distachyon* stems.

## Results and discussion

### *LAC5* and *LAC8* are close paralogs with similar expression pattern

**Fig. 1:**
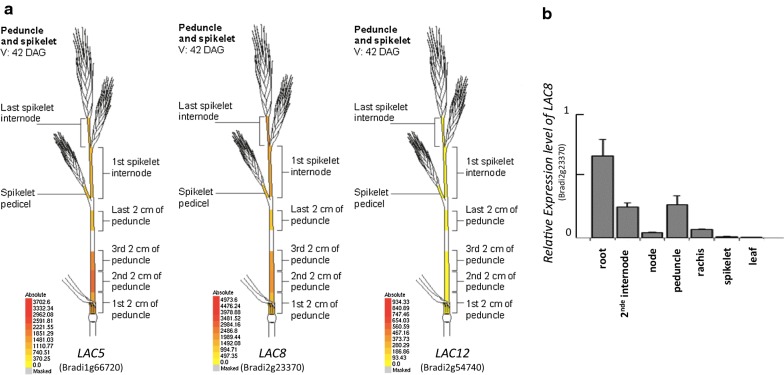
Expression levels of *LAC5*, *LAC8* and *LAC12*. **a** In silico expression levels of the three laccases obtained with the Gene Atlas tool from BAR Brachypodium eFP Browser (http://bar.utoronto.ca/efp_brachypodium/cgi-bin/efpWeb.cgi) [18]. **b** qPCR analysis shows the relative expression levels of *LACCASE8* transcript in different tissues

The most recent revision (v3.1) of the *Brachypodium* accession Bd21 genome [16, 17] induced changes in position of some LACCASE genes in the genome (Additional file 1) compared to earlier published data [15]. To update our data set and identify new genes involved in lignification, we conducted phylogenetic analysis of the entire *Brachypodium* laccase family. We used the Blastp tool from Phytozome (https://phytozome.jgi.doe.gov/) to identify homologs of LAC5 (Bradi1g66720) in *Zea mays*, *Oryza sativa* and *Setaria viridis* (Additional file 2). We then selected proteins with the highest e-score (> 50%) and compared them with laccases published in [15]. We reconstructed a phylogeny tree with the maximum likelihood comparison method (Additional file 3). Despite some names changed for a few laccases (Additional file 1), no major alteration in clusters were found compared with our previous phylogeny analysis [15]. Consistently, LAC5 was most similar to and clustered together with LAC8 and LACCASE12 (LAC12, Bradi2g54740) protein sequences. Considering that the lignin content of the *lac5* mutant was found to be only moderately reduced relative to the WT level [15], we hypothesized that another close paralog might be involved in lignification and that its activity might compensate for LAC5 deficiency. We thus analyzed in silico expression levels of the two closest paralogs *LAC8* and *LAC12* with the Gene Atlas tool from PlaNet (http://aranet.mpimp-golm.mpg.de/ and the BAR Brachypodium eFP Browser (http://bar.utoronto.ca/efp_brachypodium/cgi-bin/efpWeb.cgi) [18]. *LAC8* but not *LAC12* showed a similar expression pattern with *LAC5* (http://bar.utoronto.ca/efp_brachypodium/cgi-bin/efpWeb.cgi, Fig. 1a). In line with this result, signal values relative to *LAC8* and *LAC5* transcript levels were both comparable and much higher than those of *LAC12* in lignified tissues (Fig. 1a). We verified the expression pattern of *LAC8* by qRT-PCR and confirmed data obtained in silico with BAR Brachypodium eFP Browser (Fig. 1b). Indeed, *LAC8* is highly expressed in all lignified tissues of *Brachypodium* (internode, node, peduncle) but poorly detected in developing leaves and young spikelets (Fig. 1b). At last, it is interesting to note that *LAC6*, *LAC8*, *LAC10* (Bradi2g54680) but not *LAC12* belongs the co-expressed network of *LAC5* (Additional file 4). Insofar as *LAC8* is a close paralog of *LAC5* with a highly similar expression pattern, we hypothesized that *LAC8* was most probably involved in cell wall lignification.

### Production and characterization of a *lac5 lac8* double mutant

**Fig. 2:**
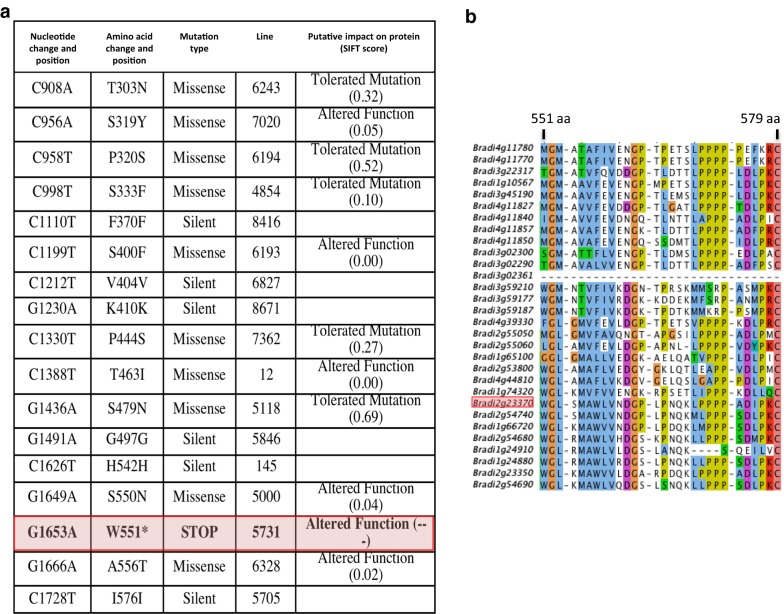
Allelic series of mutations in *LAC8* gene (*Bradi2g23370*) identified by TILLING and peptide alignment of different *Brachypodium* laccase C-terminals. **a** Features of the mutagenized lines identified by TILLING. Lines harboring mutation in non-translated region (intron, 5′ or 3′UTR) were discarded from the table. **b** Alignment of amino acids present in the C-terminal region of all *Brachypodium* laccases present in the genome of *Brachypodium distachyon* Bd21-3 accession. The codon encoding tryptophan at position 551 is replaced by a stop codon in the mutant line *Bd573*1. Colors illustrate the conserved regions between proteins

We used the TILLING (Targeting-Induced Local Lesions in Genomes) facility available for *Brachypodium* at University of Saclay, France (http://www.ips2.u-psud.fr/), to identify *lac8* mutants from a sodium azide mutant collection [19]. Twenty independent lines with mutations in the screened genomic region were isolated using this approach (Fig. 2a). SIFT software analyses revealed which substitutions might partially or totally disrupt the protein activity (Fig. 2a) [19]. By so doing, we selected one homozygous line (line *5731*) which harbored an induced stop codon mutation for further analysis. The premature stop codon in this line is predicted to produce a truncated LAC8 protein that lacks the last 29 amino acids of the highly conserved C-terminal region of the LACCASE protein family (Fig. 2b).

**Fig. 3:**
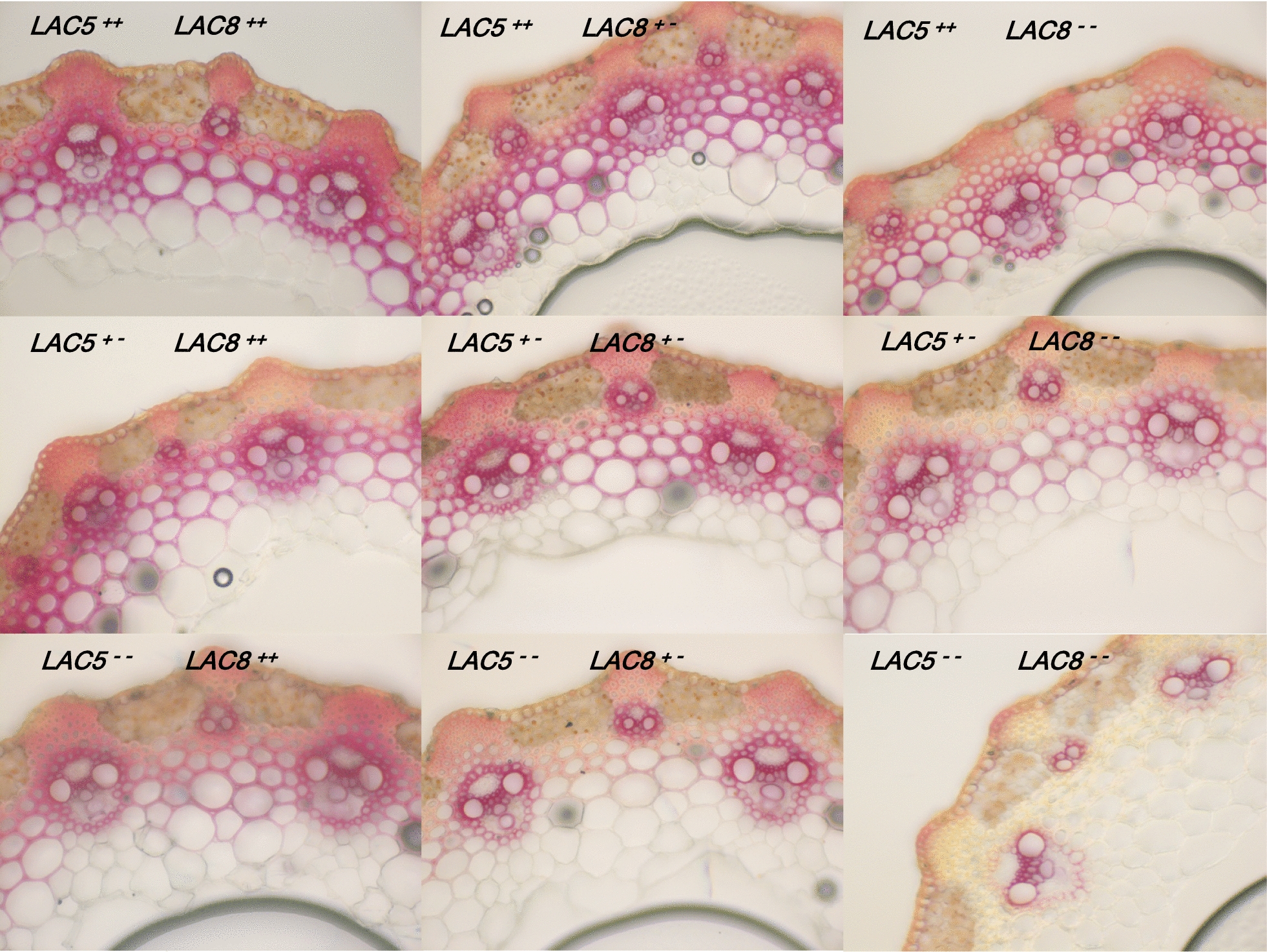
Lignin patterning in different genotyped mutants. Cross sections of dried stems from F2 plants subjected to phloroglucinol–HCl are imaged. Each genotype respective to mutation in *LACCASE5* or *LACCASE8* is indicated. ++: WT homozygous, +−: WT heterozygous, −−: mutant homozygous

**Fig. 4:**
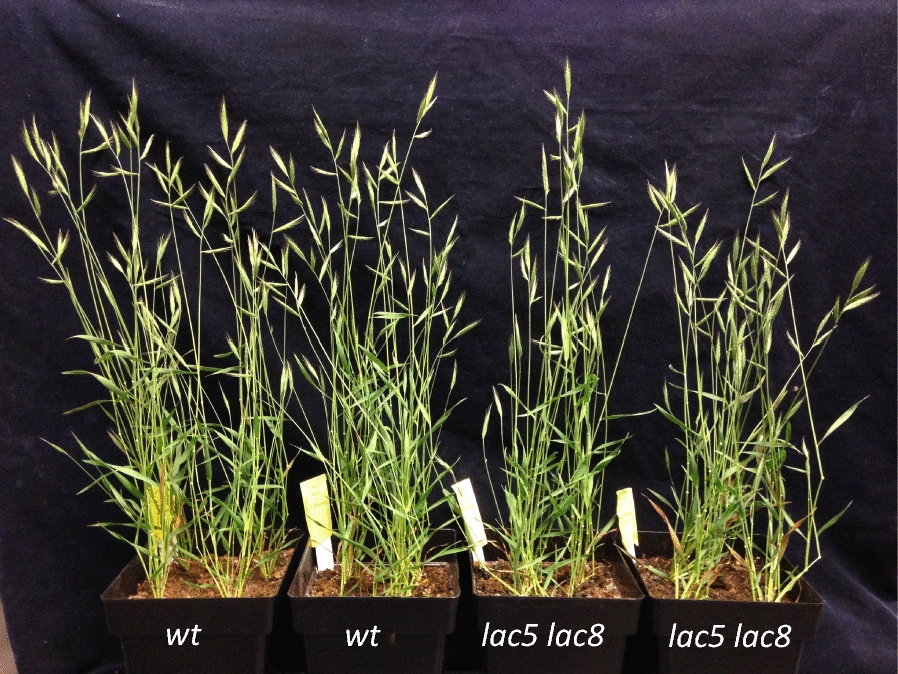
Growth phenotype of WT and of *lac5 lac8* mutant

**Fig. 5:**
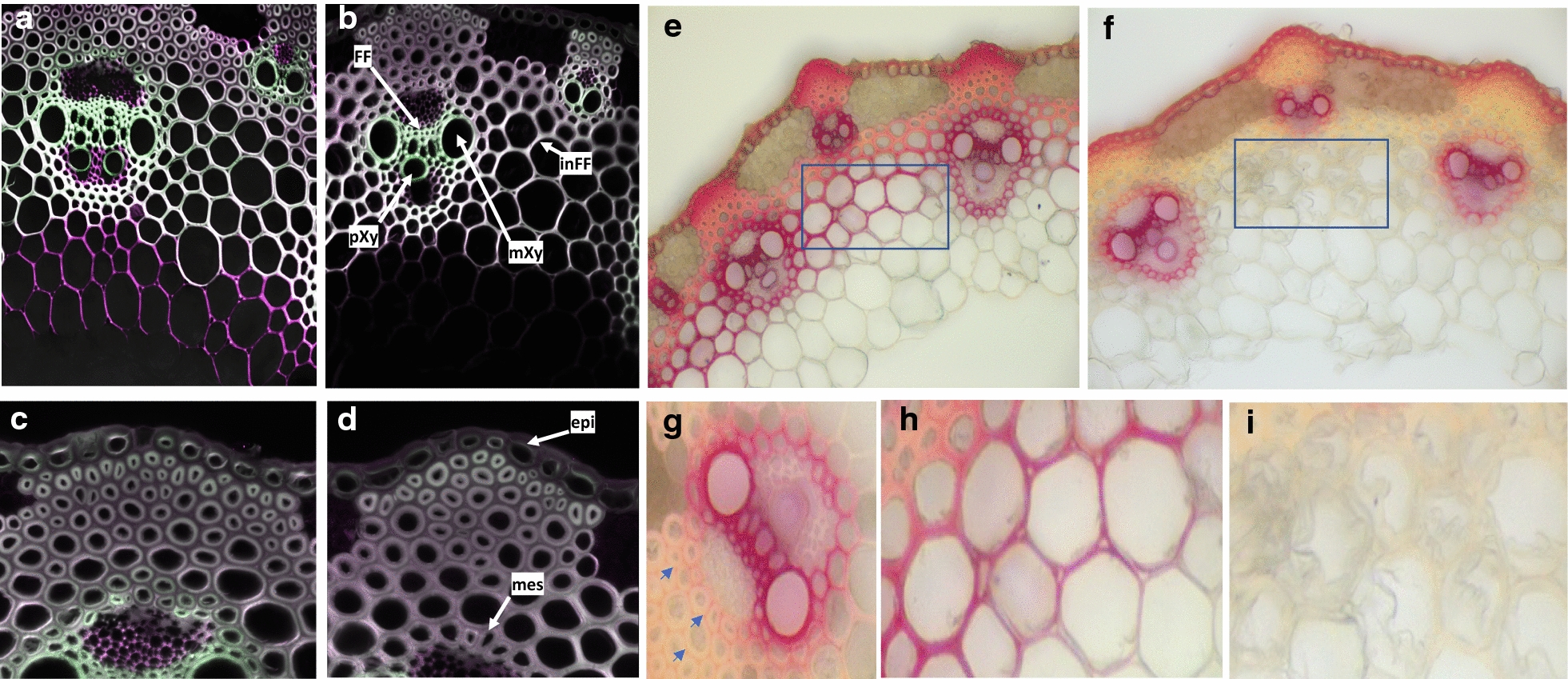
Two-photon fluorescence microscopy imaging and phloroglucinol–HCl staining of WT and *lac5 lac8* lignified tissues. Cross sections of 30-day-old plants were imaged using two-photon fluorescence microscopy (green: 420–460-nm emission, purple: 495–540-nm emission **a**–**d**) or stained with phloroglucinol–HCl prior imaging under visible microscopy (**e**–**i**). WT: **a**, **c**, **e**, **h**; *lac5 lac8*: **b**, **d**, **f**, **g**, **i***. FF* intrafascicular fibers, *pXy* protoxylem, *mXy* metaxylem, *inFF* interfascicular fibers, *epi* epidermis, *mes* mestome. Blue arrows show red staining of primary cell wall between interfascicular fiber cells in the double mutant

To bypass a possible functional genetic redundancy with *LAC5* as suggested by both expression patterns of *LAC5* and *LAC8*, we crossed *lac8*^*5731*^ homozygous line isolated in this study to the previously characterized *lac5*^*4442*^ homozygous line [15]. We genotyped 136 F2 plants from the resulting F1 generation using high-resolution melting (HRM) qPCR. Frequency of both homozygous mutant alleles in F2 progeny revealed a typical 1:16 Mendelian-like segregation. In parallel with HRM qPCR analysis, we generated vibratome cross sections of dried stems from each genotype and performed phloroglucinol–HCl staining. This staining results in the formation of red stain in the presence of lignified cell walls [20]. Of all genotypes, it was rather obvious that the homozygous *lac5 lac8* double mutant (*LAC5*^−^*LAC8*^−^, Fig. 3) showed the most substantial difference with WT (*LAC5*^++^*LAC8*^++^, Fig. 3) while this line did not show a severe growth phenotype when cultivated in the greenhouse or in the growth chamber (Fig. 4). UV excitation fluorescence microscopy has also been used as a sensitive method to document the spatial distribution of lignin in cell walls [21, 22]. Using two-photon UV fluorescence microscopy, we clearly observed that fluorescence at 420–460 nm was impacted in the secondary cell wall of interfascicular fibers (inFF) and mestome (mes) cells in 30-day-old plants (Fig. 5a–d). Surprisingly, fluorescence remained intense inside the fascicular bundle of the double mutant (Fig. 5b). This result suggests that lignins (which emit at 420–460 nm) from protoxylem (pXyl), metaxylem (mXyl) and intrafascicular fibers (FF) are not or moderately impacted. Dried senesced stems (90-day-old plants) treated with phloroglucinol–HCl confirmed these results (Fig. 5e, f). More importantly, while phloroglucinol–HCl staining was almost undetectable in the secondary cell wall of interfascicular fibers of *lac5 lac8*, it was still observed in primary cell wall (Fig. 5g). This result is consistent with the observation that some laccases direct lignification in the secondary cell wall [23] but were not localized to the cell corners/middle lamella in Arabidopsis [24]. In *lac5 lac8* dried stems, the cell walls of interfascicular fibers were observed to be jagged and malformed probably because of the lack of cell strength and rigidity normally imparted by lignification in WT (Fig. 5h, i). We hypothesize that the lignin polymer functions as a ‘cement’ in the cell walls of WT plants and in the absence of this cement in the double mutant, the cell wall polysaccharides collapsed in the inner cell space when the plant is dried and cut.

**Fig. 6:**
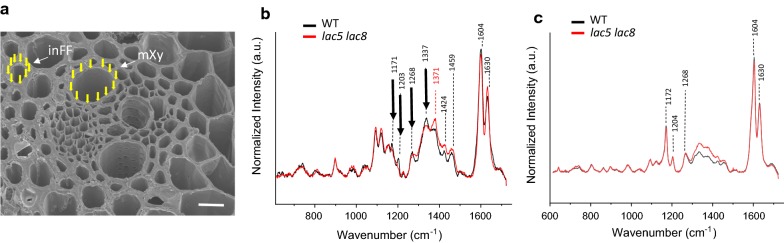
Raman spectroscopy of interfascicular fiber cell wall and metaxylem cell wall from WT and *lac5 lac8*. **a** Scanning electron microscopy of lignified tissues in WT. Yellow arrows illustrate impacts of the laser on cell wall of interfascicular fiber cell and metaxylem. **b** Raman spectral acquisition of interfascicular fiber cell wall in mutant (red line) and WT (black line). **c** Raman spectral acquisition of metaxylem cell wall in mutant (red line) and WT (black line). *inFF* interfascicular fiber, *mXy* metaxylem. Wavelength numbers for major peaks are indicated

In addition to visible microscopy and two-photon UV fluorescence microscopy, we used Raman spectroscopy to characterize changes in the cell wall composition of interfascicular fibers and metaxylem cells (Fig. 6a). We first compared Raman spectra from mutant versus WT interfascicular fiber cells (Fig. 6b). After baseline correction and normalization on standard error, we noticed a substantial decrease in signal at the bands 1171, 1203, 1268 and 1337 cm^−1^ in the double mutant. Interestingly, these bands were attributed to lignin polymers in previous papers. Indeed, based on Raman measurements on standard lignins, 1268 cm^−1^ has been shown to be specific of G-unit lignin type and 1337 cm^−1^ has been assigned to both G and S units [25–28]. The 1203 cm^−1^ band was assigned to OCH_3_ present in both G and S units [26, 29]. The 1171 cm^−1^ band has been assigned to lignins and to esters (C–O–C) [30]. Bands at 1603 and 1630 cm^−1^ were also found slightly changed between mutant and WT. Overall, these signals are often attributed to aromatic compounds and thus to lignin polymers, ferulic and coumaric acids [31, 32].

In the Raman spectroscopy, one new peak at 1370 cm^−1^ was detected for the *lac5 lac*8 mutant, and signals in the vicinity of 1424 and 1460 cm^−1^ were also found increased. These bands are attributable to C–C and C–H, and in consequence, it is difficult to assign them to a specific compound in the cell wall, even if they have been often attributed to lignin, cellulose and hemicellulose [30, 32, 33]. Interestingly, when we compared Raman spectra from metaxylem cell walls of WT versus those of double mutant (Fig. 6c), there was no detectable change in intensity of lignin-related peaks (1171, 1203, 1268 and 1337 cm^−1^) despite the fact that we found again a slight increase in signal in the region of 1330–1460 cm^−1^. These results confirm that lignification is specifically affected in interfascicular fiber cell wall, but it is not drastically affected in metaxylem cells as suggested by phloroglucinol–HCl staining and two-photon confocal analysis.

Consistent with the lignin being unaffected in the vessel secondary cell walls, we did not observe collapsed xylem vessels in the *Brachypodium lac5 lac8* mutant (Fig. 5g), unlike the Arabidopsis *lac4 lac17* double mutant [10] or in Arabidopsis *lac4 lac17 lac11* triple mutants [11].

### Lignin content and structure are strongly affected in the double *lac5 lac8* mutant

Lignin analyses were performed on single and double mutants along with the WT. Both the gravimetric Klason method and the spectrometric acetyl bromide method confirmed that *LAC5* deficiency induced a moderate decrease (about 10%) in the lignin level of *Brachypodium* mature culms, as compared to WT level (Table 1). A similar decrease (about 10%) was observed in the *lac8* sample. By contrast, in the *lac5 lac8* double mutant, the lignin level was found to be more severely reduced relative to the WT (20 to 30%, Table 1, [15]). This reduction provides the clue that, together with *LAC5*, the *LAC8* gene is involved in the lignification of *Brachypodium* stem.

**Table 1:** Lignin content of extract-free mature culms from *Brachypodium* wild type and laccase mutants

Line	KL %	ABL %
WT	18.29 ± 0.09 (100)	20.85 ± 0.27 (100)
*lac5*	16.37 ± 0.10 (89)*	19.50 ± 0.50 (93)*
*lac8*	16.52 ± 0.22 (90)*	19.11 ± 0.42 (92)*
*lac5 lac8*	12.10 ± 0.04 (66)*	16.78 ± 0.46 (80)*

The Klason lignin (KL) and the acetyl bromide lignin (ABL) contents are expressed as weight percentage of extract-free samples. Data are mean values and standard errors from biological triplicates. Values between brackets are the percentages relative to the control level. Asterisks denote significant differences (one-way ANOVA test) relative to the control value at *P* < 0.05

Lignin structure was studied by thioacidolysis. This lignin-specific method provides *p*-hydroxyphenyl (H), guaiacyl (G) and syringyl (S) thioethylated monomers from H, G and S lignin units that are only bound together by β-*O*-4 bonds [34]. When expressed relative to the Klason lignin content, thioacidolysis yields were very similar in all samples, which indicates that the proportion of the target β-*O*-4 bonds is relatively consistent among the various lines (Table 2). Whatever the sample, H monomers were recovered as minor components. Relative to the control value, the molar frequency of G monomers was found to be decreased in all laccase mutants, whereas that of S monomers was concomitantly increased (Table 2). Not unexpectedly, such opposite variations induced dramatic changes in the S/G molar ratio from 1.85, for the control, up to 2.90 in *lac5 lac8* mutant. These variations reveal that the deposition of G lignin units is more strongly affected in the *lac5*- and/or *lac8*-deficient mutants. This slight but systematic decrease in G lignin units, already observed in *Brachypodium lac5* and Arabidopsis *lac17* mutants [10, 15], supports the hypothesis of a spatial and/or temporal specificity of laccase for coniferyl alcohol. These laccases could be more active during the early lignification stages when G units are specifically deposited prior to S units [35].

**Table 2:** Determination of the main H, G and S monomers released by thioacidolysis of extract-free mature culms from *Brachypodium* wild type and laccase mutants

Line	Thioacidolysis yield (H + G + S) in µmol/g KL	Molar frequency %		
		%H	%G	%S
WT	947 ± 18	3.4 ± 0.2	33.9 ± 0.8	62.7 ± 0.9
*lac5*	974 ± 41	2.3 ± 0.1*	25.7 ± 1.8*	72.0 ± 1.9*
*lac8*	910 ± 20	2.5 ± 0.0*	31.4 ± 0.6*	66.2 ± 0.6*
*lac5 lac8*	827 ± 137	3.4 ± 0.2	24.8 ± 1.5*	71.8 ± 1.5*

Data are mean values and standard errors from biological triplicates. Asterisks denote significant differences (one-way ANOVA test) relative to the control value at *P* < 0.05

The amount of CA and FA ester-linked to *Brachypodium* cell walls was measured by mild alkaline hydrolysis. It is now well established that, in grass lignified tissues, most CA esters are linked to lignins while a lower amount is ester-linked to the arabinose substituents of arabinoxylans [36]. The amount of lignin-associated CA esters was reduced in the double mutant (Table 3), a phenomenon that could be related to its lower lignin content. Cell wall FA esters are predominantly linked to the arabinose substituents of arabinoxylans. These FA esters are proposed to act as initiation sites for lignification as first revealed by the identification of FA ether-linked to a G unit [8]. These oxidatively driven and resistant linkages between FA esters and lignins reduce the FA recovery after mild alkaline hydrolysis. On this basis, the dramatic increase in alkali-releasable FA from the double mutant may be directly related to its substantially lower lignin content.

**Table 3:** Determination of *p*-coumaric acid (CA) and ferulic acid (FA) released by mild alkaline hydrolysis of extract-free mature culms from *Brachypodium* wild type and laccase mutants

Line	CA mg/g	FA mg/g
WT	6.01 ± 0.22	5.24 ± 0.26
*lac5*	5.71 ± 0.51	7.86 ± 0.18*
*lac8*	5.08 ± 0.19*	5.88 ± 0.03*
*lac5 lac8*	4.81 ± 0.39*	12.99 ± 0.50*

Data are mean values and standard errors from biological triplicates. Asterisks denote significant differences (one-way ANOVA test) relative to the control value at *P* < 0.05

Because the drastic decrease in lignin content may deeply impact the cell wall’s susceptibility to enzymatic digestion, we performed saccharification assays without pre-treatment. Saccharification efficiency was evaluated both by the weight loss induced by the cellulose hydrolysis treatment and by the amount of glucose released in the reaction medium. As revealed in Table 4, saccharification efficiency was slightly increased in the single *lac5* mutant, whereas it was not changed in the *lac8* sample. By contrast and consistent with the severely reduced lignin content, this saccharification efficiency was substantially increased from the biomass of the *lac5 lac8* double mutant (Table 4).

**Table 4:** Saccharification assays of extractive-free mature stem from *Brachypodium* wild type and laccase mutants

Genotype	Weight loss percentage	Glucose (mg g^−1^)
WT	22.4 ± 1.3	67.4 ± 2.6
*lac5*	28.1 ± 1.3*	97.9 ± 5.8*
*lac8*	20.4 ± 1.0	64.7 ± 0.4
*lac5 lac8*	48.8 ± 0.9*	160.1 ± 26.8*

Saccharification is evaluated both by the weight loss percentage and by the amount of glucose released from the samples

The data represent mean values and standard deviation from 3 to 4 biological replicates. Asterisks denote significant differences analyzed by one-way ANOVA (Tukey’s HSD, *P* < 0.05)

## Conclusion

This work provides the first demonstration that *LAC8* gene is involved in the lignification of *Brachypodium* culm. The impact of loss of function of LAC5 and LAC8 was specifically on the interfascicular fiber cells, which make up the bulk of the grass stem. This was reflected in the substantial increased saccharification yield of the double mutant lines. Cytology studies and Raman spectroscopy showed that lignification of metaxylem cells is not or only slightly impacted in *lac5 lac8* double mutant, which permits these double mutants to continue to grow relatively normally. This result suggests that vascular tissues produce other enzymes that balance the loss of activity of LAC5 and LAC8. At last, close orthologs of the *Brachypodium LAC5* and *LAC8* genes are clearly identifiable in the genome of maize or rice, which confirms that these laccase genes are both promising targets to genetically redesign grass cell walls with improved susceptibility to enzymatic hydrolysis.

## Materials and methods

### Plant material and growth conditions

Single mutants were selected by TILLING in the collection of chemically induced *Brachypodium* mutants as described in [19]. *lac5* (line 4442) and l*ac8* (line 5731) were generated by selfing the initial tilled lines. *Brachypodium* plants (accession *Bd21*-*3*) were grown in a greenhouse under long-day conditions (18-h light, 400-W sodium lamps). Day and night temperatures were 23 °C and 18 °C, respectively.

### Histochemical staining

All the histochemical staining was performed on sections cut in the middle of the second internode from the top. Samples were hand-sectioned or embedded in 7% agarose before being transversely sectioned at a thickness of 50 µm using a vibratome (Leica VT1000S, Leica, Germany). Lignin deposition and composition were investigated histochemically by Wiesner staining (Phloroglucinol–HCl) as previously published [20]. All sections were observed under a Zeiss AxioPlan 2 microscope system with automatic exposure times.

### TILLING and sequence analysis

PCR amplification and detection of mutations DNA amplification are based on nested PCR. The first PCR amplification is a standard PCR with target-specific primers and 10 ng of Brachypodium genomic DNA. Forward primer LCZ23_N1F1: CGCAGTCGCCAACCACACGCTGACGG and reverse primer LCZ23_N1R1: CGTGCAACACCATCGACCCATTCAGC were used. One milliliter of the first PCR product served as a template for the second nested PCR amplification, with a combination of specific primers carrying M13 tail and M13 universal primers, M13F700 (59-CACGACGTTGTAAAACGAC-39) and M13R800 (59-GGATAACATTTCACACAGG-39), labeled at the 5′ end with infrared dyes IRD700 and IRD800 (LI-CORH, Lincoln, Nebraska, USA), respectively. Mutation detection was carried out as described previously except for the second PCR. This PCR was carried out using 0.05 mM of specific primers carrying M13 tail and 0.1 mM of M13 universal primers. The identity of the mutations was determined by sequencing. Sequence analysis was performed with CODDLE software (Codons Optimized to Discover Deleterious Lesions) [37] and PARSESNP software [38]. Prediction of the impact of each mutation was made with SIFT software [39] as described in [19]. Multiple sequence alignment of full-length protein sequences was performed with ClustalW software (http://www.ebi.ac.uk/Tools/clustalw2).

### Lignin content and structure determination

All main stems of each plant were collected and ground after removing spikelets and leaves. Ground samples were sequentially extracted at 60 °C with 50 mL of ethanol, water and ethanol. At each step, the samples were vortexed. These steps were repeated twice before sample drying. The extracted and dried samples, referred to as extract-free samples, were used for lignin analyses.

Lignin content was measured by the Klason method and the acetyl bromide method according to [40]. Lignin structure was studied by thioacidolysis, as previously described [41]. The lignin-derived thioacidolysis monomers were identified by gas chromatography–mass spectrometry as their trimethylsilylated derivatives. All the analyses were performed with at least three biological replicates. Significant differences were inferred by one-way ANOVA (Tukey’s HSD, *P* < 0.05).

### PCR high-resolution melting (HRM) and quantitative PCR

Approximately 20 ng of leaf DNA extract was subjected to PCR amplification on Eppendorf Realplex2 Mastercycler using the SYBR Green kit (Bio-Rad) and using the following conditions: 95 °C for 5 min, followed by 45 cycles of 95 °C for 30 s, 62 °C for 30 s and 72 °C for 30 s. Amplifications were made with the following primers: LAC5fw-CCGGAGGTTGGGTCGCCATCAGGTT and LAC5rev-CATTTACGGTTAAGCAAGAACGTGTGCACG for *LACCASE5* (Bradi1g66320); LAC8fw-GGGCATGCAGGTGTATGGAT and LAC8rev-CGGCAACTTCTGGTTCGGTA for *LACCASE8* (Bradi2g23370). A high melting curve program was used to detect the different alleles with the following program: 95 °C for 10 s, 65 °C for 5 s and increase of 0.2 °C every 5 s until 95 °C. Fluorescence was quantified every 5 s. Relative expression of *LAC8* was quantified as described in [15] using the following primers: qLAC8-fw-TACACGTTCAATGTGACAATGGCG and qLAC8-rev CTCACGCCGTGCCAGTGGAAG.

### Phylogeny tree

An unrooted phylogenetic tree was reconstructed with PhyML in the Phylogeny.fr platform (http://www.phylogeny.fr) [42]. Sequences were aligned with MUSCLE (V3.8.31) and then corrected with Gblocks (V0.91b). The phylogenetic tree was reconstructed using the maximum likelihood method implemented in the PhyML program (v3.1/3.0 aLRT) and graphically edited using TreeDyn (v198.3). Protein homologs from *Brachypodium*, rice, maize and Setaria were identified and downloaded from Phytozome V.12.1 (https://phytozome.jgi.doe.gov/).

### Cell wall saccharification

Saccharification assays were performed as described by [10]. For each sample, 30 mg of extract-free samples was incubated with 4 mL of acetate buffer pH 4.5, containing 4 mg/mL commercial cellulase (cellulase Onozuka-R10; Serva) and 0.5 mg/mL NaN_3_ for 3 days at 45 °C on a carousel. After centrifugation, the glucose content of the supernatant was determined via enzymatic assay with the bioMérieux Kit (bioMérieux, Craponne, France). The pellet was washed twice with water, then freeze-dried and weighed to evaluate the weight loss.

### Raman spectroscopy

Forty-micrometer sections of internodes were cut using a vibratome (HM 650 V, Microm Microtech), then placed with a drop of water on an aluminum slide and sealed with a coverslip and nail polish. Raman spectra were recorded using a microconfocal Raman inVia Reflex (Renishaw), equipped with a double-edge filter to eliminate the Rayleigh scattering with a cut at 100 cm^−1^, three wavelengths (532 nm, 633 nm and 785 nm) and two used different gratings (1800 and 1200 L/mm). The setup consisted of a confocal microscope aligned with an automated XYZ table, where the displacement motors generated 100 nm steps. Then, the focused power of the laser beam was checked for each wavelength to avoid any transformation or heating of the samples. The spectral resolution was smaller than 4 cm^−1^ with a precision better than 0.5 cm^−1^. Several Raman measurements were taken in secondary cell walls of metaxylem cell and sclerenchyma. Three to five biological repetitions per genotype were analyzed. A total of 20–30 measurements were taken for each cell type (metaxylem cell or sclerenchyma). Cosmic ray removal was applied using 4.2Wire software (Renishaw), and spectra were further analyzed using OriginLab Pro software. An average spectrum was obtained for each region of interest, which was baseline-corrected and normalized.

### Scanning electron microscopy (SEM)

Two-millimeter hand-cross sections of 30-day-old WT stems were spray-coated with cobalt using a Cressington 208HR High Resolution Sputter Coater and observed with a Hitachi S-4700 field emission SEM.

### Two-photon fluorescence microscopy

An Olympus FV1000 Multiphoton Laser Scanning Microscope with a tunable MaiTai BB DeepSee (710–990 nm) laser was used to stimulate lignin auto-fluorescence. Excitation (350 to 370 nm) of lignin auto-fluorescence is achieved by the absorption of two 730-nm photons [43]. During imaging, two emission channels (420–460 nm and 495–540 nm) are simultaneously collected. Samples were imaged using the Olympus XL Plan N25X objective.

## Additional files

**Additional file 1.** The laccase protein family in the sequenced Bd21 natural accession. Laccase proteins published in [15] are listed with their respective name regarding the first version (V1.0) or the most recent version (V3.1) of the Bd21 genome sequence.

**Additional file 2.** Predicted LACCASE proteins in *Brachypodium*, maize, rice and Setaria. Putative laccases protein sequences of *Zea mays*, *Oryza sativa*, *Setaria viridis and Brachypodium distachyon* were obtained from proteomic databases available on Phytozome (https://phytozome.jgi.doe.gov/pz/portal.html).

**Additional file 3.** Phylogeny tree reconstructed with *Brachypodium*, maize, rice and Setaria LACCASES. The phylogenetic tree was reconstructed using the maximum likelihood method implemented in the PhyML program. The putative proteins sequences used to reconstruct the tree are available in Additional files 1 and 2. Branch length is proportional to the number of substitutions per site and represents evolutionary distance as indicated by the scale bar. The colored cluster highlights the positions of LACCASE 5 and 8 and their closest orthologs in other species.

**Additional file 4.** Genes co-expressed with *LAC5* (*LAC8* is in bold red). The table containing genes found in the co-expression network was made with the search tool from PlaNet (http://www.gene2function.de/) using *LAC5* gene (Brad1g66720) as a bait.

## Data Availability

All data generated or analyzed during this study are included in the published article and its Additional files. Materials are available from the corresponding authors.
