# Supplementary material for: Inactivation of LACCASE8 and LACCASE5 genes in Brachypodium distachyon leads to severe decrease in lignin content and high increase in saccharification yield without impacting plant integrity
Source: Biotechnol Biofuels. 2019 Jul 15;12:181. doi: 10.1186/s13068-019-1525-5 (PMC6628504; doi:10.1186/s13068-019-1525-5)
Supplement: Supplementary file 1 — Additional file 1. The laccase protein family in the sequenced Bd21 natural accession. Laccase proteins published in [15] are listed with their respective name regarding the first version (V1.0) or the most recent version (V3.1) of the Bd21 genome sequence. [file 13068_2019_1525_MOESM1_ESM.docx]

**Additional file 1**

| Laccase name |  | Gene Locus | | |
| --- | --- | --- | --- | --- |
| (Wang et al., 2015) | | V1.0 |  | V3.1 |
|  |  |  |  |  |
| BdLAC1 |  | Bradi1g10570 | | Bradi1g10567 |
| BdLAC2 |  | Bradi1g24880 | | unchanged |
| BdLAC3 |  | Bradi1g24910 | | unchanged |
| BdLAC4 |  | Bradi1g65100 | | unchanged |
| BdLAC5 |  | Bradi1g66720 | | unchanged |
| BdLAC6 |  | Bradi1g74320 | | unchanged |
| BdLAC7 |  | Bradi2g23350 | | unchanged |
| BdLAC8 |  | Bradi2g23370 | | unchanged |
| BdLAC9 |  | Bradi2g53800 | | unchanged |
| BdLAC10 |  | Bradi2g54680 | | unchanged |
| BdLAC11 |  | Bradi2g54690 | | unchanged |
| BdLAC12 |  | Bradi2g54740 | | unchanged |
| BdLAC13 |  | Bradi2g55050 | | unchanged |
| BdLAC14 |  | Bradi2g55060 | | unchanged |
| BdLAC15 |  | Bradi3g02290 | | unchanged |
| BdLAC16 |  | Bradi3g02300 | | unchanged |
| BdLAC17 |  | Bradi3g22320 | | Bradi3g22317 |
| BdLAC18 |  | Bradi3g45190 | | unchanged |
| BdLAC19 |  | Bradi3g59180 | | Bradi3g59177 |
| BdLAC20 |  | Bradi3g59190 | | Bradi3g59187 |
| BdLAC21 |  | Bradi3g59210 | | unchanged |
| BdLAC22 |  | Bradi4g11830 | | Bradi4g11827 |
| BdLAC23 |  | Bradi4g11840 | | unchanged |
| BdLAC24 |  | Bradi4g11850 | | unchanged |
| BdLAC25 |  | Bradi4g11860 | | Bradi4g11857 |
| BdLAC26 |  | Bradi4g11770 | | unchanged |
| BdLAC27 |  | Bradi4g11780 | | unchanged |
| BdLAC28 |  | Bradi4g39330 | | unchanged |
| BdLAC29 |  | Bradi4g44810 | | unchanged |
