# Supplementary material for: Inactivation of LACCASE8 and LACCASE5 genes in Brachypodium distachyon leads to severe decrease in lignin content and high increase in saccharification yield without impacting plant integrity
Source: Biotechnol Biofuels. 2019 Jul 15;12:181. doi: 10.1186/s13068-019-1525-5 (PMC6628504; doi:10.1186/s13068-019-1525-5)
Supplement: Supplementary file 2 — Additional file 2. Predicted LACCASE proteins in Brachypodium, maize, rice and Setaria. Putative laccases protein sequences of Zea mays, Oryza sativa, Setaria viridis and Brachypodium distachyon were obtained from proteomic databases available on Phytozome (https://phytozome.jgi.doe.gov/pz/portal.html). [file 13068_2019_1525_MOESM2_ESM.docx]

**Additional file 2**

>GRMZM2G336337_P01.zma.30976754/1-572

MTSPSSSSSWLLLLLSCLALALLAADAEVHHHEFVVQETPVKRLCKTHNVITVNGQYPGPTLEVREGDTLVI

NVVNRAQYNVTIHWHGIRQMRTGWADGPEFVTQCPIRPGGSYKYRFTIEGQEGTLWWHAHSSWLRATVYGAL

IIRPRENKTYPFAKPSREVPVILGEWWDANPIDVIREAQRTGGAPNVSDAFTINGQPGDLYKCSQKETTAIP

VKPGETALLRFINAALNHELFVTIAQHRMTVVATDASYTKPFTTSVLMVAPGQTTDVLVTMDQAPARYYVAA

RAYVSGQNVAFDNTTTTAVVEYDCGCASDFGPKIQPAFPSLPAFNDTAAAAAFAAGIKSPGRVRVHESVDEH

LFFTVGLGLFNCEPGQLCAGPNNSTRFTASMNNVSFVFPRTDSLLHAHYFKTPGVFTTDFPAHPPVQFDYTA

QNVSQALWQPVPATKLYPLRFGSVVQLVLQDTSIVTPENHPIHIHGYDFFILAEGFGNFDPEKDVDKFNYVV

PPQRNTVAVPVNGWAVIQFVADNPGVWLMHCHLDVHITWGLAMAFLVEDGYGELQSLEPPPVDLPMC*

>GRMZM2G447271_P01.zma.30983031/1-585

MAISSALPCSSLLMAAAQLMLLASVVVQVQGITRHYDFNVTMANVTRLCATKSIVTVNGQFPGPKIVAREGD

RLIIRVTNQAQHNISLHWHGIRQLRTGWADGPAYITQCPIQTGQSYVYNYTVAGQRGTLWWHAHISWLRATV

YGPLVVLPRPGVPYPFPAPYKEVPVIFGEWWLADTEVVVEQALQLGAGPNVSDAHTINGLPGPLYNCSAKDT

YKLKVKPGKTYMLRLINAALNDELFFSVANHSLTVVEVDAVYVKPFTVDTLPIAPGQTTNVLLAAKPFYPGA

NYYMSAKPYSTARPATFDNTTVAGILEYEYPDAPSSSAASFDKALPLHRPTLPQLNDTSFVGNFTAKLRSLA

TPQYPAAVPRTVDRAFFFTVGLGTHPCPGNATCLGPTNTTQFAAAVNNVSFVLPTRALLHSHFAGLSSGVYS

SDFPVAPLTPFNYTGTPPNNTNVASGTKLMVVPYGANVELVLQGTSILGVESHPLHLHGFNFFVVGQGYGNY

DPVNDPPKFNLVDPVERNTVGVPAGGWVAIRFLADNPGVWFMHCHLEVHTTWGLRMAWLVLDGSLPHQKLLP

PPSDLPKC*

>GRMZM2G320786_P01.zma.30990731/1-583

MQRQRAKMPAGQLSWPLLLGAVLAFGVAASPAQASRNTHYDFVIKETNVTRLCHEKTILAVNGQFPGPTIYA

RKDDVVIVNVYNQGSKNITLHWHGVDQPRNPWSDGPEYITQCPIQPGANFTYKIIFTEEEGTLWWHAHSDFD

RATVHGAIVIHPKRGTVYPYPKPHREVPIILGEWWNADVEQILLESQRTGGDVDVSDANTINGQPGDFAPCS

KADTFKMSVEHGKTYLLRVINAGLTNEMFFAVAGHRLTVVGTDGRYLKPFTVDYIMISPGQTMNMLLEANRA

TNGSANSRYYMAARPFFTNAGLPFDDKNTTAILEYTDAPPAAGPPDFPELPAINDTAAATAYTAQLRSLVTE

EHTIDVPMEVDEHMLVTISVNTLPCGANQTCAGPGGNRLAASLNNVSFVSPTTDILDAYYHSTSGVYDPDFP

NKPPFLFNFTAPNPPQEFWLTKRGTKVKVLEYGTVVEVVFQDTAILGAESHPMHLHGFSFYVVGRGFGNFDK

DKDPATYNLVDPPYQNTVSVPTGGWAAMRFRAANPGVWFMHCHFDRHTVWGMDTVFIVKNGKTPNAQMMPRP

PNMPKC*

>GRMZM5G800488_P01.zma.30994089/1-586

MARLPSTAANFHLAIVLCLLHHLINDYLLADAAIVEHTFHVGNLSVQRLGQSQVITAVNGQFPGPKIEARDG

DTVVVHVVNLSPYNLSIHWHGILQRLSCWADGPNMVSQCPIRPAAGTYTYRFNVTGQEGTLWWHAHVSFLRA

TVYGALILHPAAPSSYPFPTPHGEATILLGEWWNVSVVDVERQALLMGAGPNNSVALTINGLIGAGGGGGSE

NSSSSSSSQYELAVQRGRTYLLRIINAALNYELFFKVANHSFTVVAADACYTDPYHTDVIVIAPGQTVDALM

RADADPGRRYYMAAQVYQSLANATYSAATTALLTYLPDASGSDDEQPTLMPTMPAFNDSATAQSFYASLTGL

LQDGVPLHVDTRMLVTFGLGATPCASAQTLCNRTLGSVAASMNNVSFQLPTTMSLLEAHMRGAPDGVYTRDF

PDRPPVMFDFTGDAMAANRSFMLTSKGTKVKTLRYNETVEVVLQNTAVLGAENHPLHLHGFNFFVLAQGPGN

FNAHRHVRKYNLVNPQQRNTVAVPGGGWAVIRFTADNPGVWIMHCHLDAHLPFGLAMAFEVEDGPTPDAVLP

PPPPDYPRC*

>GRMZM5G814718_P01.zma.31008207/1-608

MATMARSGAVKLCCCTSALLLLCCFLLPGALAEERFYEFVVRGTPVKRLCKTQEIITVNGQFPGPTIEVYSG

DTLAVRAVNLARYNVTLHWHGLRQLRNGWADGPEFVTQCPIRPGGSYTYRFTVEGQEGTLWWHAHSSWLRAT

VHGALIIHPRRGVPYPFPKPHTEAPVILAEWWRRDPIAVLRQSMITGAPPNVSDALLINGQPGDLLPCSSQE

TSIIPVVAGETSLLRIINAAMNTELFVSLAGHRMTVVAADAMYTKPFETDVVLLGPGQTTDVLVTAHAAPGR

YYLAARAYASAQGVPFDNTTATAIFQYKNAPGCPTTSGAGAGAGAGAGTFSGPVGRSSRSSGYLGRAGPQPM

LPFLPAFNDTNTATAFSNRLRSPVPVKVPGPVTQEVFTTVGFGLFNCRPGPFCQGPNNTRFGASMNNVSFQL

PDTVSLLQAHYHHIPGVFTDDFPALPPVFFDFTSQNVPRALWQPVKGTRLYRVRYGAVVQIVFQDTGIFAAE

EHPMHIHGYHFYVLATGFGNYDPRRDAARFNLVDPPSRNTIGVPVGGWAVVRFVADNPGVWLVHCHIDAHLT

GGLAMALLVEDGESELEATVAPPLDLPICVL*

>GRMZM2G164467_P01.zma.31011177/1-586

MAMAISSALPCSLLVAALMLLASVVQVQGITRHYDFNVTMANVTRLCASKSIITVNGQFPGPKIVAREGDRL

VIRVTNHAQHNISLHWHGIRQLRTGWADGPAYITQCPIQTGQSYVYNYTVVGQRGTLWWHAHISWLRATVYG

PLVILPKLGVPYPFPAPYKEVPVIFGEWWLADTEVVIKQALQLGAGPNVSDAHTINGLPGPLYNCSAKDTYK

LKVKPGKTYMLRLINAALNDELFFSVANHSLTVVEVDAVYVKPFTVDTLLIAPGQTTNVLLAAKPSYPGANY

YMSAAPYSTARPATFDNTTVAGILEYELYPDAPRPSASAGSFNEALPLYRPTLPQLNDTSFVGNFTAKLRSL

ATPRYPAAVPRTVDRRFFFAVGLGTHPCPANATCQGPTNTTQFAASVNNVSFVLPTKALLHSHFTGLSSGVY

SPDFPVAPLAPFNYTGTPPNNTNVASGTKLMVVPYGANVELVMQGTSILGVESHPLHLHGFNFFVVGQGYGN

YDPVNDPSKFNLVDPVERNTVGVPAGGWVAIRFLADNPGVWFMHCHLEAHTTWGLRMAWLVLDGSLPHQKLL

PPPSDLPKC*

>GRMZM2G305526_P01.zma.31016578/1-577

MGAAPPSSWLAFILFFGTLVALPQSSHGGGTTRHYTFNVTMKKVTRLCTTRAIPTVNGQFPGPKIVTREGDR

VVVKVLNNVKDNVTIHWHGVRQLRTGWSDGPAYVTQCPIQTGQSFVYNFTITGQRGTLFWHAHVSWMRATLY

GAIVILPKRGVPYPLPVKPYKDVPVIFGEWFNADPETIVAQALQTGAGPNVSDAFTINGLPGPLYNCSSKDT

FKLKVLPGKWYLLRLINAALNDELFFSIANHTLTVVDVDAAYVKPFRTDIVLITPGQTTNVLLRAEPDAGCP

AATHLMLARPYGTGQPGTFDNTTVAAVLEYAPPGHIRSLPLFRPSLPALNDTAFAANYSARLRSLATPDYPA

NVPRAVDRSFFFAVGLGTNPCPVNQTCQGPNGTMFTASMNNVSFNMPTTALLQAHYGSVAGVYTPDFPVAPL

EPFNYTGTPPNNTNVSHGTKVVVLDYNTSVEVVLQATSILGAESHPLHLHGFDFFVVGQGFGNYDSSKDPPK

FNLVDPVQRNTVGVPAGGWVAIRFFADNPGVWFMHCHLEVHTSWGLKMAWVVNDGPLPEQKLMPPPADLPKC

*

>GRMZM2G146152_P01.zma.31023054/1-588

MAGGRRLSPACLLRRLTVALVVLTALPELAAARTRRYTFNVTMATVTRLCVTKSVPTVNGRFPGPRLVVREG

DRLVVQVHNNINSNVTFHWHGVRQLRSGWADGPSYITQCPIRPGQSYAYDFRIVGQRGTLWWHAHFSWLRAT

LYGPLVILPPRGVPYPFPKPHRQVTLMLGEWFNADPEAVIKQALQTGGAPNVSDAYTFNGLPGPTYNCSSAA

GDDTFRLRVRPGRTYLLRLVNAALNDELFFAVANHTLTVVQADASYVKPFAAATLVISPGQTMDVLLTASAS

AAAAAPSSAFAIAVAPYTNTVGTFDNTTAVAAVEYGPHQSAAAPLRSLPLPALPRYNDTAAVANFSAMFRSL

ASARYPARVPRTVDRRFFFTVGLGADPCRSRVNGTCQGPNGTRFAASMNNVSFAMPRTASLLQAHYQRRYSG

VLAANFPAVPPTRFDYTGAPPNNTFVTHGTRVVPLSFNTTVEVVLQDTSVLGAESHPLHLHGYDFFVVGTGF

GNYDATNDTARYNLVDPVQRNTVSVPTAGWVAIRFVADNPGVWIMHCHLDVHLTWGLAMAWLVNDGPLPNQK

LPPPPSDIPRC*

>GRMZM2G367668_P01.zma.31027587/1-583

MGARRGLRRGQAAAAAFSACPFLALAVVLLALPELAAGDTHYYTFNVQMTNVTRLCVTKSIPTVNGEFPGPK

LVVREGDRLVVKVHNHINYNVSFHWHGVRQLRNGWADGPSYITQCPIQGGQSYVYDFTVTGQRGTLWWHAHF

SWLRVHLYGPLVILPKRGEGYPFPRPYKEVPILFGEWFNADTEAVINQALQTGAGPNVSDAYTFNGLPGPTY

NCSSKDTYKLKVKPGRTYMLRLINSALNDELFFGIANHTLTVVEADASYVKPFTVSTLVISPGQTMNVLLTT

APSPASPAYAMAIAPYTNTQGTFDNTTAAAVLEYAPTTTRNNTLPPLPALPLYNDTGAVSNFSRNFRSLNSA

RYPARVPVAVDRHLLFTVGLGTDPCPYTNQTCQGPNGTKFAASVNNNSFFRPRTALLEAHYRRRYAGVLLAD

FPTAPPHPFNYTGTPPNNTFVQHGTRVVPLRFNASVELVLQGTSIQGAESHPLHLHGYNFFVVGQGFGNFDP

VNDPPGYNLADPVERNTISVPTAGWVAVRFLADNPGVWLMHCHFDVHLSWGLSMAWLVNDGPLPNEKMLPPP

SDLPKC*

>GRMZM5G842071_P04.zma.31028130/1-642

MVQLLPALVALALLLVRPVADAAMAKYTFTVGSMQISQLCSSTSIIAVNGQLPGPSIEVNEGDDVVVKVVNN

SPYNVTIHWHGVLQLMTPWADGPSMVTQCPIQPSSSYTYRFSVPGQEGTLWWHAHSSFLRATVYGAFIIRPR

RGNAYPFPAPDKEVPIVLGEWWNRNVVDVESDAILAGQLPAQSDAFTVNGKTGLLYQCANETFTAVVEPSTR

VLLRVVNAGLNSHLFFKLAGHNFTVVAVDAGYTSNLNTDTLVLAPGQTVDALVTTAAAPGSYYMAVLAHDTM

SPLAFAASDTTTATAILQYNGTSSTNPPAMPAMPSSSDSGTANAFYFGLRGLGAPAVPAPVDVSMTIELGLG

QLPCDPSQTRCNGTAAAAAMNGVSFRLPSPETSLLGAHVDGVAGVFTADFPDGPPPSGTAMSVGTKLKKLSY

NSVVEIVLQNPAAVPTENHPIHLHGFNFFVLAQGMGTFAPGSVAYNLVDPVARNTIAVPGGGWAVIRFVANN

PGMWFFHCHLDPHVPMGLGMVFQVDSGTTPGSTLPTPPGDWVGVCDAQHYAAAAAVAAAPVPVPAPAPVPAP

ILAPAPAESPLPPPRAVDHKPSPNLPQRREHTGTSNSAAGRRAKGHLACFLCSVLLFFLLRQHKA*

>GRMZM5G842071_P03.zma.31028131/1-631

MHSTAEHGPPSSRTTPWPMVGSMQISQLCSSTSIIAVNGQLPGPSIEVNEGDDVVVKVVNNSPYNVTIHWHG

VLQLMTPWADGPSMVTQCPIQPSSSYTYRFSVPGQEGTLWWHAHSSFLRATVYGAFIIRPRRGNAYPFPAPD

KEVPIVLGEWWNRNVVDVESDAILAGQLPAQSDAFTVNGKTGLLYQCANETFTAVVEPSTRVLLRVVNAGLN

SHLFFKLAGHNFTVVAVDAGYTSNLNTDTLVLAPGQTVDALVTTAAAPGSYYMAVLAHDTMSPLAFAASDTT

TATAILQYNGTSSTNPPAMPAMPSSSDSGTANAFYFGLRGLGAPAVPAPVDVSMTIELGLGQLPCDPSQTRC

NGTAAAAAMNGVSFRLPSPETSLLGAHVDGVAGVFTADFPDGPPPSGTAMSVGTKLKKLSYNSVVEIVLQNP

AAVPTENHPIHLHGFNFFVLAQGMGTFAPGSVAYNLVDPVARNTIAVPGGGWAVIRFVANNPGMWFFHCHLD

PHVPMGLGMVFQVDSGTTPGSTLPTPPGDWVGVCDAQHYAAAAAVAAAPVPVPAPAPVPAPILAPAPAESPL

PPPRAVDHKPSPNLPQRREHTGTSNSAAGRRAKGHLACFLCSVLLFFLLRQHKA*

>GRMZM2G132169_P01.zma.31028610/1-573

MASSSSSRLLLLLLPCLALTLLGAADAEVHHHEFVIQETPVKRLCKTHNIITVNGQYPGPTLEVREGDTLVI

NVVNRAQYNATIHWHGIRQIRTGWADGPEFVTQCPIRPGGSYKYRFTIQGQEGTLWWHAHSSWLRATVYGAL

IIRPRENKTYPFAKPSREVPVILGEWWDANPIDVIREAQRTGGGPNVSDAFTINGQPGDLYKCSNKETTAIP

VKAGETALLRFINAALNHELFVTIAQHKMTVVATDASYTKPFTTSVLMLAPGQTTDVLVTMDQPPTRYYVAA

RAYVSGQNVAFDNTTTTAVLEYDCGCASDFGPKIQPAFPAALPAFNDTSAATAFAAGIRSPDRVKVHENVDE

YLFFTVGLGLFNCKPGQLCAGPNNNTRFAASMNNVSFVFPQTDSLLHAHYYKIPGVFTTDFPAYPPVQFDYT

GQNVSQALWQPVPATKLYPLRFGSVVQLVLQDTSIVTPENHPIHIHGYDFFILAEGFGNFDPKKDVEKFNYV

DPPQRNTVAVPVNGWAVIQFVADNPGVWLMHCHLDVHITWGLAMAFLVEDGYGELQSLEPPPVDLPMC*

>GRMZM2G132169_P02.zma.31028611/1-517

MTSIVWSVYTLSRSWPAVSINARCMHGVCRHGIRQIRTGWADGPEFVTQCPIRPGGSYKYRFTIQGQEGTLW

WHAHSSWLRATVYGALIIRPRENKTYPFAKPSREVPVILGEWWDANPIDVIREAQRTGGGPNVSDAFTINGQ

PGDLYKCSNKETTAIPVKAGETALLRFINAALNHELFVTIAQHKMTVVATDASYTKPFTTSVLMLAPGQTTD

VLVTMDQPPTRYYVAARAYVSGQNVAFDNTTTTAVLEYDCGCASDFGPKIQPAFPAALPAFNDTSAATAFAA

GIRSPDRVKVHENVDEYLFFTVGLGLFNCKPGQLCAGPNNNTRFAASMNNVSFVFPQTDSLLHAHYYKIPGV

FTTDFPAYPPVQFDYTGQNVSQALWQPVPATKLYPLRFGSVVQLVLQDTSIVTPENHPIHIHGYDFFILAEG

FGNFDPKKDVEKFNYVDPPQRNTVAVPVNGWAVIQFVADNPGVWLMHCHLDVHITWGLAMAFLVEDGYGELQ

SLEPPPVDLPMC*

>GRMZM2G072780_P01.zma.31029630/1-586

MAMAISSALPCSLLVAALMLLASVVQVQGITRHYDFNVTMANVTRLCASKSIITVNGQFPGPKIVAREGDRL

VIRVTNHAQHNISLHWHGIRQLRTGWADGPAYITQCPIQTGQSYVYNYTVVGQRGTLWWHAHISWLRATVYG

PLVILPKLGVPYPFPAPYKEVPVIFGEWWLADTEVVIKQALQLGAGPNVSDAHTINGLPGPLYNCSAKDTYK

LKVKPGKTYMLRLINAALNDELFFSVANHSLTVVEVDAVYVKPFTVDTLLIAPGQTTNVLLAAKPSYPGANY

YMSAAPYSTARPATFDNTTVAGILEYELYPDAPRPSASAGSFNEALPLYRPTLPQLNDTSFVGNFTAKLRSL

ATPRYPAAVPRTVDRRFFFAVGLGTHPCPANATCQGPTNTTQFAASVNNVSFVLPTKALLHSHFTGLSSGVY

SPDFPVAPLAPFNYTGTPPNNTNVASGTKLMVVPYGANVELVMQGTSILGVESHPLHLHGFNFFVVGQGYGN

YDPVNDPSKFNLVDPVERNTVGVPAGGWVAIRFLADNPGVWFMHCHLEAHTTWGLRMAWLVLDGSLPHQKLL

PPPSDLPKC*

>GRMZM2G072808_P01.zma.31029776/1-583

MATPYRLPCCCYALVTVLVLFFSVDATEGAIREYQFDVQMTNVTRLCSSKSIVTVNGQFPGPTVFAREGDFV

VIRVVNHVPYNMSIHWHGIRQLRSGWADGPAYITQCPIQSGQSYVYKFTITGQRGTLWWHAHISWLRATVYG

PIVILPKPGVPYPFPAPYDEVPVLFGEWWTADTEAVISQALQTGGGPNVSDAFTINGLPGPLYNCSAKDTFK

LKVKPGKTYMLRIINAALNDELFFSIAGHPLTVVDVDAVYIKPITVETIIITPGQTTNVLLTTKPSYPGATY

YMLAAPYSTARPGTFDNTTVAGILEYEDPTSSPPPHAAFDKNLPALKPTLPQINDTSFVANYTARLRSLATA

EYPADVPREVHRRFFFTVGLGTHPCAVNGTCQGPTNSSRFAASVNNVSFVLPTTALLQSHFAGKSRGVYSSN

FPAAPLVPFNYTGTPPNNTNVSNGTKLVVLPYGTSVELVMQGTSILGAESHPLHLHGFNFFVVGQGFGNFDP

AKDPAKYNLVDPVERNTVGVPAAGWVAIRFRADNPGVWFMHCHLEVHVSWGLKMAWLVLDGERPNEKLLPPP

SDLPTC*

>GRMZM2G400390_P01.zma.31030770/1-583

MPRSWMILLLVLCSAAVLAQAATVEHTFNVATISWPRICQPGNVSITAVDGVPGPVIEANEGDTVLVHVINH

SPLNVTVHWHGVFQLGTPWADGPSMVTQCPICPGHRYTYRFRITGQEGTLWWHAHSSLLRATVYGALIIRPS

SGSAYPFPAPDEEKTVLLGEWWNAETVPLTNPVADAYTINGRPGDSYSCETTAKRIEKFEVRHDSTYMLRII

NAALNTAFFLKVAGHTFTVVAADASYTTQYETDVIVIAPGQTVDALMVANASPGPGRYYMAISSYQSAAPLR

PGSYNANITTAVVEYVGAAASAGQQAPTPALPEMPEVNDTATANRFYTGMTALVRPGRRTVPLAVDTRMFVT

IGLGFVSCDQDQAPCPVVASMNNQSFKLPDADTMSMLDARYRDTPDGVYTRDFPDQPPVAFDYTNQTERLLL

GGVAAALLFPGPQSTKVRTLAYNATVEVVLQNTALVGRESHPMHLHGFNFFVVAQGFGNYDTAAKQHFNLVN

PQERNTIAVPTGGWAVIRFVADNPGMWFMHCHIDAHLSIGLAMVFEGGYIQSESSNFNLHGNLIFNAYENEC

LKPSKR*

>GRMZM2G309594_P01.zma.31043415/1-592

MASCRRRWPLLLALAQLLVVCGGLGATVEHTFDVRNDLSISQLCQPARAITAVNGRLPGPTIHVREGDTVVV

HVINNSPYNITIHWHGLFQRGTQWADGPEMVTQCPIRPSSRYTYRYNATGQEGTLWWHAHSSMLRATVHGAI

VIKPRNGALGYPFPKPDQERIVMLGEWWNGNVFDLERNAFLTGNLVPQADAYTINGKPGDLHRCSGGSNRRP

RTFKLKVRSGSTYLLRIINAAVNTPMFFKVAGHSFTVVGADAGYTAPYETDVVVVAPGQTVDALMATAAAPP

SRRRYYMMASPYNSARPSLPFRNSTATAVVEYVDVGHQQNKQGPRRRRPVLAPIMPPPNDTATAHRFFTSLA

ALVRPGARAPAVPLAVDTRMLVTIGLGLAACRPEQTRCSGGSRQQVFAGSMNNASFVLPAAMSLLEAHFRNA

TAGVYTRDFPDAPPLEFDYTRPPRGMSLATAKSTRVRTLPYNATVEVVLQDTALVARESHPMHLHGHNFFVL

AQGFGNFRRETAEKQYNLVNPLQRNTLAVPTGGWAVIRFVANNPGMWIMHCHFDAHLPIGLAMAFEVQDGPT

PETALPPPPPDFPQC*

>GRMZM2G140527_P01.zma.31045566/1-638

MVAYLLSKPNGSWKASMKVWVGLPAAAAVVVVFLIFAGVAALPAAMAAVVEHTFVVSQVNLTRLCKETLVTV

VNGQLPGPAIEVTEGDSVVVHVVNRSPYNMTIHWHGVKQRLNCWADGVPMVTQCPILPGRSFTYRFNVAGQE

GTLWWHAHVPCLRATLHGALIIRPRHSPYPFAPKPDREIPVVIGEWWDMDLAQLDRNTMDGFLVDVPTGSTI

NGKLGDLYSCSGAAQDGFVLEVEPGKTYLLRLMNAALFSEYYLKVAGHRMTVVASDANYVRPYTTDVVAIAP

GETMDVLLPADAPPGRSYYMAALAIQAPEPDVQVPPTITRGIVQYRSSSSDVAVDGVVVPAADPAVVMPDMP

DQHDTTISFHFHGNLSSLRRRHRVPPARADDHMLVTLSLGSVCRDGGRACARSDSDESIIVGTMNDVSFRAP

TAAAMPLLEAHYYGRGDMAMAAAAGVELRALPDAPLRVFNFTDPAYIPYGPKEAPLEPTEKQTTVRRFRHGA

VVEVVFQDTAVMQSDSNPMHLHGHDMFVLAHGLGNYDAARDVATYNLLDPPLKNTVVVPRLGWVAVRFVADN

PGTWYIHCHFDFHLSMGMVAVFIVEDGSSADTYLPPPPADLPKCGSNKGGLYLPEEFYLQK*

>GRMZM2G388587_P01.zma.31045883/1-610

MEPVKEGEFEEERRKGQNLLPALHMNSAAPAPIKRAGRPASALLGLLLALHLAIAKEQYHEFVVGMSSQGRR

RPMHFHVKEATVTRLCGTQQQRIMTVNGQFPGPTVEVAEGDALIVRVRNRGSYNVTVHWHGVRQMRTAWADG

PEFVTQCPIRPGTSYTYRFTVAGQEGTLWWHAHSSWLRATVHGALIIRPRAGVPYPFNAGDTPAREITIILG

EWWNMNPIDVVRTATRTGAAPNISDALTVNGQPGDLYKCSSNGTTTFSVTSGETNLLRFINAALNTELFVSL

AGHTMTVVGADASYTKPYATPVILVAPGQTTDVLVTFDQPPSRYYLAARAYASAQGVPFDNTTTTAIFDYGT

GASNSSSPAMPTLPAYNDTATATAFTTSLRGLRAADLPARVDESLFFTVGVGLFNCSSGQTCGGPNNTRFAA

SINNVSFVLPSTTSILQAHYAYAAGGATPAGVFTADFPANPPVRFDYTAPNVSRALWQPVPGTRLVLQGTNV

FAAENHPIHLHGYDFYILAEGFGNFDAAADTASFNMDDPPMRNTVGVPVNGWAVIRFVADNPGVWLMHCHLD

VHITWGLAMAFLVEDGVGELQSLEAPPSDLPIC*

>GRMZM2G169033_P01.zma.31048677/1-591

MPLRQRPTMGGGGGGVAKMPAGQLWLLLLGVLLLAFGVPAQASRNTHYDFVITETKVTRLCHEKTILAVNGQ

FPGPTIYARKDDVVIVNVYNQGYKNITLHWHGVDQPRNPWSDGPEYITQCPIQPGANFTYKIIFTEEEGTLW

WHAHSEFDRATVHGAIVIHPKRGTVYPYPKPHKEMPIILGEWWNADVEQILLESQRTGGDVNISDANTINGQ

PGDFAPCSKEDTFKMSVEHGKTYLLRVINAGLTNEMFFAVAGHRLTVVGTDGRYLRPFTVDYILISPGQTMN

MLLEANCATDGSANSRYYMAARPFFTNTAVNVDDKNTTAILEYTDAPPSAGPPDSPDLPAMDDIAAATAYTA

QLRSLVTKEHPIDVPMEVDEHMLVTISVNTIPCEPNKTCAGPGNNRLAASLNNVSFMNPTIDILDAYYDSIS

GVYEPDFPNKPPFFFNFTAPNPPQDLWFTKRGTKVKVVEYGTVLEVVFQDTAILGAESHPMHLHGFSFYVVG

RGFGNFDKDKDPATYNLVDPPYQNTVSVPTGGWAAMRFRAANPGVWFMHCHFDRHTVWGMDTVFIVKNGKGP

DAQMMPRPPNMPKC*

>Sevir.8G219200.1.p.svi.32632195/1-598

MAKFSMAASNLCVAIVALAAVAAAAVGEAAAVEHTFVVSEMKMTHLCNETLVTVVNGQLPGPAIEVTEGDSV

AVHVVNKSPHNITIHWHGLKQRLNCWADGVPMVTQCPIRPGHNFTYRLNVTGQEGTLWWHAHVSCLRASLHG

AFIIRPRHAYPFPKPDKEIPIVIGEWWSMNLAQLAKNMEDGYYDDSSSATTINGKLGDLYNCSGVVEDGLVL

DVEPGKTYLLRLLNAALYSEYYVKIAGHEFTVVSADANYVRPFTTDVVAIGPGETLDALVVANAIPGRYYMV

AVGGQAPKPDIQIPETRSRATVRYAIGAGNGDEAAPPVAPEMPDQHDFMVSFNFHGNLSSLNRPGSPPVPVT

ADESLFVVLRMGSICRRGRLSCKRSGSKESIIVETMNNVSFQLPAVAAATPLLEELYYDRRRNGTVGGSGLD

QLYTLPDRPARPFNYTDRALIPWGPNEAWLEPTEKAAVARRFRHGAVVDIVFQNAAMMDTDNHPMHLHGHDM

FVLAQGHDNYDTVRDVARYNLVDPPLKNTVLVPRLGWAAVRFLADNPGVWYMHCHYELHVSIGMAAVFIIED

GPTLESALPPPPVDFPKCDQQ*

>Sevir.8G262800.1.p.svi.32632443/1-590

MPHSLFRFLCLARSAPMHRPINREMAAVPGSQRCLVRLLLMASFVLLQALSAHAITRHYKFNVVMRNVTRLC

STKPILTVNGKFPGPTLYAREGDNVLVKVVNHVTHNVTIHWHGVRQIRTGWSDGPAYITQCPIQPGTSFLYN

FTVTGQRGTLLWHAHINWLRATVHGAIVILPKLGVPYPFPTPHKEVVVVLGEWWKADTEVVINQAMQQGVGP

NVSDSHTINGHPGPLSDCASSQDGFKLNVENGKTYMLRIINAALNDDLFFKIAGHKFTVVEVDAVYTKPFKT

ATLLITPGQTTNVLLTADRTAGRYLLSVSPFMDAPVQVDNKTGTATLHYANTISATAPLTLIKPPPQNATTI

VSKFVESLRSLNSEEYPANVPLTVDHSLVFTVGVGVNPCAKCINATRLVGTINNVTFIMPSTPILQAHYYNI

PGVFTDDFPATPPHKFNYTGSGPKNLQTMKGTRVYRLPYNASVQVVLQDTGIISPESHPIHLHGFNFFVVGN

GVGNYNPKTSPSTFNLIDPIERNTIGVPTGGWAAIRFRADNPGVWFMHCHFEVHTSWGLKMVFVVDNGKRPD

ETLIPPPKDLPQC*

>Sevir.8G117600.1.p.svi.32632774/1-571

MARCCWLMLLVLAMVACCGHAATVEHTFDVGNFSISQLCQPATIIAAVNSQLPGPTIHVHEGDTVVVHVVNN

SPYNITVHWHGLFQRGTQWADGPEMVTQCPIRPGSRYSYRYNATGQDGTLWWHAHSSMLRATVHGAIVIKPR

NGDQGYPFPKPDKEEIILLGEWWNRNVFNLEREAFLTGNLVDPADAYTINGKPGNMHKCPGSNRKPRTFKLK

VQSNSTYLLRIISAAVNTPMFFKIAGHSFTVVGADASYTTPYETDVIVISPGQTVDALMVADAAPSRRYYMV

ASPYNSARPNLPFRKGAATAVVEYAGGRKRAAPRRPLLARMPRFNDTATAHRFFSSLTALVRPGQPTVPLAV

DTRMFVTVGLGFADCRPEQTQCKQQVFAGSMNNASFVLPTAMSLLEAHFRNVTGVYTRDFPDRPPLEFDYAR

PPRDMDVTTTKSTKVRTVRYNATVEVVLQNTALVARESHPMHLHGHNFFVLAQGFGNFRQDTAAKRYNLVNP

QERNTLAVPSGGWAVIRFVANNPGMWIMHCHFDAHLPIGLAMAFEVQDGPTPETALPPPPADLPQC*

>Sevir.8G223100.1.p.svi.32632936/1-611

MKFSSSPAASAAIAIAVVFFLSAKAPPSANAAVVEHTFVVSQVKMTHLCNETLVTVVNGQFPGPAIEVTEGD

SVAVHVVNKSPHNLTIHWHGVKQRMNCWADGVPMITQCPILPNQSFTYRFNVAGQEGTLWWHAHVSFLRATL

HGALVIRPRRGSSSYPFPKPYMEIPIIIGEWWQMDLFKADWGIKQHKIDAYYTASTINGKLGDIYNCSGAVE

DGYKLDVEPGKTYLLRVINAALFAEYYLKIAGHKFTIVAADANYVTPYTTDVIAIAPGETVDALVVADAAPG

RYYMVALPNQSPKPDPQSPVLITRGIVQYSNKQKPGNGVEHPSFDIPVSPEMPDQHDLMPSFYFHGNLTSLV

HHPQRPRVPTRVDERLFITLGLGSICRQGHSSCGRSESDDSLIVATMNNVSFQQPALEMPLLEMHYYNPDRV

ATMLQELPNKPPMVYNYTDPALIPLGPKEAKLEPSSEATVARRFRQGSVVEVVFQGTALLQSESNPMHLHGH

DVFVLAQGEGTYDAGRDAARYNLVNPPIKNTVHVPRLGWVALRFVADNPGIWYMHCHFGFHLSMGMLALFIV

EDGRTVGTSLPAPPADFPTCGHDHKLMPTELYPI*

>Sevir.8G223200.1.p.svi.32633036/1-582

MAIGVVFFLYAIVPPAAMAAVVEHTFVVSQVKMTHLCNETLVTVVNGQFPGPAIEVTEGDSVTVHVVNESPY

NLTIHWHGVKQRLNCWADGVPMITQCPILPNQNFTYRFNVAGQEGTLWWHAHVSFLRASVHGALIIRPRRGA

SSYPFPKPYKEIPIIIGEWWQMDLARADWAIAHSVDVYFGASTINGKLGDLYNCSGALEDGYMLDVEPGKTY

LLRIVNAALFAEYYLKIAGHKFTVVAADANYVSPYTTDVIAIAPGETVDALVVADAAPGRYYMVALPNQSPE

PDPQSPVLITRGILQYRNVQEALGNSSSSEMAVAPEMPDDQDLLTSFYFHRNLTSLHHPQRPLVPTRVDEHL

FITLSLGSICRRGQSCKRGGSDETFTVATMNNVSFQQPAVATPLLELHYYNTNSMDMLQDLPDKPPMVFNYT

DQALIPPGPKEAKLEPTSKATVARRFRQGSVVEVVFQGTALLQSESNPMHLHGHDVFVLAQGEGLYDAARDT

AWYNLVNPPIRNTVHVPRLGWVAIRFVADNPGIWYMHCHFEFHMTMGMVGLFIVEDGPTVDTSLPAPPADFP

SCGHI*

>Sevir.8G224800.1.p.svi.32633487/1-614

MKWRSLPAGTVAIATGVIFFLSAIGAPGAPAAMASVVEHTFVVRQVKMTHLCKETLATVVNGQFPGPAIEVT

EGDSVTVHVVNQSPYNLTIHWHGVKQRLNCWADGVPMITQCPILPNQNFTYRFNVAGQEGTLWWHAHVSFLR

ASVHGALIIRPRRGASSYPFPKPYKEVPIIIGEWWDMDLLKADWGIQQHVIDPYFNASTINGKLGDLYSCSG

AVEDGYLLDVEPGRTYLLRIINAALFAEYYLKIAGHKFTVVAADANYVSPYTTDVIAIAPGETMDALLVADA

DPGRYYMVALPNQSPLPDPQGPTLITRGIVQYSNKQRAADGGGRPSSDIAVSPEMPDQHDMITSFYFHGNLT

GLHHPQHLEVPKHVDERLFITLGLGSICRGGQSSCKRSENNESLDVATMNXFXYQQPAVATPLLELHYYXXD

NXVLSMLQELPDKPPRVFNYTDPXLIXPGPKEAKLEPTSKATIARRFRQGAVVEVVFQGTAILSSESNPMHL

HGHDMFVLAQGEGNYDATRDVPRYNLVNPAVKNTVFVPRLGWVAVRFIADNPGIWYMHCHFEFHVSMGMVAL

FIVEDGSTVDTFLPAPPADFPTCGHDHNVMSNEFYPI*

>Sevir.8G223000.3.p.svi.32633800/1-611

MKWRSLPAATVAIATGVVFFLSAIGAPAAMASVVEHTFVVRQVKMTHLCKETLATVVNGQFPGPAIEVTEGD

SVTVHVVNQSPYNLTIHWHGVKQRLNCWADGVPMITQCPILPNQNFTYRFNVAGQEGTLWWHAHVSFLRASV

HGALIIRPRRGASSYPFPKPYKEIPIIIGEWWEMDLLKADWGIKQHVIDAYFNASTINGKLGDLYSCSGAVE

DGYLLDVEPGRTYLLRIINAALFAEYYLKIAGHKFTVVAADANYVSPYTTDVIAIAPGETVDALLVADADPG

RYYMVAVPNQSPLPDPQSPTLITRGIVQYSNKQRAADGGGRPSSDIPVSPEMPDQHDMITSFYFHGNLTGLH

HPQHLEVPKHVDERLFITLGLGSICRGGQSSCKRSENNESLDVATMNXFXYQQPAVATPLLELHYYXXDNXV

LSMLQELPDKPPRVFNYTDPXLIXPGPKEAKLEPTSKATIARRFRQGAVVEVVFQGMAILSSESNPMHLHGH

DVFVLAQGEGNYDATRDVPMYNLVNPAVKNTVFVPRLGWVAVRFIADNPGIWYMHCHFGFHLSMGMVALFIV

EDGSTVNTSLPAPPADFPTCGHDHNVMSNEFYPI*

>Sevir.8G016500.1.p.svi.32633835/1-586

MRASGGMSSSSSMPFCSSSLLLITLPLLLWSSLTIQPAIAKEQYHEFVIQEASVTRLCREHSIMTVNGQFPG

PALEIDEGDSLIVRVINRGRYNVTVHWHGVRQMRTGWSDGPEYVTQCPIRPGQSYTYRFTVAGQEGTLWWHA

HSSWLRATVHGALIIRPRAGVTAYPFDAAGEPPAREIPILLGEWWDMNPIDVVRTATRTGAAPNISDALTVN

GHRQRCSSKDTXTFPVKSGETNLLRFINAALNTELFVSLAGHTMTVVGADASYTKPYSTSVLMIAPGQTTDV

LVTFDQPPGRYYLAARAYASAQGVPFDNTTTTAIFDYSSSSSSGSRSPAMPTLPAYNDTATATTFTTNLRGL

RKAELPSRVDENLFFTVGVGLFNCSSGQKCGGPNNTRFAASMNNVSFVLPSTVSILQAHYGGAQAQQGVFTD

DFPANPPVQFDYTAQNVSRALWQPAPGTKVYRLKYGAAVQLVLQGTNIFAGENHPIHIHGYDFYILAEGFGN

FDAATDTAKFNLDDPPMRNTVGVPVNGWAAIRFVADNPGVWLMHCHLDVHITWGLAMAFLVEDGVGELQSLE

APPPDLPLC*

>Sevir.8G221000.1.p.svi.32634224/1-629

MQTMERRSFPALVAATVILFLSATAQPAAGAIVEHTFIVGQMNMTHLCKEMLVAVVNGQLPGPVIEVTEGDT

VVVHVVNKSPYNITIHWHGVKQQLNCWADGVPMITQCPIGPNNNLTYTFNVTGQEGTLWWHAHVAYLRGTLH

GAIIIRPRHGVSSYPFPEPHREIPIVLGEWWQMDLLKASMDIKDSTADDDPSAATINGKLGDLYNCSGVKED

GFALDVEPGKTYLLRIINAALFYEYYIRIAGHKFTVVAADANYVSPLTTDLLAVAPGQTLDALVVANAAPGR

YYMVASPNQPPKPDFQHPTFTTRGIVQYTNENHSNGGSSGPGAEGPLSRNLPVVAPEMPDEHDTMTSYYFHG

KLTALHPSRSLPVPARVDEHLLIALGLGSVCKKRGQSCKRGESEETLTLATMNNISFELPAATAAGPLLEAH

YYNTGSLDMLRELPDRPPRMYNFTDPSFIPSGPREASLEATSKATIVRRFRHGTVVEVVFQSTAMWQSDSNP

MHLHGHDMFVLAQGLGNYNAATDVAKYNLVDPPVRNTVLVPRLGWVAVRFVANNPGAWFVHCHFSFHLSMGM

AAVFVVEDGPTVNTSLPPPPADFLTCYRKNNPVADEFDIRSTKSEIPDVTGA*

>Sevir.8G221600.1.p.svi.32634857/1-603

MAATAAIIFFLSAMAISVRAAVVEHTFVVSQVNMTHLCKETLVTVVNGQLPGPVIEVTEGDSVAVLLVNKSP

YNITIHWHGVKQWLNCWADGVPMVTQRPILPNHNFTYRFNVTGQEGTLWWHAHVTCLRATLHGAIIIRPRDG

ASSYPFPNPDKDVPIIIGEWWEMDLDELDRRMRNSLFDDNPSGATMNGKFGDLYNCSGAKEDGYVLNVEPGK

TYLLRIINAVLFSEYYLKIAGHKFTVVAADANYVNPYTTDIISIAPGETIDALVVADAPPGGYYITALANQS

PKPDPQIPKLVTRGTVWYSTGHVSSNGTAPPVAPEMPDQHDTMASFYFHGNLTSLNDTQRLLVPERADESLF

ITVGLGSICRRGQTCKRSGNNESILVATMNNISFQLPDMSMPLLEAHYYHTGDMDMLQELPDRPPMVFNFTD

RGLIPWGPKEGQLEPTSRGSLVRRFQHGAVVDIVFQGTAVMQSDSNPMHLHGHDMFVLAQGVGNYDAARDVA

KYNLVDPPVRNSVLVPRIGWAAIRFVANNPGVWLMHCHYEFHFSMGMAVIFIVEDGPTVGTSLPPPPLEPAC

NHGHHVVPNDLYLKTTETEFAGVNEV*

>Sevir.8G221600.2.p.svi.32634858/1-603

MAATAAIIFFLSAMAISVRAAVVEHTFVVSQVNMTHLCKETLVTVVNGQLPGPVIEVTEGDSVAVLLVNKSP

YNITIHWHGVKQWLNCWADGVPMVTQRPILPNHNFTYRFNVTGQEGTLWWHAHVTCLRATLHGAIIIRPRDG

ASSYPFPNPDKDVPIIIGEWWEMDLDELDRRMRNSLFDDNPSGATMNGKFGDLYNCSGAKEDGYVLNVEPGK

TYLLRIINAVLFSEYYLKIAGHKFTVVAADANYVNPYTTDIISIAPGETIDALVVADAPPGGYYITALANQS

PKPDPQIPKLVTRGTVWYSTGHVSSNGTAPPVAPEMPDQHDTMASFYFHGNLTSLNDTQRLLVPERADESLF

ITVGLGSICRRGQTCKRSGNNESILVATMNNISFQLPDMSMPLLEAHYYHTGDMDMLQELPDRPPMVFNFTD

RGLIPWGPKEGQLEPTSRGSLVRRFQHGAVVDIVFQGTAVMQSDSNPMHLHGHDMFVLAQGVGNYDAARDVA

KYNLVDPPVRNSVLVPRIGWAAIRFVANNPGVWLMHCHYEFHFSMGMAVIFIVEDGPTVGTSLPPPPLEPAC

NHGHHVVPNDLYLKTTETEFAGVNEV*

>Sevir.8G222700.1.p.svi.32635180/1-599

MDSRSLRVAATAALIFFSVIAQSAGTAIVEHTFVVTQVTMTHLCKDTLVTVVNGQLPGPAIEVTEGDSIAVH

VVNKSPFNLTIHWHGVRQMLNCWADGVPMITQRPILPDHNFTYRFDVSGQEGTLWWHAHVPCLRATIHGILI

IRPRHGAISYPFPKPHKEIPIIIGEWWDLEDLGQVDRHLRHYVADDYFKASTINGKLGDLYNCSGVVEEGYK

LDVEPGKTYLLRVLNAALFSEYYLKVAGHKFRVVAGDANYVSPYTTDIIAIAPGQTFDALVVADASPGRYYM

VAMPNQAPKPDYQSPVLPTRGILQYSNGAGGNQPGDVPMSPEMPDNHNHMLSFYFHGNLTSLHHPRHLPVPK

RIDERLFITLGLGSVCRQGQSCERGAESDEVIVVATMNNISYELPTVSRPLLEAHYQNPSNIDWLQELPDVP

PRVFNFTDSSLIPTGPKEEQLEPTSKAALARRFRYGAVVDVVFQSTSMLQSESNPMHLHGHDMFVLAQGSGN

YDKERDVAKYNLVNPPLVNTVLVPRLGWVAVRFIADNPGVWYMHCHFEFHQSMGMIALFIVEDGPSANTSLP

SPPVDFLTYGDDNNLMPDEYYL*

>Sevir.8G212200.1.p.svi.32635209/1-617

MKIQCLSLPIAATAVASAVIVVLSAMALLGAATAVVEHTFVVSEMNMTHLCKEMLVTVVNGQFPGPMIEVTE

GDSVVVHVVNKSPHNITIHWHGVKQLLNCWADGVPMITQSPILPNQNFTYRFSVTGQEGTLWWHAHVTCLRA

TLHGAFIIRPRHGASSYPFPNPDKEVPIIIGEWWEMDLDELDRRMRDSFFNDNPSGATMNGKLGDVYNCSGA

KEDGYVLNVEPGKTYLLRIINAALYSEYYIKIAGHKFTVVATDANYVNPYTTDIITIAPGETIDALVVADAP

PGGYYIIALANQSPKPDHQMPKFVTRGTVWYSTRHVSSNGTAPPVAPEMPDQHDTMATFYFHGNLTSLNDTQ

QPLVPERADESLFITVGLGSICRRGETCKRSGSNEAIIVATMNNVSFQLPDTNMPLLEAHYYHTGGMDVLQE

LPDRPPMVFNFTDRGLIPWGPKEGQLEPTSRGSLVRHFRHGAVVDIVFQGTAVMQSASNPMHLHGHNMFVLA

QGVGNYDAARDVAKYNLVDPPVRNSVVVPRIGWAAIRFVADNPGVWFMHCHYEFHLSMGMAAIFIVEDGPTV

ETSLPPPPLELLACNHGHHVVPNELYHKTTETEFARVNEV*

>Sevir.2G003200.1.p.svi.32636529/1-570

MHHTPMAQPRPLLLLRLAFALCFLLIRCMLADAAIVEHTFNVGNLTVERLGRSQVITAVNGQFPGPKIEARD

GDTVVVHVVNNSPYNMSIHWHGILQRQSSWADGPNMVSQCPIRPGGGRYTYRFNVTGQEGTLWWHAHVSFLR

ATVYGALLLRPAPAEGYPFDKPHREATILLGEWWNASVVDVERQALLAGGAPNNSVALTINGLVDGDHQLLT

VERGRTYLLRIVNAALNYQLFFKVAAHSFTVVAADACYTDPYHTDVIVVAPGQTVDALMRADAHPGRYYMAA

QVYQSLANATYSATATALITYYHQDDATPPEMPSMPAFNDSATAERFYAGLTGLLRDSTPTVPPHVDTRMLV

TFGLGVTPCAPEQTLCNRTLGSVAGSMNNVSFQFPAAMSLLEAHMRGDPDGVYTREFPDRPPVMFDFSGEAG

AGAAFAFTSKGTKVKALRYGETVEVVLQNTAILGAENHPLHLHGFNFYVLAQGAGNFNAHRHVRAYNLVNPH

QRNTVAVPTGGWAVIRFTADNPGVWIMHCHLDSHLPFGLAMIFEVDDGPTPDAVLPPPPPDYPRC*

>Sevir.4G294600.1.p.svi.32647086/1-594

MGAVAERPAVLLRFLQLGVFLVLGVAALSPAAHGSRVRRYHFLVKKVVVTRLCRQKSILTVNGQFPGPTIRA

RSGDVVVVNVRNHGDKNITIHWHGVDQPRNPWSDGPEYITQCPIQPGAAFAYRVILSQEEGTLWWHAHTGFD

RATVHGAIVILPKHGAAFPFDHPRRVEEMPPIILNEWWRDDDANHLLEEAVRTGRDVKPSDAATINGEPGDM

FPCSEAGTFRARVERGGTYLLRVINAGLTNDVFFAVAGHRLTVVATDARYTKPFAADHLMVASGQTVDALLH

ANCAAGGRYYMAARTFASNTNVVEFNNSTATAILEYADAARGRAAAPVFPATLPAVEDMAAATTYTKRLRSL

ASEAHPVDVPARADERLLVTMAVNLIPCAPDAACTGPRGDRLAASLNNVSFQNPGAVDILSAYYYRDGSSAG

GVYDAGFPDGPPSRFNFTDPGLPEAGLVGPFTVRGTRVKVLEHGAAVEVVFQDTAVLGMESHPMHLHGYSFY

VVGRGIGNFDDGRDPAGYNLVDPPRQNTVAVPKGGWAAIRFRATNPGVWFMHCHFDRHVVWGMDTVFIVKNG

KNPEAKMLRPPPNMPKC*

>Sevir.9G440800.1.p.svi.32654558/1-609

MARSGLVKLLCSSAFLLLCCFLLRGALAEERFYEFVVQETPVKRLCSSQKIITVNGQFPGPTIEVYNGDTLA

IKAVNLARYNVTLHWHGLRQLRNGWADGPEFVTQCPIRPGGSYTYRFTIQDQEGTLWWHAHSSWLRATVHGA

LIIHPRRGLPYPFPKPHSEFPVILAEWWRRDPIAVLRQSMITGAPPNVSDALLINGQPGDLLPCSSQETSII

PVVAGETSLLRIINAAMNTELFVSLAGHKMTVVAADAMYTKPFETTVILLGPGQTTDVLVTAHAAPGRYYLA

ARAYASAQGVPFDNTTATAIFQYKNAPGCPTTTASAGAGAGAGAGMGGNTFNGPVGRSSRSSGHPGRTGPQP

MLPFLPAFNDTNTATAFSNSLRSPHPVKVPGPVTQELFTTVGFGLFNCHPGPFCQGPNNTRFGASMNNVSFQ

LPNTVSLLQAHYHHIPGVFTDDFPSFPPVFFDFTSQNIPRPLWQPVKGTKLYRVRYGAVVQIVFQDTGIFAA

EEHPMHIHGYHFYVLATGFGNYDPRRDAAKFNLVDPPSRNTIGVPVGGWAVVRFVADNPGVWLVHCHIDAHL

TGGLAMALLVENGGSELEATMAPPLDLPICVL*

>Sevir.9G457500.1.p.svi.32655128/1-571

MEAPCLALLLFFGTLLVLPQSSHGATRYYTFNVTLQKVTRLCTTRAIPTVNGKFPGPKIVTREGDRVVVKVV

NSVKDNVTIHWHGVRQLRTGWSDGPAYVTQCPIRTGQSYVYNFTITGQRGTLFWHAHVSWMRATLYGPIIIL

PKRGVPYPFPVKPYKEVPIIFGEWFNADPEAIIAQALKTGAGPNISDAFTINGLPGPLYNCSSKDTFKLKVL

PGKWYLLRLINAALNDELFFSIANHTLTVVDVDAAYVKPFHTDVVLITPGQTTNVLLRAEPDAGCPAATHLM

LARPYGTGQPGTFDNTTVAAVLEYAPPGHIKSLPLFRPSLPALNDTAFAANYSARLRSLATPDYPANVPRGV

DRSFFFAVGLGTNPCPANQTCQGPNGSMFTASMNNVSFDMPTTALLQAHYNNIAGVYTTDFPVAPLEPFNYT

GTPPNNTNVSNGTKVVVLQYNTSVEVVLQDTSILGAESHPLHLHGFDFFVVGQGFGNYDSSKDPAKFNLVDP

VQRNTVGVPAGGWVAIRFFADNPGVWFMHCHLEVHTSWGLKMAWVVNDGPLPEQKLMPPPADLPMC*

>Sevir.1G221300.1.p.svi.32667733/1-580

MGGVAKVPPALWLLLGVVAVAFGVAATPAQASGTNHYDFFIKETNVTRLCHEKAVLAVNGQFPGPTIYARRG

DVVVVNVHNQGHKNITLHWHGVDQPRNPWFDGPEYITQCPIRPGANFTYTIILSDEEGTLWWHAHSDFDRAT

VHGAIVVHPKLGSTYPYPKPHKEIPIILGEWWNVDVEQLLEEMKRTGGDVNISDANTINGQPGDMFPCSRNG

TFRAVVEHGKTYLLRIINAGLTNDMFFAVAGHRLTVVGTDGRYLKPFAADHVMIASGQTVDALLHAGRAPNG

GGRYYMAARTFQTNLQLSINNSTATAVLEYTDAAGPPELPVLPAVRDIGAATAYTARLRSLASEAHPADVPA

RVDERMLVTVSVNVLPCGGGANSNETCDGPINGTRLSASLNNVSFVSPAVDVLDAYYSSIAGVYEPDFPDRP

PVAFNFTDSEPAQELWFTRRGTKVKVVEYGAVVEVVFQGTGILGAEPHPIHLHGYAFYVVGRGFGNFDESKD

PAAYNLVDPPYQNTVSVPAGGWAAIRFRAANPGVWFMHCHFDRHTVWGMDTVFIVKNGKTPDAQMLPRPPSM

PKC*

>Sevir.1G326800.1.p.svi.32667978/1-570

MPSGHCWLLLGLVLAFVVAASPAQASRANHYDFFIKETKITRLCHETTILAVNDQFPGPTIYARKGDVVVVN

VYNQGNKNITLHWHGVDQPRNPWFDGPEYITQCPIQPGANFTYRIIFSEEEGTLWWHAHSDYDRATVHGAIV

IHPKRGSAYPYPKPHKEIPIILGEWWNADAEKLFEETKRGGDVNISDANTINGQPGDQFPCSKNGTFRMLVE

HGKTYLLRVINAGLTNDTFFGVAGHRLTVVGTDGRYLKPFTVESIMISPGQTMNALLEADRPTDGSGNSRYY

MAARTFTGNGLQLKSGNATAILEYTDAPPSAGPPDFPNLPTDDNAAATAYTAQLRSLVTKDHPVDVPTHIDE

HMLVTIAMNFLPCGTNDTCTSPIGSRLAASLNNVSFVAPSVDVLDAYYYSIQGVYEPDFPNNPPFILNITDS

LPLNLSFTKRGTKVKVVEYGAVVEVVFQDTGILAAESHPMHLHGFSFYVVGRGFGNFDKNKHPATYNLVDPP

YQNTVSVPKAGWAAIRFRAANPGVWFMHCHFERHTVWSMHTVFIVKNGRTPDARMMPRRSTMLKC*

>Sevir.5G383300.1.p.svi.32668464/1-579

MGARHGLRRGHAAAASTCPFLAFAVLLALPALAAGDTHYYTFNVQMTNVTRLCVTKSIPTVNGKFPGPKLVV

REGDRLVVKVHNHINYNVSFHWHGVRQLRNGWADGPSYITQCPIQGGQSYTYDFTITGQRGTLWWHAHFSWL

RVHLYGPLVILPKRAEGYPFPRPYKEVPILFGEWFNGDTEAIINQALQTGGGPNVSDAYTFNGLPGPTYNCS

AQDTYKLKVQPGRTYMLRLINSALNDELFFAIANHTLTVVEADANYVKPFTVQTLVISPGQTMNVLLTTAPN

PASPAYAMAISPYTNTQGTFDNTTAAAVLEYAPTPAAAARSLPLPALPLYNDTGAVTNFSRNFRSLASAQYP

ARVPLAVDRHLLFTVGLGTDPCPSNQTCQGPNGTKFAASINNNSFFRPRSALLEAHYQRRYAGVLLANFPTT

PPHPFNYTGTPPNNTFVQHGTRVVPLRFNTSVELVLQGTSIQGAESHPLHLHGYDFFVVGQGFGNFDPVNDP

PTYNLADPVERNTISVPTAGWVAIRFFADNPGVWLMHCHFDVHLSWGLSMAWLVNDGPLPNQKMLPPPSDLP

KC*

>Sevir.5G382900.1.p.svi.32668506/1-583

MAMAISSALRPCSLLVAALMLLASIVEVQGITRYYDFNVTMANVTRLCSSKSIVTVNGQFPGPELVAREGDR

VVVRVTNHAQHNISLHWHGIRQLRTGWADGPAYITQCPIQTGQSYVYNFTVVGQRGTLWWHAHISWLRATVY

GPIVILPKLGVPYPFPAPYKEVPVIFGEWWQADTEVVIKQALQTGGGPNVSDAHTINGLPGPLYNCSAKDTY

KLKVKPGKTYMLRLINAALNDELFFSIANHSLTVVEVDAVYVKPFTVDTLLIAPGQTTNVLLTAKPFYPGAN

YYMSAAPYSTTRPGTFDNTTVAGILEYEYPDAPSSAPSFNKALPLYKPTLPGFNDTDFVGNFTAKLRSLATA

QYPAAVPKTVDKKFFFTVGLGTHPCPANTTCQGPTNTTQFAASVNNVSFALPTRALLHSHFTGLSSGVYSSD

FPVAPLTPFNYTGTPPNNTNVANGTKLMVIPYGTNVELVMQGTSILGIESHPLHLHGFNFFVVGQGYGNYDP

VNDPAKFNLVDPVERNTVGVPAGGWVAIRFLADNPGVWFMHCHLEVHTTWGLRMAWLVLDGSLPHQKLLPPP

SDLPKC*

>Sevir.5G388200.1.p.svi.32668924/1-554

MAPAAVALLVVLIPAIFTATASAAVVEHTFNVGGMEIWQLCMNSVIYTVNQQLPGPTIEVSEGDTLVVHVVN

GAPYPMSMHWHGIFQLQSGWADGAHMITQCPIPPSGKFTYVFNVTGQEGTLWWHAHSSMLRATIYGALIIKP

RGGYPFPAPYAEIPILLGEWWNRNVDDVETDGLLTGLGPAMSDAFTINGSPGDQTPCGGAGIFQVEVEPGKT

YLLRIINAAVNAELFFRVAGHTFTVVAVDASYTNPHPTDVVVIAPGQTVDALMSASAAPGRYYMAARAFESK

TVANPPPFDNATATAVLRYRGVPDAAPAAMPALPPYTDVVTAARFYWSMTGLVRPGDPVVPTRVDHSMVVAF

GLEQAPCAPDQTKCQGFAVVASMNRYSFRFPEEVSLLEALFRGVPGVYSEDFPRSPPAVAAARRATSVRKVN

FNDVVEVVLQNEAYSSVLGAENHPIHLHGFNFFVLAQGLGRFDRGMKSTYNLVNPQVRNTVAVPAGGWAVIR

FTANNPGMWFMHCHLDAHLPLGLAMVFEVLNGPAPNLLPPPPADFPKCH*

>Sevir.5G388000.1.p.svi.32669055/1-578

MHFRIMPGSWLILVLAVSSAAVLAHAATVEHTFNVATLCWPRISQPGDVSITAVDGVPGPVIEAYEGDTVVV

HVINDSPHNVTIHWHGVIQRGTPWADGPEMVTQCPIRPGTRYTYRFNVSGQEGTLWWHAHASFLRATVHGAL

IIRPRAGAGAYPFPTPDGEAVVLLGEWWNDENAISSNVIADAYTINGKPGDLYAGETTANRSAKFEVTRNST

YLLRIINAALNTAFFFKVAGHTFTVVAADASYTTSYETDVIVIAPGQTVDALMAADASPGCYYMAISSYQSA

FPPPPGGFNGNVTTSLVEYAGAAAPGGGQQAPALPDTPEPTDTDTANRFYTGLTALVRPGMPRVPLTVDTRM

FVTVGLGLRYPPCEPTQTTPCQPIPVATMNNQSFVLPSAMSMLDARYRNTPDGVYTRDFPDRPPVEFDYTNR

TEMVVGGSAAALLFPGMPATKVRKLEYNATVEMVLQNTALVGRESHPMHLHGFDFFVLAQGFGNYDDATGSQ

QFNLRNPQERNTIAVPTGGWAVIRFVADNPGMWFMHCHIDSHLSIGLGMVFEVEDGPTPDTKLPPPPADLPQ

C*

>Sevir.5G217600.1.p.svi.32670237/1-629

MKNRSLPAASAIVAAAIVFFLSVMALPVAVAVVEHTFVVSQVNMTHLCKETLVTVVNGQLPGPPIEVTEGDS

VAVHIVNESPYNITIHWHGVKQRLNCWSDGVPMITQCPIKPNQNFTYRFNVTGQEGTLWWHAHVPCLRASLH

GALIIRPRNGASSYPFPKPHKEIPIIIGEWWEKDLSVVGRNMRDGSLDDDPSASTINGKLGDLFNCSGAPED

GYVLEVEPGKTYMLRVINAALFYEYYLKIAGHKFTVVAADANYVNPYTTDVIAVSPGETVDAILIADAPPGS

YYMVAQPVKAPLPDTRTPVYVTRGVVQYNSNRSYGNGTTEQQSSSHGANEGIPFGDAPVVPEMPGMHNTVVS

FDFHGNLTSLRHPRRPMVPLRVDEHLFIALGLGMTPCRRGQSCNRRKGDERIIAATMNNVSFHLSSIRTPIL

EAHYYHTGGRKDDGTLLELPDRPPRTFNFTDPALIPEGDKEALLEPTSKETVARWFRHGATVEVVFQSTALL

QGSSNPMHLHGHDMYLLAQGHGNYDAAKDVARYNLVNPPMKNTVHVPNLGWAAVRFVADNPGVWFMHCHYEF

HLSMGMAAMFIVEDGPTVGRSLPPPPMDFATCGNYESYLQTKKSEVSHVDGV*

>Sevir.5G245600.1.p.svi.32670316/1-567

MTMASSRHHRLPLLLSAALVLALSVLPAAQADVQRYQFDIVMSNVSRLCHAKSMATVNGSYPGPTIYAREGD

RVVVAVTNRVAHNVTIHWHGLKQRRNGWADGPAYVTQCPIQPGGTYAYDFNVTGQRGTLWWHAHIAWLRATV

HGAIVVLPARGVPYPFPKPDAEVEIILGEWWHADVEAVEKQGRALGMAPNTSDAHTINGKPGPLFPCSDKHT

YALQVQWGKTYLLRIINAAVNDELFFSIAGHTMTVVEIDATYTKPLLASTIQLSPGQTTNVLVRADQRPGRY

FMAAKPFNDAAVPADNKTATAILQYAGVPASVLPAPPRLMPETNGTGFVAAFHDRLRSLNSARYPAAVPLAV

DRRLLYAIGLNIDPCASCPKGSRLAASLNNITFVMPRVALLQAHYGGLKGVFAADFPDRPPARFNYTGAPLT

AGLGTSLGTRLSRVAYNASVELVLQDTNLLSVESHPFHLHGYNFFVVGRGVGNFDPAKDPAKYNLVDPPERN

TVGVPAGGWTAIRFRADNPGVWFLHCHLEVHTSWGLKMAFLVEDGDGPDESVLPPPKDLPKC*

>Sevir.5G388100.1.p.svi.32671090/1-652

MVRLLLALALLLVGPVADAATAKYTFTVGSTQISQLCSTTSIIAVNGQLPGPSIEVNEGDAVEVKVINNSPY

NVTIHWHGVFQLMTPWADGPSMVSQCPIQPSGSYTYRFSVPGQEGTLWWHAHSSFLRATVYGAFIVRPRAGN

SYPFPAPDQEVPIVLGEWWNRNVVDVENDAILSGQLPTQSDAFTVNGKTGLLYQCANETFTAVVEPNTRVLL

RVINAGLNSHLFFKVAGHNFTVVAVDGSYTSNLNTDTLVIAPGQTVDALVTTSAAPGSYYMAVLAHDTMSPL

AFAASDTTTATAILQYNGTSTNPPAMPVMPSSSDSATANAFYFGLRGLGTPAVPSPVDVSMTIELGLGQLPC

DPSQTKCNGTAAAAAMNGVSFRLPAPETSLLGAHLNGLTGVFTADFPDGPPPSGTAMAVGTKVKKLAYNSVV

EIVLQNPSAVPTENHPIHLHGFNFFVLAQGVGTFTPGSASYNLVDPVARNTIAVPGGGWAVIRFVANNPGMW

FFHCHLDPHVPMGLGMVFHVDSGTTAGATLPPPPADWVGVCDAQNYATAAAAAAAAATPAPVPAPAPAPTLA

PGSAPAAATSPRAAAGSPVKPSSPVDHKPSPNLPQRRGDGRPSATSAAAEPRATGHLTCLLSTIILFFVLHE

HKA*

>Sevir.5G383000.1.p.svi.32673265/1-580

MATSYLLLPYCVLVAVLVLLFSVDVAEGAIREYQFDVQMTSVTRLCSSKSIVTVNGLFPGPTVFAREGDLVV

VRVVNHVPYNMSIHWHGVRQLRSGWADGPAYITQCPIQSGQSYVYKFTITGQRGTLWWHAHISWLRATVYGP

IVILPKPGVPYPFPAPYKEVPVIFGEWWKADTEAVISQALQTGGGPNVSDAFTINGLPGPLYNCSAKDTFKL

KVQPGKTYMLRIINAALNDELFFSIAGHPLTIVDVDAVYIKPITVETLIITPGQTTNVLLTTKPSYPAANYY

MLAAPYSTARPGTFDNTTVAGILEYEDPNSPSPAAFNKNLPILKPTLPQINDTSFVANFTGKLRSLATAEYP

ADVPREVDRRFFFTVGLGTHPCAVNGTCQGPTNDTRFAASVNNVSFVLPTTALLQAHFTGRSNGVYSPNFPA

APLIPFNYTGTPPNNTNVSNGTKLVVLPYGTSVELVMQGTSILGNESHPLHLHGFNFFVVGQGFGNFDPVKD

PAQYNLVDPVERNTVGVPAGGWVAIRFRADNPGVWFMHCHLEVHVSWGLKMAWLVLDGDKPKEKLLPPPSDL

PKC*

>Sevir.5G371400.1.p.svi.32673586/1-568

MASPPSRMLILLSCLALSLLAGAEVHHHEFVVQETPVKRLCKTHNIITVNGQFPGPTLEVREGDTLVINVVN

RAQYDVTIHWHGIRQLRTGWADGPEFVTQCPIKPGGSYKYRFTIEGQEGTLWWHAHSSWLRATVYGALIIRP

RENKTYPFEKPSREVPVILGEWWNANPVDVIREAQRTGGAPNVSDAFTINGQPGDFLKCSEKETTAIPVKPG

ETALLRFINAALNHELFVTIAQHKMTVVAADASYTKPFTTSVLMIAPGQTTDVLVTMDQAPTRYYVAARAYV

SGQNVAFDNTTTTAVIEYDCGCASDFGPKIQPAFPALPAFNDTAAATAFAAGIKSPDRVKVHENVDEYLFFT

VGLGLFNCKPGELCAGPNNNTRFTASMNNVSFVFPKKDSLLHAHYYKVPGVFTTDFPAYPPVQFDYTAKNVS

QALWQPVPATKLYPLRFGSVVQLVLQDTSIVTPENHPIHIHGYDFFILAEGFGNFDPKKDVEKFNYVDPPQR

NTVAVPVNGWAVIQFVADNPGVWLMHCHLDVHINWGLAMAFLVEDGYGELQSLEPPPVDLPMC*

>Sevir.5G464400.1.p.svi.32674430/1-486

MATVNGSYPGPTIYAREGDRVVVAVTNRVARNVTIHWHGLKQRRNGWADGPAYVTQCPIQPGGTYAYDFNVT

GQRGTLWWHAHIAWLRATVHGAIVVLPARGVPYPFPKPDAEVEIILGEWWHADVEAVEKQGRALGMAPNTSD

AHTINGKPGPLFPCSEKHTYALQVQWGKTYLLRIINAAVNDELFFSIAGHTMTVVEIDATYTKPLSASAIQL

SPGQTTNVLVRADQRPGRYFMAAKPFNDAAVPADNKTATAILGTPALRSLNSARYPAALPLAVDRRLLYAIG

LNIDPCASCPKGSRLAASLNNITFVMPRVALLQAHYRGLKGVFAADFPDRPPARFNYTGAPLTAGLGTSLGT

RLSRVAYNASVELVLQDTNLLSVETHPFHLHGYNFFVVGRGVGNFDPTKDPAKYNLVDPPERNTVGVPAGEW

TAIRFRADNPGVWFLHCHLEVHTSWGLKMAFLVEDGDGPDESVLPPPKDLPEC*

>Sevir.3G222800.1.p.svi.32676594/1-578

MASSSAVPSSAVAVAAAVLLLAAVGAEAETRKYQFNVQMASVTRLCGTKSIVTVNGQYPGPTLFAREGDHME

VTVVNRSPYNVSLHWHGVRQLLSGWADGPAYITQCPIQPGGSYVYRYQIVGQRGTLWWHAHISWLRSTLYGP

IVILPPAGVPYPFPKPDEEVPLMFGEWWRNDTEAVIAQALQTGGGPNISDAYTINGLPGPLYNCSAQDTFRL

KVKPGKTYMLRLINAALNDELFFSVANHTLTVVDVDALYVKPFAVDTLVIAPGQTSNVLLAAKPAFPGARYY

MEARPYTNTQGTFDNTTVAGILEYEDPSSSSATTAAANLPIFAPTLPQINDTNFVANYTARLRSLATAAYPA

AVPQSIDRRFFFTVGLGTHPCAVNGTCQGPNGSRFAAAVNNVSFVLPTTALLQAHFAGRSNGVYTTDFPAVP

LMPFNYTGPPPNNTNVMNGTRVVALPFGTTVELVLQDTSILGAESHPLHLHGFNFYVVGQGFGNFDPVNDPA

KFNLVDPVERNTVGVPAGGWVAIRFRADNPGVWFMHCHLEVHMSWGLKMAWLVQDGSLPNQKLPPPPSDLPQ

C*

>Sevir.3G223200.1.p.svi.32677580/1-578

MAGGRRLRCLSPACLFLAVAVALLAMPGLAAARTRRYTFNVTMATVTRLCATRSIPTVNGRFPGPKIVVREG

DRLIVQVHNNINNNVTFHWHGVRQVRSGWADGPSFITQCPIRPGQSYAYNFRIIGQRGTLWWHAHFSWLRAT

LYGPLVILPPLGVPYPFPKPDREVPLMLGEWFNADPEAVIRQALQTGGGPNVSDAYTFNGLPGPTYNACSAG

DTFRLRVRPGRTYMLRLVNAALNDELFFAVANHTLTVVGADASYVKPFTAATLVISPGQTMDVLLTAAAATP

PSPAYAIAVAPYTNTVGTFDNTTAVAALEYAPQAALRGLPLPALPLYNDTGAVANFSANFRSLASAQYPARV

PRTVDRKFFFAVGLGADPCPSRVNGTCQGPNGTRFAASMNNVSFTMPKTSLLQAHYQRRYSGVLTANFPAMP

PMTFNYTGTPPNNTFVTHGTRVVPLRYNTTVEVVLQDTSILGAESHPLHLHGYDFYVVGTGFGNYDANNDTA

KYNLVDPVQRNTISVPTAGWVAIRFVADNPGVWIMHCHLDVHLSWGLAMAWLVNDGPLPNQKLPPPPSDIPK

C*

>Sevir.3G086900.1.p.svi.32678244/1-614

MHISPNHTRTAVFFSERSTSRTLLGICMARSWSLVLPFGLVIALLFACVAQAAVVEYTFNVGNLSISQLCQP

PLIITAVNGQLPGPTIEAREGDTVVVRLVNQSPYNMTIHWHGVFQRGTPWSDGPAMVTQCPVKPGGAYTYRF

NVTGQEGTLWWHAHTESYLRATVYGAIVIRPRAGAAAYPFPTPDGEETVILGEWWNANVYDLRRNAFLRGNP

PINSNAYTINGKPGDFYNCSDANQTYRFQVRSNGRYLLRIINAALNTNMFFKVAGHRFTVVAADAAYTAPYD

TDVVVIAPGQTVDALMVAGAAPGQYYMAASPYVSATPPNRGPPFSMSNATAVVEYAGSTTTAPPQPPTMPPY

TDTATAFRFFTSLKAPVPSGAPAVPLSVDTRMFVTVGLGISDCQPAQLLCNPTGTRTLPVLAASMNNASFVL

PPAGSVSMLQAHYDADGSAPAPSVYTRDFPDRPPVIFNYTADASDIPTLQYTTKSTKVKTLRYNETVEMVLQ

STRLLANESHPMHLHGVNFYVLAQGVGNYDEAAAAPRFNLENPQERNTVAVPPGGWAVIRFQANNPGVWFMH

CHIEDHLDVGLAMAFEVQDGPTPETSVPPPPLDLPQC*

>Sevir.3G329700.1.p.svi.32679082/1-576

MHRSWSLVLPLGLIALLFASVAEAAAVEHTFNVGNLSISQLCQPARIITAVNGQLPGPTIEACEGDTVVVHL

VNESPYNMTIHWHGIFQRGTPWADGPAMVTQCPVKPGATYTYRFNATDQEGTLWWHAHISLLRATVYGALVL

RPRGGAGAYPFPKPHGEETVLLGEWWNANVHDLDDMAFLTGNPPRNADAYTINGKSGDFYNCSNANETYRFQ

VRRNETYLLRIINAALNTPMFFKVANHSFTVVGADAAYTTPYETDVVVVAPGQTVDALMVAGAAVGRYYMAA

SPYDSAIPVGPPFSMSTATAIVEYAGSAAEAPPQLPSRPEYNDTDTAFRFLSNLTALVLPGKPTVPLSVDTR

MFVTVGLGNSDCLPKQLLCNTTGTRMPIFSASMNNASFVLPESVSMLQAHYANASAGVYTRDFPDRPPVIFD

YTADASDNATLKYTPKSTKVKTLRYNETVEMVLQNTRLIAKESHPMHLHGFNFFVLAQGFGNYNEAAAKPQF

NLVNPQERNTVAVPTGGWAVIRFLANNPGMWFMHCHFDAHLDLGLAMVFEVQDGPTAETSVPPPPLDLPQC*

>Bradi2g23350.1.p.bdi.32771988/1-578

MAVSSSLLCLLFAALTATLVGAQAVTRKYQFDVQMTSVTRLCSTKSIVTVNGQYPGPTLFAREGDHVEVNVV

NNSPYNMTIHWHGVRQLRSGWYDGPAYITQCPIQPGQSYVYRFTITGQRGTLWWHAHVSWLRATVHGPIVIL

PPLGVPYPFSPVPYKEVPLMFGEWWKNDTEAVIAQALQTGGGPNISDAFTMNGLPGPLYNVCSSAKGETFKL

KVEPGKTYMLRLINAALNDELFFAVANHTLTVVDVDALYVKPFAVESLVIAPGQTSNVLLTANNNPNARYYM

LARPYTNTQGTFDNTTVAGILEYEDTSNGKAPSTSSLPILRPTLPEINDTSVVSNYTAKLRSLASAEYPASV

PQQVDREFFFTVGLGTHPCQSLINGTCQGPNNTRFAASINNISFVLPTTALLQSHYTGMSNGVYASNFPFYP

LRPWNYTGTPPNNTNVMNGTKALVLPFGVAVELVMQDTSILGAESHPLHLHGFNFFVVGQGFGNYNPSSDPA

KFNLVDPVERNTVGVPAGGWVAIRFRADNPGVWFMHCHLEVHMSWGLKMAWVVLDGARPDQKLPPPPADLPK

C*

>Bradi2g54680.1.p.bdi.32772136/1-578

MAMAISSGLPACSVVMATLMVLIIQAQGITRHYDFDVQMAKVTRLCGSKSIVTVNGQFPGPELVAREGDRVH

VRVTNHVSHNMSLHWHGIRQMQTGWADGPAYITQCPIQMGQTYVYKFTITGQRGTLWWHAHISWHRATVYGA

IVILPKLGVPYPFAAPHKEVPVIFGEWWAADTEVVMSQALKVGGAPNISDAFTINGLPGPLYNCSAQDTFKL

KVTPGKTYLLRLINAALNDELFFSVANHTLTVVEVDAVYVKPFTVKTIVISPGQTTNVLLTAKPVNPKANFY

MSAAPYSVIRPGTFDNTTVAGILEYHEDPSSSSSFDKNLPLFKPMLPRFNDTKFVTNFTTKLRSLATTKYPA

AVPQTVDKRFFFTIGLGTLPCPKNMTCQGPNGTQFAAAVNNVSLVLPTKALLQSHFTGLTTGVYASDFPAMP

LSPFNYTGTPPNNTNVATGTKLLALPFNTSVELVMQDTSVLGIESHPLHLHGFNYFVVGQGFGNYDSAKDPA

KFNLVDPVERNTVGVPAGGWVAIRFLADNPGVWFMHCHLEVHTTWGLRMAWLVHDGSKPNQKLLPPPSDMPK

C*

>Bradi2g23370.1.p.bdi.32772671/1-598

MPLTTLKCSHIQEDLHKMGGAHHGLLRCASPACLHLAFFLLLAVEPGLTAGLTRRYTFNVTMATVTRLCLTK

SIPTVNGQFPGPKISVREGDRLVVNVHNNINNNVTFHWHGVRQLRSGWADGPAYITQCPIRPGQSYVYSFRI

VGQRGTLWWHAHFSWLRATLHGPMVILPPLGVPYPFPKPYREVPLMLGEWFNADPEAVIKQALQTGGGPNVS

DAYTFNGFPGPTYNCSAKSTYKLKVKPGRTYMLRLINAALNDELFFAVANHTLTVVQADASYVKPFTANTLV

ISPGQTMDVLLTAAASTSSSAFAIAVAPYTNTVGTFDNTTAIAVLEYSPQRPSMLRNLPAPSLPLYNDTNAV

TNFSSKFRSLDNARYPAQVPMTVDRHFFFTVGLGADPCQSPINGTCQGPNNTRFAASINNVSFIMPKTSLLQ

AHYQRMYKGVLMANFPTAPVRKFNYTGTPPNNTFVTHGTRVVPLAFNTTVEVVLQDTSIQGAESHPLHLHGY

DFYVVGTGFGNYDANNDTAKYNLVDPVKRNTISVPTAGWVAIRFIADNPGVWIMHCHLDVHLSWGLSMAWLV

NDGPLPNQKLPPPPADIPKCS*

>Bradi2g54690.1.p.bdi.32772704/1-579

MAATFRSSSLLVVAALMVLSINLAEGDTVEYQFDVETMNVTRLCSSKSIVAVNGQFPGPTVLAREGDLVVVR

VVNKAQYNMSIHWHGVRQLRSGWADGPAYITQCPIQPGQSYVYKFTISGQQGTLWWHAHISWLRATVYGPIV

ILPKLGVPYPFPAPFKEVPLMFGEWWKADTEAVISQALQTGGGPNVSDAFTINGLPGPLYNCSAKDTFKLKV

KPGKMYMLRIINSALNDELFFSIAGHLLTVVDVDAVYVKPVTVETLLITPGQTTNVLLTTKPSYPGANYYML

ASPYSTAASGTFDNTTVAGILEYEHDEYPGSSASFNKNLPLFKPTMPQINDTSFVSNFTAKLRSLANEDYPA

DVPREVDRRFFFTVGLGTHPCAGANGTCQGPNGSRFAAAVNNVSFVLPTTALLQSHFTGMSNGVYESNFPAM

PSSPFNYTGTPPNNTNVSNGTKLVVLSYGESVELVMQGTSILGAESHPFHLHGFNFFVVGQGFGNFDPMSDP

AKYNLVDPVERNTVGVPAAGWVALRFRADNPGVWFMHCHLEVHVSWGLKMAWLVQDGSLSNQKLLPPPSDLP

KC*

>Bradi2g55050.1.p.bdi.32778355/1-552

MAQLLWLASVLLLAASSVADAATANYTFTVASMRVNRLCNSTDIIAVNGQLPGPTIEVNDGDEVVVNVTNGS

PYNLTIHWHGMLQLLTPWADGPSMVTQCPIQPNSSYAYRFNVTGQEGTLWWHAHSSFLRATVYGALIVKPRN

GSAYPFPTPDQEVPLVLGEWWSQNVVDVEKDALMSGQLPSRSDAFTVNGLTGQLYPCANETFTVVVEPNTTV

LLRVINAALNTHLFFKLAGHNFTVVAVDACYTANHTTDTLVLAPGNTVDALIFTGPKPAGSYYMAVQPHDTI

SPATMATSDDDSTATAILRYNGTSPTATPAMPAMPTSSDTSTANAFYFGLRGVKAFTAVPTKVDVNMTIELG

LGQLPCDSAQTSCNGTAFAAAMNGVSFRLPTRVSLLEAQFKGKPAGVYTADFPDGPPGSGMAMVEGTKVRSL

PYNSTVEIVLQNPTAVPAENHPIHLHGFNFFVLAQGLGTFTPGNASAYNLVDPVSRNTLAVPTGGWAVIRFV

ANNPGMWFFHCHLDAHVPMGLGMVFAVQNGTAPGSILPPPPADLPMC*

>Bradi2g55050.2.p.bdi.32778356/1-552

MAQLLWLASVLLLAASSVADAATANYTFTVASMRVNRLCNSTDIIAVNGQLPGPTIEVNDGDEVVVNVTNGS

PYNLTIHWHGMLQLLTPWADGPSMVTQCPIQPNSSYAYRFNVTGQEGTLWWHAHSSFLRATVYGALIVKPRN

GSAYPFPTPDQEVPLVLGEWWSQNVVDVEKDALMSGQLPSRSDAFTVNGLTGQLYPCANETFTVVVEPNTTV

LLRVINAALNTHLFFKLAGHNFTVVAVDACYTANHTTDTLVLAPGNTVDALIFTGPKPAGSYYMAVQPHDTI

SPATMATSDDDSTATAILRYNGTSPTATPAMPAMPTSSDTSTANAFYFGLRGVKAFTAVPTKVDVNMTIELG

LGQLPCDSAQTSCNGTAFAAAMNGVSFRLPTRVSLLEAQFKGKPAGVYTADFPDGPPGSGMAMVEGTKVRSL

PYNSTVEIVLQNPTAVPAENHPIHLHGFNFFVLAQGLGTFTPGNASAYNLVDPVSRNTLAVPTGGWAVIRFV

ANNPGMWFFHCHLDAHVPMGLGMVFAVQNGTAPGSILPPPPADLPMC*

>Bradi2g53800.1.p.bdi.32779127/1-567

MASSRLLFLLSCSCLALALLASAEVHHHEFIVQETPVKRLCKEHNIITVNGQFPGPTLEVREGDTLVVNVVN

QAQYNVTIHWHGIRQFRTGWADGPEFVTQCPIKPGGSYKYKFTIEGQEGTLWWHAHSSWLRATVYGALIIRP

REDKAYPFEKPSREVPLMLGEWWDANPIDVIREAQRTGGGPNVSDAFTVNGQPGDLYNCSREETTAISVKPG

ETALLRFINSALNHELFVSIANHKMTVVGADASYTEPFVTSVLMIAPGQTTDVLVTMDQAPTRYYIAARGYV

TTQGVAFDNTTTTAILEYDCGCSTDFGPAIRPAFPTLPAFNDTSAATAFAAGIKSPRKVEIPSPVDENLFFT

VGLGLFNCKPGQQCGAPNNTRFTASMNNISFVFPKATSLLHAHYYDIPDVFTTDFPAYPSVQFDYTAQNVSR

SLWQPIPATKLYKLRYNSVVQIVLQDTSIVTPENHPIHLHGYDFYILAEGFGNFDAKKDAEKFNLENPPQRN

TVAVPVNGWAVIRFRADNPGVWLMHCHLDVHITWGLAMAFLVEDGYGKLQTLEAPPVDLPMC*

>Bradi2g55060.1.p.bdi.32779171/1-555

MERAAMIVPLVLALCTAMASAAIVEHTFNVGGMNISQLCTDTVIYTANEQLPGPTIEATEGDTVVVHVVNDS

PYPLSIHWHGIFQLLSGWADGAHMITDCSVQPSGNFTYQFNVTAQEGTLWWHAHSSLLRATIYGALIIKPRN

GTDGYPFPAPYGEIPILLGEWWNKNVNDVEIDAHLTGLGQDISDALTLNGLPGDQTPCTGAGIYGVEVEYNK

TYLLRIINAAVNVELFFKVAGHNFTVVAIDASYTDPYATDTIVITPGQTVDALMTTSAPPGRYYMAANVFDS

KTVPFRFNTGTATGIVKYEDAPDDDATATMPTMPSHEDVVTAGNFYWSLTGLVRPSDPPVPKTVDHDMVVEF

GVDQAPCAVEQTKCQGFALVAFMNRNSFQFPRNASLLKALYDGVQGRVYSEDFPSSPPPLPGIRRATSVKRL

NYGDVVEVVLQSRVYSSVLGAENHPIHLHGFNFFVLAQGLGRFDPRANSTYNLVNPQVRNTVAVPAGGWAVI

RFTANNPGMWFMHCHLDAHLPLGLAMVFEVLDGPAPNLLPPPPVDYPKCY*

>Bradi2g54740.1.p.bdi.32779499/1-577

MGALRGLRRHAAASACPFLAFAVLLALPGLAAGITRHYTFDVQMTNVTRLCATKSIPTVNGQFPGPRLVARE

GDRLVVKVHNHINYNVSFHWHGIRQLRNGWADGPAYITQCPIQGGQSYVYDFTITGQRGTLWWHAHFSWLRV

HLYGPLVILPKRAEGYPFPLPYKEFPIMFGEWFKADSEAVINQALQTGAGPNVSDAYTFNGLSGPTYNCSSK

DTYKLKVQPGRTYMLRLINSALNNELFFGIANHTLTVVEADANYVKPFTSKTVVISPGQTMNVLLTTSSNPA

SRAFAMSIAPYTNTQGTFDNTTATAVLEYASTRPSSTQNLAMPALPRYNDTNAVANFSSNFRSLASAQYPAR

VPQAVDRHVLFTVGLGTDPCPSNQTCQGPNGTKFAASINNNSFVRPKTALLEAHYQSRYAGVLMANFPTTPP

HPFNYTGTPPNKTFVAHGTRVVPLSFNTTVELVMQGTSIQGAESHPLHMHGFNFFVVGQGFGNYDPVNDPAK

YNLIDPVERNTVSVPTAGWVAVRFLADNPGVWLMHCHFDVHLSWGLSMAWLVNDGPLPNQKMLPPPSDLPKC

*

>Bradi4g39330.1.p.bdi.32783985/1-584

MARSSPLLLLLFALALVAPVAHAAVVEHTFNVGNLSISQLCRPEMNITAVNGQLPGPTIHAKEGDTVIVHLL

NDSPYNMTIHWHGIFQRGSQWADGPVMVTQCPVRPAANYTYSFNVTGQEGTLWWHAHISFLRATVYGALVIL

PRGGAGAYPFSPKPDKEMVVMLGEWWNDNVHELQETAFLTGNPAPAADAYTINGKPGDLYNCSAPNHTRRFE

VRRNETYLVRIINAALNTPLFFKVANHSFTVVGADACYTTPYRTDVVVVAPGQTVDALMATPDAPPVAGQRR

YYMAASPYDSAIPGPGPPFSPTIATAVLEYVGGGDNTTTDIQMPDMPPFTDTNTAHRFLSNLTALVLPGKPT

VPLGAMGTRMFVTIGMGQADCQPDQTMCDPTSTKFASSMNNASFVLPRGTLSMLEAHRSNATAGVYTRDFPD

RPPLIFDYTADASDTAQLQYTTKSTKVRTLRFNETVEMVLQNTRLIAKESHPMHLHGFNFFVLAQGFGNYNE

TVAVPQFNFVNPQERNTLAVPTGGWAVIRFIADNPGMWYMHCHFDAHLDFGLGMVFEVLDGPTPETSVPPPP

KDLPRCR*

>Bradi4g11850.1.p.bdi.32786337/1-601

MALSPAAAVAVFFFLAMLVGGDAAVVEHTFVVSQVRMNRACKDTLVTVVNGQLPGPAIEVTEGDSVVVHVVN

KSPHGLTIHWHGVKQRLNCWADGVGMITQCPIQPGKNFTYRFNVAGQEGTLWWHAHVASLRATVHGALIIRP

RSGGDAGTLYPFPRPHKEVPIFIGEWWDVDLVELDMRMADGFFDDYPVNATINGMLGDLYDCAGASEDIYVM

QVEPGKTYMLRIVNVALLSRYYLKIAGHRFTVVAADANYVKPYNTDVIAIAPGESMDALVVADAPHGKYYMV

ALANQPPLPDQQIPVFSSRGVLQYSYNPIKGEDPTVINSLMVPEMPDQHDMMTSFYFHGNLTGATPHPPLPI

NVDDRLFISLALGSICRGSHVPLPSSPCRGYDVNESISVATMNNVSFQLPTKVSLLEAHYRGNMSIAGKLHE

LPDKPPTEYNYTDKTMIPWGSKEAALESTTKAMSVRMFRFNTTVEIVFQSTAILNSDANPMHLHGHDFFVLA

QGLGNYDAERDAGSYNLVDPPVRNTVLVPPVGWAAIRFVTDNPGVWFLHCHYGFHTSMGMAVAFEVENGQSS

DMTLPPPPIDLPRCEHHGNSVAYQ*

>Bradi4g44810.1.p.bdi.32787154/1-570

MASSACSSLFISIIAALLLCCSQLTAAKEQQHEFVIQETAVKRLCNGGMSIVTANGQFPGPTVEVSEGDSLV

VNVVNNATYNVTIHWHGVRQMRTGWSDGPEFVTQCPIRPGQSYTYRFTVTGQEGTLWWHAHSSWLRATVYGA

LLIRPRDGVPYPFDFAAETVPILLGEWWDMNPIDVIRAATRTGAAPNVSDALTVNGQPGDLYSCSSSQDTTV

FPVKSGETNLLRFINAALNTELFVSLAGHAMTVVGADASYTKPYNTSVLVLGPGQTTDVLVTFDQPPGRYYL

AARAYASAQGVPFDNTTTTAIFDYGAGDGTTSPAMPTLPAYNDTATVTAFTTSLRNLHSIGLPSVVDEDLFF

TVGVGLFNCSKGQSCGGPNNTRFAASINNVSFVLPSTVSILQAHYDGGANAGVFTTDFPANPPVQFDYTAQN

VSRGLWQPVPGTKLYNLKYGAVVQVVLQGTNIFAGENHPIHIHGYDFYILAEGFGNFDAATDTAKFNLDDPP

MRNTVGVPVNGWAVIRFVADNPGVWLMHCHLDVHITWGLAMAFLVKDGVGELQSLGAPPPDLPIC*

>Bradi4g11840.2.p.bdi.32791581/1-607

MAAMALSATSVAVFFFLAVLSGGDAAVVEHTFVVSQVKMNRACRGDTLVTVVNGQLPGPAIEVTEGDSVVVH

LVNKSPYGLTIHWHGVKQRLNCWADGVDMVTQCPIQPGRNFTYRFNVAGQEGTLWWHAHVASFRATVHGALI

IRPRSGVTSYPFPEPHKEIPIFIGEWWEVDLVKLDTTLGDGVDYNPVNTTINGKLGDLYNCSVHLCDSGCEG

ALEDNFIIEVEQGKTYLLRIVNAALFSEYYLKIAGHRFTVVAADANYVKPYTTDIIAIAPGESVDAIVLADA

PPGKYYMVALGNQQPPPDIQIPPLSSRVLVKYNYNPSKEQALTLGASVMAPEMPDQHNTIASFYFHGNMTGS

FAHLPVPVHVDEHLFISLALGVICRGGHVPSPSLPCSKLNGSILAATMNNISFEFPSNVSLLEAHYRGNMSN

AGMLHKLPDKPPIVYNFTDRAMISEGSEEAALESTLIATTVRRFRFNTTVEIVFQSTTVLQSDSNPMHLHGH

DFFVLAQGLGNYNAERDVGRYNLVDPPVRNTVLVPSSGWAAIRFVTDNPGVWFLHCHYGFHMSIGMAVVFEV

DNGQTLNTTLAPPPADLPICEQHDSSVAYE*

>Bradi4g11840.1.p.bdi.32791582/1-598

MAAMALSATSVAVFFFLAVLSGGDAAVVEHTFVVSQVKMNRACRGDTLVTVVNGQLPGPAIEVTEGDSVVVH

LVNKSPYGLTIHWHGVKQRLNCWADGVDMVTQCPIQPGRNFTYRFNVAGQEGTLWWHAHVASFRATVHGALI

IRPRSGVTSYPFPEPHKEIPIFIGEWWEVDLVKLDTTLGDGVDYNPVNTTINGKLGDLYNCSGALEDNFIIE

VEQGKTYLLRIVNAALFSEYYLKIAGHRFTVVAADANYVKPYTTDIIAIAPGESVDAIVLADAPPGKYYMVA

LGNQQPPPDIQIPPLSSRVLVKYNYNPSKEQALTLGASVMAPEMPDQHNTIASFYFHGNMTGSFAHLPVPVH

VDEHLFISLALGVICRGGHVPSPSLPCSKLNGSILAATMNNISFEFPSNVSLLEAHYRGNMSNAGMLHKLPD

KPPIVYNFTDRAMISEGSEEAALESTLIATTVRRFRFNTTVEIVFQSTTVLQSDSNPMHLHGHDFFVLAQGL

GNYNAERDVGRYNLVDPPVRNTVLVPSSGWAAIRFVTDNPGVWFLHCHYGFHMSIGMAVVFEVDNGQTLNTT

LAPPPADLPICEQHDSSVAYE*

>Bradi1g24910.2.p.bdi.32798700/1-568

MANVTRLCATKSIMTVNGEFPGPALVAREGDRVLVRVTNQVSHNMTLHWHGIRQLRSGWADGPAYVAQCPIQ

IGQSYVYNFNITGQRGTLWWHAHISWIRATVYGAIVILPELGIPYPLAAPHEEVPILFGEWWKANTEAVVKQ

ALQTGGAPNISDAFTINGLPGPLYNCSAKGMNELVLKVEAGKTYLLRLINAALNDELFFVIANHKLTVVEVD

AVCVKPFTVNTLVISPGQTTNVLLTAKPFNPKANFYMSAVPYSTIRPGTFDNTTVTGILEYHNPNSGSASSS

FDKDLPLFKPTMPRFNDTGPVTNFTTKLRSLATATYPVIVPRSVDKRFLFTIGPGTLPCPVNMTCQGPTNVT

RFAAAMNNVSLVFPSTALLQSHYTGMGMSKGVYASNFPTAPLTPFSYTGTPPNNINVAKGTRLLVLPFNTSV

ELVMQETSILGVESHPLHLHGFNFFVVGQGFGNYDAVNDLAKFNLVDPVERNTVGVPASGWVAIRFLADNPG

VWFMHCHLEVHTTWGLRMAWLVLDGSLANQKSQEILVCLVRSRLAFFRFSVIFVSSSFICLPS*

>Bradi1g66720.1.p.bdi.32799595/1-573

MGAKCLSLLVFLGTSLLLPQLLLAAMTRYYTFNVTMKKVTRLCNTRAIPTVNGKFPGPKIVTREGDRVVVKV

VNNVKHNVTIHWHGVRQLRTGWSDGPAYITQCPIQTGQSYVYNFTVTGQRGTLFWHAHVSWMRATLYGPIVI

LPKLGVPYPFPKPFKDVPIMFGEWFNVDPEAIIAQALQTGGGPNVSDAYTINGLPGPLYNCSSRDTFKLKVQ

PGKWYLLRLINAALNDELFFSIANHTLTIVDVDASYVKPFDTDVVLVTPGQTTNVLLHAKPDEGCQPATHLM

LARPYATSRPGTYDNTTVAAVLEYSPSGQIKSRPLFRPTLPVFNDTSFAANYSAKHRSLASSEYPANVPRRI

DRPFFFAVGLGTTPCPTHQGCNGPTNDTKFSASMNNVSFNMPTTALLKAHYDGNTAGVYTADFPAMPTQPFN

YTGTPPNNTNVSNGTKVAVLPYNASVEVVLQDTSIQGAESHPLHLHGFDFFVVGQGVGNYNASMHPAGFNLL

DPVQRNTVGVPAGGWVAIRFYADNPGVWFMHCHLEVHTSWGLKMAWVVNDGPLPDQKLMPPPSDLPKC*

>Bradi1g66720.2.p.bdi.32799596/1-418

MVKYATISCVGEWFNVDPEAIIAQALQTGGGPNVSDAYTINGLPGPLYNCSSRDTFKLKVQPGKWYLLRLIN

AALNDELFFSIANHTLTIVDVDASYVKPFDTDVVLVTPGQTTNVLLHAKPDEGCQPATHLMLARPYATSRPG

TYDNTTVAAVLEYSPSGQIKSRPLFRPTLPVFNDTSFAANYSAKHRSLASSEYPANVPRRIDRPFFFAVGLG

TTPCPTHQGCNGPTNDTKFSASMNNVSFNMPTTALLKAHYDGNTAGVYTADFPAMPTQPFNYTGTPPNNTNV

SNGTKVAVLPYNASVEVVLQDTSIQGAESHPLHLHGFDFFVVGQGVGNYNASMHPAGFNLLDPVQRNTVGVP

AGGWVAIRFYADNPGVWFMHCHLEVHTSWGLKMAWVVNDGPLPDQKLMPPPSDLPKC*

>Bradi1g74320.1.p.bdi.32803741/1-562

MPRHLSQLLLVVMVNCVLLQALSVHAITRHYKFNVVMRKMSRLCSTKTILTVNGKFPGPTLYAREGDNVLVK

VVNHVPHNVTIHWHGVRQIRTGWYDGPAYITQCPIQPGSSFLYNFTITGQRGTLLWHAHINWLRATVHGAIV

ILPKLGVPYPFPAPHKEAVVVLGEWWKADIETIINRAMQLGVGPNISDSHTINGHPGPMSDCASSQDGFKLN

VESGKTYMLRIINAALNDDLFFKIAGHKLTVVEVDAVYTKPYKTDILLITPGQTTNVLLAADQSAGRYLLSI

SPFMDAPVQVDNTTGTAILHYTNTVSAAARLTLFKPPPQNATLIASKFADSLRSLNSKEYPANVPRTVDHSL

FFTIGVGVNPCPNCINGTRVVGTINNLTFVMPSTPILQAYYYNIPGVFTEDFPATPPHKFNYTGSGPKNLRT

MNGTRVYRLPYNASVQVILQDTGIISTESHPIHLHGFNFFVVGRGIGNYSPKTSPSTFNLIDPIERNTIGVP

TGGWTAIRFRADNPGVWFMHCHFEVHTSWGLKMVFVVENGKRPSETLIPPPKDLLQC*

>Bradi1g24880.1.p.bdi.32804891/1-582

MAASPGLPAPWSLFMATLVLLIVQAQGITRHFDFNVQMANVTRLCATKSIVTVNGEFPGPALVAREGDRVLV

RVTNQVSHNMTLHWHGIRQLRSGWADGPAYVAQCPIQTGQSYVYNFTITGQRGTLWWHAHISWLRATVYGAI

VILPEFGVPYPFAAPHEEVPILFGEWWKADTEAVVKQALQTGGAPNISDAFTINGLPGPLYNCSAKDTFKLK

VQPGKTYLLRLINAALNDELFFSIAKHRLTVVEVDAVYVKPFTVDTLVISPGQTTNVLLTAKLFYPKANFFM

SAVPYSNIRPGTFDNTTVAGILEYHNPSSGSVSSSFNKDLPLFRPKLPRFNDTGLVTKFTAKLRSLATASYP

AAVPQSVDKRFFFTIGLGTLPCPTNTTCQGPTNITRFAAAVNNVSLVLPSTALLQSHYTGMGMSKGVYASNF

PTAPLSTFNYTGTPPNNTNVAQGTRLLVLPFNASVELVMQDTSILGIESHPLHLHGFNFFVVGQGFGNYDVV

NDPAQFNLVDPVERNTVAVPAGGWVAIRFIADNPGVWFMHCHLEVHTTWGLRMAWLVRDGSLPNQKLLPPPS

DLPKC*

>Bradi1g65100.1.p.bdi.32805128/1-600

MESASGVMRFCCCVSSLLLLCFLLPCALAEERFYEFVVQETVVKRLCKTQKIITVNGQFPGPTIEVHDGDTL

AIRAVNMAQYNVTLHWHGLRQLRNGWADGPEFVTQCPIRPGSSYTYRYTIQGQEGTLWWHAHSSWLRATVHG

ALIIHPKRGLPYPFPKPNKEFPVLLAEWWRKDPIAVIRQSMVTGAPPNISDTILINGQPGDFLECSSQETSI

IPVVAGETNLLRIINAAMNSELFVSLAGHKMTVVAADAVYTRPFETTVVLLGPGQTTDVLVTADAAPARYYL

AARVYASAQGVPFDNTTATAIFQYKNAAGCPTTTNPALNGPVGRPSQRSSAHPGRAGPAPLMPALPAFNDTN

TATAFSKSIRSPRPVKVPGPVTQEVFTTVGFGLFNCRPGPFCQGPNNTRFAASMNNVSFQLPNTVSLLQAHY

HHVPGVFTEDFPAVPPVIFDFTSQNVPRSLWQPVKGTRLYRVKYGAVVQMVFQDTGIFAAEEHPMHIHGYHF

YVLATGFGNYDARRDAAKFNMVDPPSRNTIGVPVGGWAVVRFVADNPGVWLVHCHIDAHLTGGLGMALLVED

GKAELQATVPPPLDLPICGVDGL*

>Bradi3g22317.1.p.bdi.32812870/1-600

MMTLPMAAATAFFFLALLVAVSTVLAAIVEHTFVVRQMHMQHLCKDTLVTVVNGQFPGPAVEATEGDTVVVH

VINQSPYGITIHWHGVKQRLTCWADGAGMITQCPIQPNTAFTYRFTVAGQEGTLWWHAHVASLRATLHGILI

IRPKSGSYPFQKPHMDVPIIIGEWWQKDLTEVEKGYLNSNDNDPAAAAINGKLGDLYNCSGVVENSYVLEVE

RGKTYMLRLVNAALFSEYYYKVAGHRFTVVGVDANYVKPYDTDVLAIAPGETMDVLMVADAPPCRYNMVALS

IQAPAPDPQIQTFVSRGLVRYKNVAVNRTRVCSEQALTPKMPDRHDTATTFFFHGNLTGSLPPGHQSLLRQV

RDRVDERLFITLGQGSICKGGNQEGSCKRGGSNESMLVAYMNNVSFHLSEKTVSLLEARWHRRNTTTINVTV

EELPGRPARVFNFTDLALIPLIPGGKGEELEPTRKATTVRRFAHNATVEVVFQSTAALQSDSNPMHVHGHDF

FVLAQGKGNYDAARDVGRYNLVDPPMKNTVQVPRLGWAAIRFVADNPGMWFMHCHFEYHIATGMATVFQVDD

GPTLDTTLPPPPLDLPKCSHIKE*

>Bradi3g59210.1.p.bdi.32814940/1-579

MAGVTKIPAMLWVLGVVFTFGAAAVGLAEADNTYDFFIKEAKYKRLCRDKTILTVNGQFPGPTITARKGEVV

IVKVHNQGNKNITIHWHGVDQPRNPWYDGPEFITQCPIQPGTSFTYRIVLSDEEGTIWWHAHSDFDRATVHG

AFVIHPKRGSNYPFKAPEREIPIILGEWWKEDVTHMLEQSKRTGGEVDLSDANTINGQPGDLFPCSKDATFK

LPVRTGKTYLLRIINAGLTNDLFFGIAGHPITIIGTDGRYLKPFTVKHIMISPGQTMDALLDTDRAIMGSSN

GRYYMAARTFASNPDLDFNNSTATAILEYMDAPRARRVGLPDFPNLPANLDMDAATEYTAQLRSLASKDHPV

DVPMHVDEEMFITIAVNVFPCAHNKTCEGPRGNSLAASLNNVSFQNPSIDILDAYYSAVDGVYEANFPNQPP

FFFNFTDTTVPVEFEFTKAGTKVKVLEYGSVVEVVFQDTALNGAETHPMHLHGYAFYTVGKGFGIFNKSTDP

AKYNLVDPPYQNTVTVPKAGWTAIRWRATNPGVWFMHCHFDRHTVWGMNTVFIVKDGNTPRSKMMSRPASMP

KC*

>Bradi3g59177.1.p.bdi.32815283/1-578

MVGAKIPTMLWVLGVVFTFGAAAVGLAEANNVHDFIIKEANYPRLCQNKKILTVNGQFPGPTITARRGDVVI

VNVINQGNKNITIHWHGVDQPRNPWYDGPEFITQCPIQPGTNFTYRILLSEEEGTIWWHAHSDFDRASVHGA

FVIHPKNGTYYPFKMPHEEIPIILGEWWKTDVTHLLEESKRTGGEVNLSDANIINGQPGDFYPCSQSNIFKL

PVQTGKTYLLRIINAGLTNDLFYGIAGHLLTIVGTDGRYTKPFTVKHIMISPGQTMDALLEADRAINGSSNG

RYYMAAHTFASNPNLTFTNSTATAILDYVDAPPAKRAGPPDFPNLPNFFDMKAATEYTAQLRSLASKDHPVD

VPMKVDVPMLITIAVNVLPCASNETCEAPDNTRLAASLNNVSFQNPSIDILDAYAQSQNGVYEVNFPDKPPF

FFNFTDTNVPKELEVTKVGTKVKMLNYGEVVEVVFQDTAINGAETHPMHLHGFAFYVVGRGFGNYDAKKDPA

TYNLIDPPYQNTVTVPKAGWTAIRWRASNPGVWFMHCHFDRHTVWGMNTVFIVRDGKKDDEKMFSRPANMPR

C*

>Bradi3g59187.1.p.bdi.32817195/1-579

MVGVTKITMMLWVLGVVFTFGAAAVGLAEANNVHNFYIKEANHPRLCKNKTILTVNGQFPGPTITARRGDVV

IVNVYNQGNKNITIHWHGVDQPRNPWYDGPEFITQCPIQPGTNFTYRILLSDEEGTIWWHAHSDFDRATVHG

AFVIHPKHGSFYPFKMPHKEIPIILGEWWKADVTHLLEESKRTGGEVNLSDANIINGQPGDFFPCSKDNIFK

LPVQTGKTYLLRIINAGLTNDLFYGIAGHLLTIVGTDGRYTKPFTVKHIMISPGQTMDALLEADRAINGSSN

GRYYMAARTFASNTALDFNNSTTTAILEYTDAPPSRRAGTPDFPNLPANLDMNAATEYTAQLRSLASKDHPV

DVPMHVDHPMLITIAINVLPCAPNQTCDGPNGNRLAASLNNVSFQNPSIDILDAYYSSVNGVFEASFPNKPP

FFFNFTDTVVPPELEVTKVGTKVKMLNYGDVVEVVFQDTTINGAETHPMHLHGFAFYVVGRGFGNYDKLKDP

ATYNLIDPPYQNTVTVPKAGWTAIRWRATNPGVWFMHCHFDRHTVWGMNTVFIVKDGKTPDTKMMKRPPSMP

RC*

>Bradi3g02290.2.p.bdi.32819041/1-585

MAATVAFFFFAAILAAAAAGGGDAALVEHTFIVSQVRLNRLCNDTLVTVVNGQLPGPTIEVREGDSVVVHVI

NKSPHGLTIHWHGVKLQLNCWADGAGMITQCPIRPNNNFTYRFDIVEQEGTLWWHAHVASLRATIHGALIIR

PRPGPSSYPFPKPEKEIPIVIGEWWEMDLVELDMRLRNGNLFDVPRAATINGQTGDLYNCSAHVDFAGAIKE

SNILNVEHGKTYLLRIVNAALNSEYYLKIAGHKFTVVGADANYVKPYTTDVITIAPGETVDALLVTDAHPGG

RYYMIAMAYQPPKPAKQFPLFLSRGIVQYYDNNASPRKEEEALPNTPMAPEMPDQHDAVPSFYFYGNLTSLQ

PHPLPTIVDERLFYALDAGYFCREGGSSCQNVSNIVATINNVSFQLPETTPLLQAHYYNNMKSGIGTLPDGS

PRMFNYSMSLAPTSKATSVRKLRYNTTVEIVFQSPVIADSYSNPMHLHGHDFFVLAQGFGKFDEKKDVKTYN

LVDPPVRNTVHVPIYGWAAIRFVTKNPGVWYLHCHYGHHSSTGMAVALVVENGPTLDTTLPPPPADFPSCDN

YISMLANE*

>Bradi3g02290.1.p.bdi.32819042/1-579

MAATVAFFFFAAILAAAAAGGGDAALVEHTFIVSQVRLNRLCNDTLVTVVNGQLPGPTIEVREGDSVVVHVI

NKSPHGLTIHWHGVKLQLNCWADGAGMITQCPIRPNNNFTYRFDIVEQEGTLWWHAHVASLRATIHGALIIR

PRPGPSSYPFPKPEKEIPIVIGEWWEMDLVELDMRLRNGNLFDVPRAATINGQTGDLYNCSGAIKESNILNV

EHGKTYLLRIVNAALNSEYYLKIAGHKFTVVGADANYVKPYTTDVITIAPGETVDALLVTDAHPGGRYYMIA

MAYQPPKPAKQFPLFLSRGIVQYYDNNASPRKEEEALPNTPMAPEMPDQHDAVPSFYFYGNLTSLQPHPLPT

IVDERLFYALDAGYFCREGGSSCQNVSNIVATINNVSFQLPETTPLLQAHYYNNMKSGIGTLPDGSPRMFNY

SMSLAPTSKATSVRKLRYNTTVEIVFQSPVIADSYSNPMHLHGHDFFVLAQGFGKFDEKKDVKTYNLVDPPV

RNTVHVPIYGWAAIRFVTKNPGVWYLHCHYGHHSSTGMAVALVVENGPTLDTTLPPPPADFPSCDNYISMLA

NE*

>LOC_Os07g01110.1.osa.33116765/1-584

MAPSLGSGSTRILLIVSLLLCLRQQAVVDAAIVEHTFHVGNLTVERLGQRQVITAVNGQFPGPKVEARNGDT

LLVRVVNNSPYNITIHWHGVLQRLSAWADGPAMVTQCPILPGSGAGSSYTYRFNVTGQEGTLWWHAHVSFLR

ATVYGALLIRPRPGVPYPFPAPHAEHTLLLGEWWNASATLVDVERQAFLTGGQPANSVALTINGMPGLSHAH

KEMHHLRVARGNTYLLRLVNAALNYQLFFKVAAHNFTVVAVDACYTDPYHTDVIVIAPGQTVDALMHAGAAP

GRRYYVAAQVYQSIANATYSATARALLRYDDDAKDAAKTIIMSPRMPVLNDSATAQRFYGSLTGLLRDGKPT

VPQRVDTRMVVTYGLAIAPCLPAQTLCNRTRGSLAASMNNVSFQLPATMSLLEASRSRSSGVYTRDFPDRPP

VMFDFTNAAAVNRNMSLMVTSKGTRVKALRYNETVEVVLQNTAVLGTENHPLHLHGFNFYVLAQGTGNYYYL

IRKKKIRKNLVNPQQRNTIAVPPGGWAVIRFTADNPGVWLMHCHLEAHLPFGLAMAFDVQDGPTPDAMLPPP

PNDYPPC*

>LOC_Os01g62600.1.osa.33118821/1-548

MGTPRGLRNAGSSSSACRFLAAFAVLLALPTLTAGLTRHYTFNVQMTNVTRLCVTKSIPTVNGQFPGPKLVV

REGDRLVVKVHNHMNYNVSFHWHGILQLRNGWADGPSYITQCPIQGGGSYVYDFTVTGQRGTLWWHAHFSWL

RVHLYGPLVILPKRGEGFPFPRPYKELPPIMFGEWFNADTEAVINQALQTGAGPNISDAYTFNGLPGPTYNC

SSKDTYKVKVQPGRTYLLRLINSALNDELFFGIANHTLTVVEADANYVKPFTAKTLVISPGQTMNLLLTTAP

NPGSPVYAMAIAPYTNTQGTFDNTTAVAVLEYAPTRASATGNNNLPLPPLPRYNDTNAVANFSSKFRSLATA

RYPARVPRAVDRHVLFTVGLGTDPCPSNQTCQGPNGTKFAASINNNSFVRPRVALLEAHCQRRVVPLAFNTS

VELVLQGTSIQGAESHPLHMHGFNFFVVGQGFGNYDPVNDPANYNLVDPVERNTVSVPTGGWVAVRFLADNP

GVWLMHCHFDVHLSWGLSMAWLVNDGPLPSQKMLPPPSDLPKC*

>LOC_Os01g61160.1.osa.33119297/1-568

MASSSSSRLLFLLSCSVLALLAGAEVHHHEFIVQETPVKRLCKTHNVITVNGQLPGPTLEVREGDTVVINVV

NHAQYNVTIHWHGIRQFRTGWADGPEFVTQCPIKPGGSYKYRFTIEGQEGTLWWHAHSSWLRATVYGALIIR

PRENKTYPFEKPAREVPLILGEWWDADPIQVIREAQRTGAAPNISDAYTINGQPGDLYNCSKEETTAVPVKP

GETALLRFINAALNQELFVSIAQHKMTVVGVDASYTKPFTTSVLMIAPGQTTDVLVTMDQAPTRYYLAARAY

DSAQGVAFDNTTTTAVIEYDCGCATDFGPSIPPAFPVLPAFNDTNTATAFAAGIRSPHEVKIPGPVDENLFF

TVGVGLFNCEPGQQCGGPNNTRFTASMNNISFVFPQTTSLLHAHYYGIPGVFTTDFPAYPPVQFDYTAQNVP

RYLWQPVPATKLYKLKFGSVVQIVLQDTSIVSPENHPIHIHGYDFYILAEGFGNFDPKKDAKKFNYVDPPQR

NTVAVPTNGWAVIRFVADNPGVWLMHCHLDVHITWGLAMAFLVEDGYGKLETLEAPPVDLPMC*

>LOC_Os01g63200.1.osa.33120085/1-555

MASAAMLVPLVLVLCTAAASAAVVEHTFKVGGTKITQLCMNSVIYTANQQLPGPTIEVTEGDTLVVHAVNDS

PYPLSLHWHGVYQLRSGWNDGANKITQCPIQPSGNFTYRFNITGQEGTLWWHAHSSLLRATIYGALIIKPRN

GPSGYPFPEPYEEIPILLGEWWNRNVDDVENDGYLTGLGPQISDALTINGMPGDQNRCKGSAMYEVEVEYGK

TCLLRIINAAVNVELFFKVAGHTFTVVAADASYTKPYATDVIVIAPGQTVDALMNTTASPGRYYMAAHVFDS

KTVAVPFDQSTATGIVKYKGVPNYAPAAMPSLPPHDDVVTAGRFYWSLTGLARPSDPGVPTTVDHNMVVTFG

LDQAPCAPNQTKCSGFALVAAMNRNSFQFPDQKVSLLEALYKGVPGVYSEDFPDFPPPMQGFRKATAVKKVK

YNDVVEVVLQSEQYSSTLGTENHPIHLHGFDFYLLAQGLGRFNPSMKSKYNLVDPQVRNTVAVPAGGWAVIR

FMANNPGMWFMHCHLDAHLPLGLAMVFEVLNGPAPNLLPPPPVDHPKCHG*

>LOC_Os01g44330.1.osa.33120305/1-563

MASAASSLPLLVSSLLLALFALGAHADVKRYQFDIVMSNVSRLCHEKAMVTVNGSYPGPTIYAREGDRVIVN

VTNHVKHNMTIHWHGLKQRRNGWADGPAYVTQCPIGSGGSYVYDFNVTRQRGTLWWHAHIAWMRATVHGAIV

ILPAAGVPYPFPKPDDEAEIVLGEWWHADVETVERQGSMLGMAPNMSDAHTINGKPGPLVPFCSEKHTYALQ

VQSGKTYLLRIINAAVNDELFFSIAGHNMTVVEIDATYTKPFAASTVQLSPGQTMNVLVSADQSPGRYFMVA

KPFNDVPIPADNKTATAILQYAGVPTSVVPALPQTMPATNSTGSVAAFHDKLRSLNSPRYPADVPLAVDRHL

LYTIGLNIDPCETCLNRSRLAASLNNITFVMPRTALLQAHYYGQKGVFAADFPDRPPARFNYTGVPLTAGLG

TSLGTRLSKIAYNATVELVLQDTNLLSVESHPFHLHGYNFFVVGRGVGNFDPAKDPAKYNLVDPPERNTVGV

PAGGWTAIRFRADNPGVWFLHCHLEVHTSWGLKMAFLVEDGSGPDESVLPPPKDLPKC*

>LOC_Os01g63190.1.osa.33120401/1-560

MVIPWCSSMMRLLWFLFALLLARSVADAATANYTFTVESMRVSRLCNSTDIIAVNGLLPGPMIEVNEGDAVA

VEVINGSPYNLTIHWHGILQLLTPWADGPSMVTQCPIQPNSSYTYRFNVTGQEGTLWWHAHSSFLRATVYGA

LIIRPRNGSAYPFPAPDQEVPIVLGEWWSRNVVDIESDAVSSGQLPRESDAFTVNGVTGELYQCANDTFTVD

VQPNTTVLLRVINAGLNTHLFFKVAGHAFTVVAVDACYTANYTTDTLVLAPGHTVDALMVTNASAGSYYMAV

QAYDSLSPTTMAVTDDTTATAIVHYNTTSTKKNATPVMPTMPQSSDSATANAFYFGLRGPPSPSAPAVPTKV

DVNMTIELGLGQLPCDSTQSSCSGKSVAAAMNGVSFRLPSQMSLLEAQFNRTPGVYTADFPDAPQPSGTPMV

EGTKVRRLKYNSTVEIVLQNPTAFPSENHPIHLHGFNFFVLAQGLGNFTPGNVSGYNLVDPVSRNTLAVPTG

GWAVIRFVANNPGMWFFHCHLDAHVPMGLGMVFAVDNGTTPDSFLPPPPADLPKC*

>LOC_Os01g62490.1.osa.33122519/1-578

MAISYLLRSSILAVAALLLFSVNLAKGDIREYQFDVKTTNVTRLCSSKSIVTVNGQFPGPTVFAREGDLVVI

RVINHSPYNMSIHWHGIRQLRSGWADGPAYITQCPIQPGGSYVYKYTITGQRGTLWWHAHISWLRATVYGPI

IILPKAGVPYPFPAPDKEVPVVFGEWWKADTEAVISQATQTGGGPNVSDAFTINGLPGPLYNCSAKDTFKLK

VEAGKTYMLRLINAALNDELFFSIAGHTLTVVDVDAVYVKPFTVDTLLITPGQTTNVLLTTKPSYPGATFYM

LAAPYSTAMSGTFDNTTVAGILEYEDPSSHSTAAFNKNLPVLRPTLPQINDTSFVSNYTAKLRSFATAEYPA

NVPQQVDTRFFFTVGLGTHPCAVNGTCQGPNGSRFAAAVNNVSFVLPSTALLQSHYTGRSNGVYASNFPAMP

LSPFNYTGTPPNNTNVSNGTRLVVLPYGASVELVMQGTSVLGAESHPFHLHGFNFFVVGQGFGNFDPVNDPA

KYNLVDPVERNTVGVPAAGWVAIRFLVDNPGVWFMHCHLEVHVSWGLKMAWVVQDGSLPNQKILPPPSDLPK

C*

>LOC_Os01g63180.1.osa.33124840/1-483

MVTQCPIRPGHRYTYRFAVAGQEGTLWWHAHSSYMRATVYGALVIRPRRAGGYPFPTPYEEKTVLLGEWWNG

DPVALESQSFSTGIPAPNADAYTINGMPGDSYLCPETTNRIAKFEVRRDKTYLLRIINAALNTAFFFKVAGH

TFTVVAADASYTEPYATDVIVIAPGQTVDALMAADASPGCYHMAISSYQSAIPFPPRPAGFNGNTSTAIVEY

VDATATTDAGSPVLPVMPKPNDTYTANQFYTSLTALIRPGRRTVPLTVDTRMLVTVGLGFSSCQPEQTQCNR

SAPVVLANMNNVSFALPNTVSMLEALYRNTADGVYTRDFPDQPPVAFDYTSRGLLGNSPLASTGSPSTKVKT

LRYNATVEMVLQNTALVGLESHPMHLHGFNFFVVAQGFGNNDGEAAGAGEFNLVNPQERNTVAVPTGGWAVI

RFVADNPGMWAMHCHIDSHFAIGLAMVFEVESGPTPGTTLPPPPPDLPQC*

>LOC_Os01g62480.1.osa.33124985/1-580

MTMAISSALPSPLLLAASLLLLIVQAQGITRHYEFNVQMANATRLCNTKSMVTVNGQCPGPELVAREGDRVV

IRVTNNVAHNISLHWHGVRQVRTGWADGPAYITQCPIQTGQSYVYNFTVAGQRGTLWWHAHISWLRATVYGA

LVILPKLGVPYPFPAPHKEVPVIFGEWWNADTEEVVNQAVQTGGGPNVSDAFTINGLPGPLYNCSAQDTFKL

KVKPGKTYMLRLINAALNEELFFAVANHTLTVVEVDAVYVKPFTVDTLVISPGQTTNVLLTAKPYYPGANFY

MSAAPYSTARPGTFGNTTVAGILEYENPAMSPSAASFVKGLPLFKPTLPQLNDTDFVTNFTDKLRSLATPEY

PAAVPQSVDKRFFFTVGLGTLPCPANMTCQGPNNTQMAASMNNVSFVLPARALLQSHFTGLSSGVYAPDFPV

APLSPFNYTGTPPNNTNVKTGTKLLVLRYNTSVELVMQDTSILGIESHPLHLHGFNFFVIGQGFGNYDAVND

PAKFNLVDPVERNTVGVPAGGWVAIRFLADNPGVWFMHCHLEAHTTWGLRMAWLVLDGSHPNQKLLPPPSDL

PKC*

>LOC_Os01g27700.1.osa.33125405/1-553

MGTAKIPALLWFLLAGLVLALAVNPAHGAKTRHYDFFITETNYTRLCHEKSILTVNGQFPGPTIYARKGDFI

IVNVHNNGNKNITIHWHGVDQPRNPWSDGPEFITQCPIRPGGNFTYQVILFEEEGTLCSILQCDAMQCFWWN

DDVEHVLDKAKRIGGDVEPSDTNTINGQPGDMFPLCSRDDTFKVAVQQGNTYLLRVINAGLTNDMFFAIAGH

RLTVVGIDARYTKPITVDYIMIAPGQTMDVLLKANRTLGSNSRYYMAARTFITLPVDTIRFNNSTATAIVEY

TDSAVARPVGPPEFPVLLPAIKDEDAAMAFVKQLRSLGNQDHPVHVPKQVDEHMLIDIDINFLPCDANNATN

KLCEGPQGNRFAASLNNVSFQNPAIDVLDAYYYGSGRGVYEENFPNKLTVIVNPTGDINGGGPLLTKRGTKV

KVLEYGTVVEVVFQDLSIENHPMHLHGFTFYVVGRGSGTFDERRDPATYNLIDPPFQNTVSVPKSSWAAIRF

RADNPGVWFMHCHFDRHVVWGMDTMFIVKDGKTPQAQMLPRPPNMPEC*

>LOC_Os03g16610.1.osa.33131068/1-579

MGARCLALLLLYGTLLLLLLLPQLPLAGAATRYYTFNVKLQNVTRLCNTRAIPTVNGKFPGPKIVTREGDRV

VVKVVNNIKDNITIHWHGVRQMRTGWSDGPAYVTQCPIQTGQSYVYNFTINGQRGTLFWHAHVSWLRSTLYG

PIIILPKAGLPLPFTEPHKDVPIIFGEWFNADPEAIVAQALQTGGGPNVSDAYTINGLPGPLYNCSSKDTFR

LKVQPGKMYLLRLINAALNDELFFSVANHTLTVVDVDASYVKPFDTDVVLITPGQTTNVLLRAKPTAEAAGA

THLMMARPYATGRPGTYDNTTVAAVLEYAPPGHIKSLPLLRPSLPALNDTAFAAGFAAKLRSLACPDYPSNV

PRRVDKPFFFAVGLGTTPCPGSNNQTCQGPTNTTKFTASINNVSFDMPTTALLQAHYTGQSAGVYTADFPAS

PLEPFNYTGTPPNNTNVSNGTRVVVLPYNASVEVVLQDTSILGAESHPLHLHGFDFFVVGQGTGNYDPSKHP

AEFNLVDPVQRNTVGVPAGGWVAIRFFADNPGVWFMHCHLEVHTTWGLKMAWVVNDGPLPEQKLMPPPSDLP

MC*

>LOC_Os02g51440.1.osa.33139001/1-580

MGTAKLPALLWLLAGVVLALAVNPAHGAKTRHYDFFITETNYTRLCHEKSILTVNGQFPGPTIYARKGDLVI

VNVHNNGNKNITIHWHGVDQPRNPWSDGPEFITQCPIRPGGNFTYQVILSEEEGTLWWHAHSDFDRATVHGA

IVIHPKRGTTFPFKKPDKEIPVILGEWWNDDIEHVLDKAQLLGGDVDPSNANTINAQPGDMFPCSRDDTFKV

AVQQGNTYLLRIINAGLTNDMFFAIAGHRLTVVGIDARYTKPLTVDYIMIAPGQTMDVLLEAKRTLGSNSRY

YMAARTFITLPLDTIPFNNSTATAIVEYTDSVTARPVGPPEFPVQLPAIKDENAAMAFVTQLRSLGNQEHPV

HVPTHVDEHMLIDIDINVLPCDPTNMAEKCKEGPQGNRFAASLNNVSFQSPAIDVLDAYYYSSGHGVYEEDF

PNKPTAFVDPPVNNGSGPLMTKRGTKVKVLEYGTVVEVVFHDLSSENHPMHLHGFAFYVVGRGNGTFDESRD

PATYNLVDPPFQNTVSVPRSGWAAIRFRADNPGVWFMHCHFDRHVVWGMDTVFIVKDGKTPQAQMLPRPPNM

PQC*

>LOC_Os12g15680.1.osa.33152344/1-599

MKSPVHLEHPFGTQIVKDNMARSWSLLLLPFALALVASVAQAAVVEYTFNVGNLSISQLCQQEMIITAVNGQ

LPGPTIVATEGDTVVVHMVNESPYNMTIHWHGIFQRGTPWADGPAMVTQCPVRPGGNYTYRFNVTGQEGTLW

WHSHFSFLRATVYGALIIKPRGGAKAYPFPVPDEEVVVILGEWWKTNVYDLQQRSLVTGNPAPHADAYTING

KPGDFYNCSAPNQTHKFELKQNKTYMLRIINAALNTPLFFKVANHSFNVVAADACYTKPYKTDVVVISPGQT

VDALLVPDAGVAAAVGGRYYMAVIPYNSAVNAADPSFLYSLTNSTAIVEYGGGPATSPPMVPDMPEYNDTAT

AHRFLSNMTALVPNRVPLAVDTHMFVTVSMGDTFCGPEQTMCMPDDKGTIFASSMNNASFILPNTTSMLEAM

YKGSIDGVYTRDFPDTPPIVFDYTADASDDNATLKHTFKSTKVKTLKYNSTVQMVLQNTRLVSKESHPMHLH

GFNFFVLAQGFGNYNETTDPAKFNLVDPQERNTVAVPTGGWAVIRFVADNPGVWFMHCHFDAHLEFGLGMVF

EVQNGPTQETSLPPPPSDLPQC*

>LOC_Os12g01730.1.osa.33153944/1-568

MGSRGCSCWLLSLALLCSLAAAKEQYHEFVIRETTVKRLCKSQSIMTVNGQFPGPTLEIKEGDSLIINLINR

GRYNVTLHWHGVRQMRTGWSDGPEYVTQCPVRPGQSYRYRFTVAAQEGTLWWHAHSSWLRATVYGALLIRPR

DGTSYPFHVQPTRELAPILLGEWWDMNPVDVVRAATRTGAAPNISDALTVNAQPGDLYSCSSHDTAFFPVTS

GETNLLRFINAALNTELFVSLAGHNMTVVAADASYTKPYTTSLLLLAPGQTTDVLVTFDQPPGRYYLAARAY

ASAQGVPFDNTTTTAIFDYGAANNASSAAIAMPTLPAYNDTTAATAFTTNLRGLRKAELPSRVDESLFFTVG

VGLFNCTNATAQQCGGPNGTRFAASINNVSFVLPSSTSILQAHHHGAPGGVFTADFPASPPVQFDYTAQNVS

RALWQPVPGTKVYKLKYGSAVQVVLQGTNIFAGENHPIHLHGYDFYILAEGLGNFDAGADTAKFNMEDPPMR

NTVGVPVNGWAVIRFVADNPGVWLMHCHLDVHITWGLAMAFLVDDGVGELQSLEAPPPDLPLC*

>LOC_Os12g15920.1.osa.33154335/1-659

MRFTLEAAHILAPGSIAAKPGRIKLTGTPASHHIWTCTGAKQISQAEKIHLTQVWSKFPTSNGTQTFRPSEV

TADELQTILVWWYFEGYQRWQLPLPVEIAQAAVVEHTFNVGNFSISQLCQPPLIITAVNGQLPGPTIYAREG

DTVVVHLVNTSPYSMTLHWHGVLQRGTPWADGPAMVTQCPVQPGGNYTYRFNVDGQEGTLWWHAHVSFHRAT

VYGALVIRPRGGAKAYPFPKPDKEHVVILGEWWNATVYDMERMAFLTGIPAPHADAYTINGKPGDFYNCSAP

NQTAKFEVRQNGTYLLRIINAGMNTPLFFKVAKHRLTVVGADACYTKPYKTDVVVVSPGQTVDALMVASAAV

GRYYMAASPYDSAIPQGPPFSDTTATAILQYAGARRKTVRWRPPVLPRRPPVNDTATAHRFFSGMTALLRHG

KPSAVPLAVDTHMYVTVGLGVSLCQPEQLLCNRSAPPVFSSSMNNASFVVPKNTSLLEAHFRREPAGVYTRD

FPDTPPVVFDYTGDESDNATMQFTTKSTKVKTLRYNETVEMVLQNTRLIAKESHPMHIHGFNFFILAQGFGN

YDKRRAERRFNLVDPQERNTIAVPTGGWAVIRFVADNPGMWYMHCHFDAHISLGLAMVLEVLDGPTPETSVP

PPPADLPRCS*

>LOC_Os05g38410.1.osa.33157295/1-550

MAAASSVLRCCLLVAALMTLSAMGAEAITRQYLFDVQTTSVTRLCSTKSIVTVNGQYPGPTLFAREGDHVEV

TVVNHSPYNMSIHWHGIRQLLSGWADGPSYITQCPIQPGGSYVYRFTITGQRGTLWWHAHISWLRATVHGPM

VILPPAGVGYPFPAPHEEVPIMFGEWWNNDTEAVISQALQTGGGPNISDAYTLNGLPGPLYNCSAQDTFKLK

VKPGKTYMLRLINAALNDELFFSIANHTLTVVDVDALYVKPFTVDTLIIAPGQTSNVLLTAKPTYPGASYYM

LARPYTTTQGTFDNTTVAGVLEYDDPCPTTAAGKIVPIFSPTLPQINDTNAVSNFTAKLRSLASAGYPAAVP

QQVDHRFFFTVGLGTHPCAVNGTCQGPNGSRFAASINNVSFVLPATALLQSHFAGKSKGVYASNFPYYPLNP

FNYTGTPPNNTNVMNGTKVLVLPYGANVELVMQDTSILGAESHPLHLHGFNFFVVGQGFGNFDPINDPAKFN

LYDPVERNTVGVPAGGWVAIRFHADNPGMYSEPHRFPCDSTFAPT*

>LOC_Os05g38390.1.osa.33157984/1-514

MATVTRLCVTKSVPTVNGQFPGPKLVVREGDTLVIRVTNNINNNVTFHWHGIRQVRSGWADGPAYITQCPIR

SGGSYVYRFTVTGQRGTLWWHAHFSWLRATLYGPLVILPPRGVAYPFPKPHREVPLLLGEWFNADPEAVIKQ

ALQTGGGPNVSDAYTFNGLPGPTYNCSSSNDTFKLRVRPGKTYLLRLINAALNDELFFGVANHTLMVVQADA

SYVKPFAATALVISPGQTMDVLLTAAANNPPSRSFAIAVAPYTNTVGTFDNTTAVAVLEYYGAATSAAALRS

LPLPSLPAYNDTGAVANFSASFRSLASAQYPARVPRTVDRHFFFAVGLGADPCQSPVNGTCQGPNNTRFAAS

MNNVSFVMPRTSLLQAHYQRRYNGVLAANFPAAPRTPFNYTGTPPNNTFVTHGTRVVPLSFNTTVEVVLQDT

SILGAESHPLHLHGYDFYVVGTGFGNYDASNDTAKYNLVDPVQRNTISVPTAGWVAIRFVADNPGWLPALYL

YLHLKREFL*

>LOC_Os05g38420.1.osa.33159283/1-575

MAAASSVLRCCLLVAALMTLSAMGAEAITRQYLFDVQTTSVTRLCSTKSIVTVNGQYPGPTLFAREGDHVEV

TVVNHSPYNMSIHWHGIRQLLSGWADGPSYITQCPIQPGGSYVYRFTITGQRGTLWWHAHISWLRATVHGPM

VILPPAGVGYPFPAPHEEVPIMFGEWWNNDTEAVISQALQTGGGPNISDAYTLNGLPGPLYNCSAQDTFKLK

VKPGKTYMLRLINAALNDELFFSIANHTLTVVDVDALYVKPFTVDTLIIAPGQTSNVLLTAKPTYPGASYYM

LARPYTTTQGTFDNTTVAGVLEYDDPCPTTAAGKIVPIFSPTLPQINDTNAVSNFTAKLRSLASAGYPAAVP

QQVDHRFFFTVGLGTHPCAVNGTCQGPNGSRFAASINNVSFVLPATALLQSHFAGKSKGVYASNFPYYPLNP

FNYTGTPPNNTNVMNGTKVLVLPYGANVELVMQDTSILGAESHPLHLHGFNFFVVGQGFGNFDPINDPAKFN

LYDPVERNTVGVPAGGWVAIRFHADNPGVWFMHCHLEVHMSWGLKMAWLVLDGSRPDQKLPPPPLDLPKC*

>LOC_Os11g48060.1.osa.33160442/1-587

MHCTALSPALSSPSPAAGHAANMAVLPESRRLSLLLMAACFLLQALSAHAITRHYKFNVVMRNMTRLCSTKP

ILTVNGKFPGPTLYAREGDNVLVKVVNHVAHNVTIHWHGVRQIRTGWYDGPAYITQCPIQPGSSFLYNFTIT

GQRGTLLWHAHINWLRATVHGAIVILPKLGVPYPFPAPHKEAVIVLGEWWKEDTETVINQAMQLGVGPNISD

SHTINGHPGPLSECASSQDGFKLSVENGKTYMLRIINAALNDDLFFKVAGHELTVVEVDAVYTKPFKTDTLL

ITPGQTTNVLVRANQGAGRYLLSVSPFMDAPVQVDNKTGTATLHYANTVSSSMASLTLVKPPPQNATHIVSK

FTDSLHSLNSKEYPANVPQTVDHSLLLTVGVGVNPCPSCINGTRVVGTINNVTFIMPSTPILQAHYYNIPGV

FTEDFPATPLHKFNYTGSGPKNLQTMNGTRVYRLPYNASVQVVLQDTGIISPESHPIHLHGFNFFVVGKGVG

NYNPRTSPSTFNLIDPIERNTIGVPTGGWTAIRFRSDNPGVWFMHCHFEVHTSWGLKMAFVVDNGKRPSETL

IPPPKDLPQC*

>LOC_Os11g47390.1.osa.33163692/1-584

MGIAKIPAVLWLLACAVLTFAVAISPAHGGRTRRHYDFFITETNYRRLCHEKSVLTVNGQFPGPTIYARKGD

LVIVNVYNHGNKNITIHWHGVDQPRNPWSDGPEFITQCPIRPDGKFTYQVIMSEEEGTLWWHAHSDFDRATV

LGAIVVHPKHGDTFPFKRPDKEIPIILGEWWKNDVNHLLEEMKRIGEDVKPSDANTINGQPGDMFPCSRDDT

FKVAVEHGNTYLLQVINAGLTNDMFFAVSGHRLTVVGIDARYTKPLTVEYIMIAPGQTMDLLLEANRSLGSK

SNSRYYMAARTFITLPVPIPFNNSTATAVVEYYTGDSGAGPPDFPAVLPSLDDVDAAMAFLRQLRSLGSKDH

PVHVPTHVDEHMLIDLAINFLPCNATNATDTACKGPKGNTTRFAASLNNVSFVSPAIDVLHAYYYGSGRGVY

EDDFPNNPAPVFVNLTGDNDRPGVTKHGAKVKVLEYGTVVEVVFQDTSFESHPMHLHGFAFYVVGLGSGKFD

DRRDPATYNLLDPPYQSTVSVPKAGWAAIRFRADNPGVWFMHCHFDRHMVWGMNTVFIVKDGKTPQAQMLPR

PPNMPKC*

>LOC_Os11g01730.1.osa.33163704/1-568

MPSRGCSCWLLSLALLCSLAAAKEQYHEFVIRETTVKRLCKSHNIMTVNGQFPGPTLEINEGDSLIINLINR

GRYNMTLHWHGVRQMRTGWSDGPEYVTQCPVRPGQSYRYRFTVAAQEGTLWWHAHSSWLRATVYGALLIRPR

DGTSYPFDVQPTRELAPILLGEWWDMNPVDVVRAATRTGAAPNISDALTVNAQPGDLYSCSSHDTAVFPVTS

GETNLLRFINAALNTELFVSLAGHNMTVVAADASYTKPYTTSLLLLAPGQTTDVLVTFDQPPGRYYLAARAY

ASAQGVPFDNTTTTAIFDYGAANNASSAAIAMPTLPAYNDTTAATAFTTNLRGLRKAELPSRVDESLFFTVG

VGLFNCTNATAQQCGGPNGTRFAASINNVSFVLPSSTSILQAHHHGAPGGVFTADFPANPPVQFDYTAQNVS

RALWQPVAGTKVYKLKYGSAVQVVLQGTNIFAGENHPIHLHGYDFYILAEGLGNFDAGADTGKFNVEDPPMR

NTVGVPVNGWAVIRFVADNPGVWLMHCHLDVHITWGLAMAFLVDDGVGELQSLEAPPPDLPLC*
