# Supplementary figures and images for: Inactivation of LACCASE8 and LACCASE5 genes in Brachypodium distachyon leads to severe decrease in lignin content and high increase in saccharification yield without impacting plant integrity
Source: Biotechnol Biofuels. 2019 Jul 15;12:181. doi: 10.1186/s13068-019-1525-5 (PMC6628504; doi:10.1186/s13068-019-1525-5)

## Slide 1
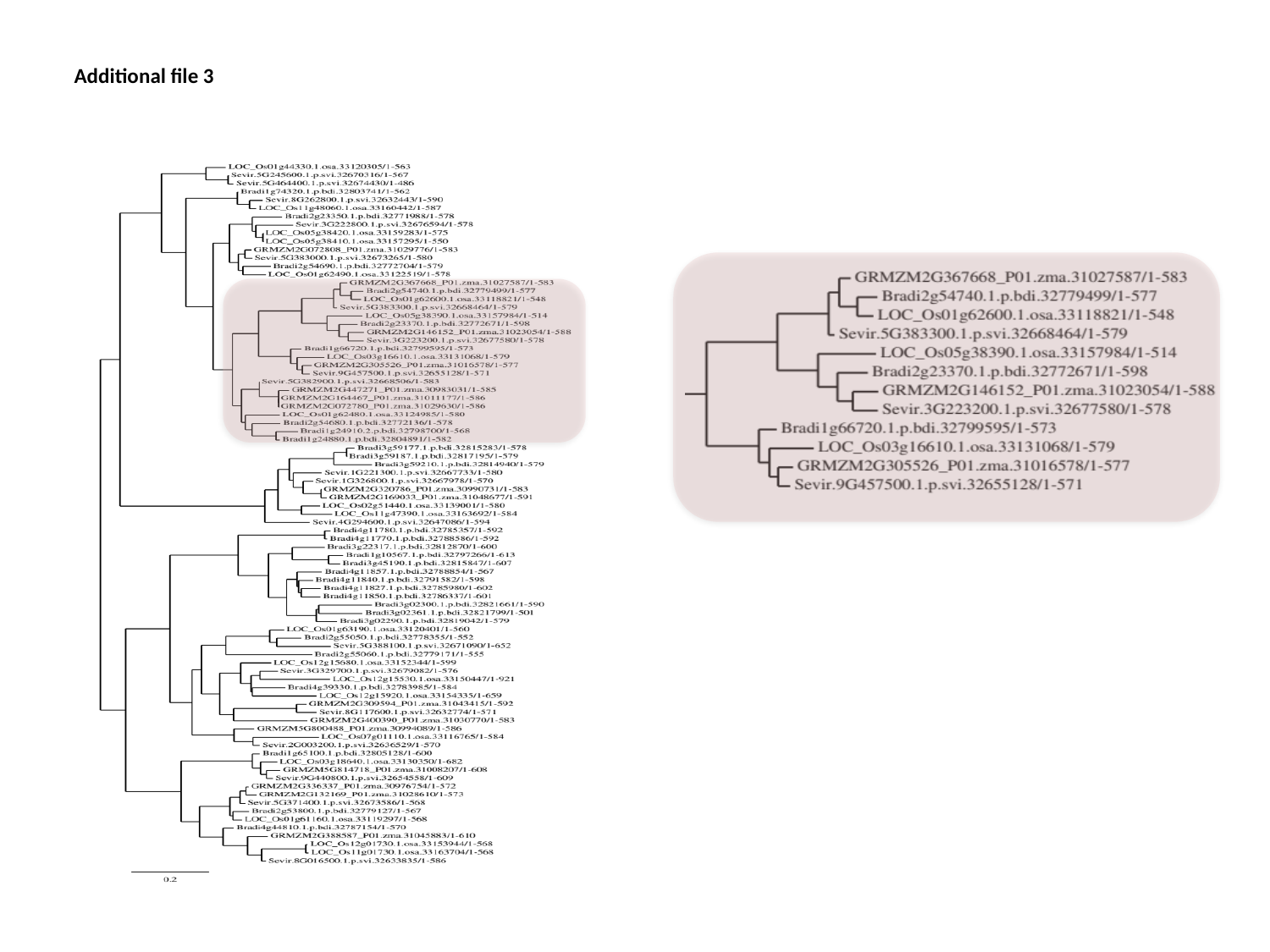

Additional file 3

Supplement: Supplementary file 3 — Additional file 3. Phylogeny tree reconstructed with Brachypodium, maize, rice and Setaria LACCASES. The phylogenetic tree was reconstructed using the maximum likelihood method implemented in the PhyML program. The putative proteins sequences used to reconstruct the tree are available in Additional files 1 and 2. Branch length is proportional to the number of substitutions per site and represents evolutionary distance as indicated by the scale bar. The colored cluster highlights the positions of LACCASE 5 and 8 and their closest orthologs in other species. [file 13068_2019_1525_MOESM3_ESM.pptx]
