# Supplementary material for: Inactivation of LACCASE8 and LACCASE5 genes in Brachypodium distachyon leads to severe decrease in lignin content and high increase in saccharification yield without impacting plant integrity
Source: Biotechnol Biofuels. 2019 Jul 15;12:181. doi: 10.1186/s13068-019-1525-5 (PMC6628504; doi:10.1186/s13068-019-1525-5)
Supplement: Supplementary file 4 — Additional file 4. Genes co-expressed with LAC5 (LAC8 is in bold red). The table containing genes found in the co-expression network was made with the search tool from PlaNet (http://www.gene2function.de/) using LAC5 gene (Brad1g66720) as a bait. [file 13068_2019_1525_MOESM4_ESM.docx]

**Additional file 4**

| Probeset/other ID | GeneID | Description | Label(s) present |
| --- | --- | --- | --- |
| [Bradi2g18447.1](http://aranet.mpimp-golm.mpg.de/responder.py?name=gene!Bdi!19886) | [bradi2g18447](http://aranet.mpimp-golm.mpg.de/responder.py?name=gene!Bdi!19886) | sulfotransferase domain containing protein, expressed | [ORTHO017557](http://aranet.mpimp-golm.mpg.de/responder.py?name=fam!fam!ORTHO017557) [HOM000207](http://aranet.mpimp-golm.mpg.de/responder.py?name=fam!fam!HOM000207) |
| [Bradi3g38950.1](http://aranet.mpimp-golm.mpg.de/responder.py?name=gene!Bdi!3935) | [bradi3g38950](http://aranet.mpimp-golm.mpg.de/responder.py?name=gene!Bdi!3935) | methyladenine glycosylase, putative, expressed | [Adenine_glyco](http://aranet.mpimp-golm.mpg.de/responder.py?name=fam!fam!Adenine_glyco) [ORTHO009146](http://aranet.mpimp-golm.mpg.de/responder.py?name=fam!fam!ORTHO009146) [HOM000675](http://aranet.mpimp-golm.mpg.de/responder.py?name=fam!fam!HOM000675) |
| [Bradi2g11500.1](http://aranet.mpimp-golm.mpg.de/responder.py?name=gene!Bdi!20894) | [bradi2g11500](http://aranet.mpimp-golm.mpg.de/responder.py?name=gene!Bdi!20894) | kelch motif family protein, putative, expressed | [Kelch_1](http://aranet.mpimp-golm.mpg.de/responder.py?name=fam!fam!Kelch_1) [Kelch_2](http://aranet.mpimp-golm.mpg.de/responder.py?name=fam!fam!Kelch_2) [ORTHO011617](http://aranet.mpimp-golm.mpg.de/responder.py?name=fam!fam!ORTHO011617) [HOM000141](http://aranet.mpimp-golm.mpg.de/responder.py?name=fam!fam!HOM000141) |
| [Bradi1g75350.1](http://aranet.mpimp-golm.mpg.de/responder.py?name=gene!Bdi!12951) | [bradi1g75350](http://aranet.mpimp-golm.mpg.de/responder.py?name=gene!Bdi!12951) | RING finger and CHY zinc finger domain-containing protein 1, putative, expressed | [zf-C3HC4](http://aranet.mpimp-golm.mpg.de/responder.py?name=fam!fam!zf-C3HC4) [zf-CHY](http://aranet.mpimp-golm.mpg.de/responder.py?name=fam!fam!zf-CHY) [HOM000317](http://aranet.mpimp-golm.mpg.de/responder.py?name=fam!fam!HOM000317) |
| [Bradi2g26770.1](http://aranet.mpimp-golm.mpg.de/responder.py?name=gene!Bdi!7393) | [bradi2g26770](http://aranet.mpimp-golm.mpg.de/responder.py?name=gene!Bdi!7393) | annexin, putative, expressed | [Annexin](http://aranet.mpimp-golm.mpg.de/responder.py?name=fam!fam!Annexin) [ORTHO009375](http://aranet.mpimp-golm.mpg.de/responder.py?name=fam!fam!ORTHO009375) [HOM000320](http://aranet.mpimp-golm.mpg.de/responder.py?name=fam!fam!HOM000320) |
| [Bradi4g21240.1](http://aranet.mpimp-golm.mpg.de/responder.py?name=gene!Bdi!12045) | [bradi4g21240](http://aranet.mpimp-golm.mpg.de/responder.py?name=gene!Bdi!12045) | plant-specific domain TIGR01627 family protein, expressed | [DUF579](http://aranet.mpimp-golm.mpg.de/responder.py?name=fam!fam!DUF579) [HOM000646](http://aranet.mpimp-golm.mpg.de/responder.py?name=fam!fam!HOM000646) |
| [Bradi2g10302.1](http://aranet.mpimp-golm.mpg.de/responder.py?name=gene!Bdi!17160) | [bradi2g10302](http://aranet.mpimp-golm.mpg.de/responder.py?name=gene!Bdi!17160) | flavin monooxygenase, putative, expressed | [ORTHO019218](http://aranet.mpimp-golm.mpg.de/responder.py?name=fam!fam!ORTHO019218) [HOM000351](http://aranet.mpimp-golm.mpg.de/responder.py?name=fam!fam!HOM000351) |
| [Bradi2g17680.3](http://aranet.mpimp-golm.mpg.de/responder.py?name=gene!Bdi!7046) | [bradi2g17680](http://aranet.mpimp-golm.mpg.de/responder.py?name=gene!Bdi!7046) | CPuORF26 - conserved peptide uORF-containing transcript, expressed | [CMAS](http://aranet.mpimp-golm.mpg.de/responder.py?name=fam!fam!CMAS) [Hydroxy-O-Methy](http://aranet.mpimp-golm.mpg.de/responder.py?name=fam!fam!Hydroxy-O-Methy) [Methyltransf_11](http://aranet.mpimp-golm.mpg.de/responder.py?name=fam!fam!Methyltransf_11) [TehB](http://aranet.mpimp-golm.mpg.de/responder.py?name=fam!fam!TehB) [Ubie_methyltran](http://aranet.mpimp-golm.mpg.de/responder.py?name=fam!fam!Ubie_methyltran) [ORTHO001147](http://aranet.mpimp-golm.mpg.de/responder.py?name=fam!fam!ORTHO001147) [HOM002670](http://aranet.mpimp-golm.mpg.de/responder.py?name=fam!fam!HOM002670) |
| [Bradi3g56290.1](http://aranet.mpimp-golm.mpg.de/responder.py?name=gene!Bdi!11250) | [bradi3g56290](http://aranet.mpimp-golm.mpg.de/responder.py?name=gene!Bdi!11250) | bZIP transcription factor domain containing protein, expressed | [bZIP_1](http://aranet.mpimp-golm.mpg.de/responder.py?name=fam!fam!bZIP_1) [bZIP_2](http://aranet.mpimp-golm.mpg.de/responder.py?name=fam!fam!bZIP_2) [ORTHO007961](http://aranet.mpimp-golm.mpg.de/responder.py?name=fam!fam!ORTHO007961) [HOM000332](http://aranet.mpimp-golm.mpg.de/responder.py?name=fam!fam!HOM000332) |
| [Bradi5g16060.1](http://aranet.mpimp-golm.mpg.de/responder.py?name=gene!Bdi!8890) | [bradi5g16060](http://aranet.mpimp-golm.mpg.de/responder.py?name=gene!Bdi!8890) | expressed protein | [ORTHO023386](http://aranet.mpimp-golm.mpg.de/responder.py?name=fam!fam!ORTHO023386) [HOM001352](http://aranet.mpimp-golm.mpg.de/responder.py?name=fam!fam!HOM001352) |
| [Bradi5g02460.1](http://aranet.mpimp-golm.mpg.de/responder.py?name=gene!Bdi!21733) | [bradi5g02460](http://aranet.mpimp-golm.mpg.de/responder.py?name=gene!Bdi!21733) | cytochrome P450 93A2, putative, expressed | [ORTHO019696](http://aranet.mpimp-golm.mpg.de/responder.py?name=fam!fam!ORTHO019696) [HOM000005](http://aranet.mpimp-golm.mpg.de/responder.py?name=fam!fam!HOM000005) |
| [Bradi3g33070.1](http://aranet.mpimp-golm.mpg.de/responder.py?name=gene!Bdi!6131) | [bradi3g33070](http://aranet.mpimp-golm.mpg.de/responder.py?name=gene!Bdi!6131) | expressed protein | [DUF566](http://aranet.mpimp-golm.mpg.de/responder.py?name=fam!fam!DUF566) [ORTHO022740](http://aranet.mpimp-golm.mpg.de/responder.py?name=fam!fam!ORTHO022740) [HOM000545](http://aranet.mpimp-golm.mpg.de/responder.py?name=fam!fam!HOM000545) |
| [Bradi1g23620.1](http://aranet.mpimp-golm.mpg.de/responder.py?name=gene!Bdi!941) | [bradi1g23620](http://aranet.mpimp-golm.mpg.de/responder.py?name=gene!Bdi!941) | expressed protein | [ORTHO026141](http://aranet.mpimp-golm.mpg.de/responder.py?name=fam!fam!ORTHO026141) [HOM008361](http://aranet.mpimp-golm.mpg.de/responder.py?name=fam!fam!HOM008361) |
| [Bradi5g23460.1](http://aranet.mpimp-golm.mpg.de/responder.py?name=gene!Bdi!11397) | [bradi5g23460](http://aranet.mpimp-golm.mpg.de/responder.py?name=gene!Bdi!11397) | shikimate kinase, putative, expressed | [ORTHO001690](http://aranet.mpimp-golm.mpg.de/responder.py?name=fam!fam!ORTHO001690) [HOM001947](http://aranet.mpimp-golm.mpg.de/responder.py?name=fam!fam!HOM001947) |
| [Bradi2g46197.1](http://aranet.mpimp-golm.mpg.de/responder.py?name=gene!Bdi!7256) | [bradi2g46197](http://aranet.mpimp-golm.mpg.de/responder.py?name=gene!Bdi!7256) | no apical meristem protein, putative, expressed | [ORTHO008852](http://aranet.mpimp-golm.mpg.de/responder.py?name=fam!fam!ORTHO008852) [HOM000659](http://aranet.mpimp-golm.mpg.de/responder.py?name=fam!fam!HOM000659) |
| [Bradi2g12370.1](http://aranet.mpimp-golm.mpg.de/responder.py?name=gene!Bdi!17772) | [bradi2g12370](http://aranet.mpimp-golm.mpg.de/responder.py?name=gene!Bdi!17772) | GDSL-like lipase/acylhydrolase, putative, expressed | [Lipase_GDSL](http://aranet.mpimp-golm.mpg.de/responder.py?name=fam!fam!Lipase_GDSL) [ORTHO022049](http://aranet.mpimp-golm.mpg.de/responder.py?name=fam!fam!ORTHO022049) [HOM000086](http://aranet.mpimp-golm.mpg.de/responder.py?name=fam!fam!HOM000086) |
| [Bradi5g04540.1](http://aranet.mpimp-golm.mpg.de/responder.py?name=gene!Bdi!18435) | [bradi5g04540](http://aranet.mpimp-golm.mpg.de/responder.py?name=gene!Bdi!18435) | zinc finger, C3HC4 type domain containing protein, expressed | [ORTHO023311](http://aranet.mpimp-golm.mpg.de/responder.py?name=fam!fam!ORTHO023311) [HOM000242](http://aranet.mpimp-golm.mpg.de/responder.py?name=fam!fam!HOM000242) |
| [Bradi1g03940.1](http://aranet.mpimp-golm.mpg.de/responder.py?name=gene!Bdi!9452) | [bradi1g03940](http://aranet.mpimp-golm.mpg.de/responder.py?name=gene!Bdi!9452) | leaf senescence related protein, putative, expressed | [DUF231](http://aranet.mpimp-golm.mpg.de/responder.py?name=fam!fam!DUF231) [ORTHO006504](http://aranet.mpimp-golm.mpg.de/responder.py?name=fam!fam!ORTHO006504) [HOM000050](http://aranet.mpimp-golm.mpg.de/responder.py?name=fam!fam!HOM000050) |
| [Bradi2g23530.1](http://aranet.mpimp-golm.mpg.de/responder.py?name=gene!Bdi!18886) | [bradi2g23530](http://aranet.mpimp-golm.mpg.de/responder.py?name=gene!Bdi!18886) | homeodomain protein, putative, expressed | [Coprinus_mating](http://aranet.mpimp-golm.mpg.de/responder.py?name=fam!fam!Coprinus_mating) [Homeobox](http://aranet.mpimp-golm.mpg.de/responder.py?name=fam!fam!Homeobox) [POX](http://aranet.mpimp-golm.mpg.de/responder.py?name=fam!fam!POX) [ORTHO019268](http://aranet.mpimp-golm.mpg.de/responder.py?name=fam!fam!ORTHO019268) [HOM000277](http://aranet.mpimp-golm.mpg.de/responder.py?name=fam!fam!HOM000277) |
| [Bradi2g16337.1](http://aranet.mpimp-golm.mpg.de/responder.py?name=gene!Bdi!2845) | [bradi2g16337](http://aranet.mpimp-golm.mpg.de/responder.py?name=gene!Bdi!2845) | CGMC_MAPKCMGC_2.8 - CGMC includes CDA, MAPK, GSK3, and CLKC kinases, expressed | [ORTHO000061](http://aranet.mpimp-golm.mpg.de/responder.py?name=fam!fam!ORTHO000061) [HOM000019](http://aranet.mpimp-golm.mpg.de/responder.py?name=fam!fam!HOM000019) |
| [Bradi5g18150.1](http://aranet.mpimp-golm.mpg.de/responder.py?name=gene!Bdi!10299) | [bradi5g18150](http://aranet.mpimp-golm.mpg.de/responder.py?name=gene!Bdi!10299) | expressed protein | [ORTHO023405](http://aranet.mpimp-golm.mpg.de/responder.py?name=fam!fam!ORTHO023405) [HOM006997](http://aranet.mpimp-golm.mpg.de/responder.py?name=fam!fam!HOM006997) |
| [Bradi1g67460.1](http://aranet.mpimp-golm.mpg.de/responder.py?name=gene!Bdi!14918) | [bradi1g67460](http://aranet.mpimp-golm.mpg.de/responder.py?name=gene!Bdi!14918) | phospholipase A2, putative, expressed | [Phospholip_A2_1](http://aranet.mpimp-golm.mpg.de/responder.py?name=fam!fam!Phospholip_A2_1) [HOM000828](http://aranet.mpimp-golm.mpg.de/responder.py?name=fam!fam!HOM000828) |
| [Bradi3g42430.1](http://aranet.mpimp-golm.mpg.de/responder.py?name=gene!Bdi!18494) | [bradi3g42430](http://aranet.mpimp-golm.mpg.de/responder.py?name=gene!Bdi!18494) | MYB family transcription factor, putative, expressed | [Actin](http://aranet.mpimp-golm.mpg.de/responder.py?name=fam!fam!Actin) [Myb_DNA-binding](http://aranet.mpimp-golm.mpg.de/responder.py?name=fam!fam!Myb_DNA-binding) [ORTHO014260](http://aranet.mpimp-golm.mpg.de/responder.py?name=fam!fam!ORTHO014260) [HOM000007](http://aranet.mpimp-golm.mpg.de/responder.py?name=fam!fam!HOM000007) |
| [Bradi3g41210.1](http://aranet.mpimp-golm.mpg.de/responder.py?name=gene!Bdi!4481) | [bradi3g41210](http://aranet.mpimp-golm.mpg.de/responder.py?name=gene!Bdi!4481) | microtubule associated protein, putative, expressed | [MAP65_ASE1](http://aranet.mpimp-golm.mpg.de/responder.py?name=fam!fam!MAP65_ASE1) [ORTHO010611](http://aranet.mpimp-golm.mpg.de/responder.py?name=fam!fam!ORTHO010611) [HOM000492](http://aranet.mpimp-golm.mpg.de/responder.py?name=fam!fam!HOM000492) |
| [Bradi2g17067.1](http://aranet.mpimp-golm.mpg.de/responder.py?name=gene!Bdi!4637) | [bradi2g17067](http://aranet.mpimp-golm.mpg.de/responder.py?name=gene!Bdi!4637) | auxin-responsive protein, putative, expressed | [ORTHO022083](http://aranet.mpimp-golm.mpg.de/responder.py?name=fam!fam!ORTHO022083) [HOM000285](http://aranet.mpimp-golm.mpg.de/responder.py?name=fam!fam!HOM000285) |
| [Bradi4g40400.1](http://aranet.mpimp-golm.mpg.de/responder.py?name=gene!Bdi!19953) | [bradi4g40400](http://aranet.mpimp-golm.mpg.de/responder.py?name=gene!Bdi!19953) | plant-specific domain TIGR01627 family protein, expressed | [ORTHO007686](http://aranet.mpimp-golm.mpg.de/responder.py?name=fam!fam!ORTHO007686) [HOM000646](http://aranet.mpimp-golm.mpg.de/responder.py?name=fam!fam!HOM000646) |
| [Bradi4g37980.1](http://aranet.mpimp-golm.mpg.de/responder.py?name=gene!Bdi!3143) | [bradi4g37980](http://aranet.mpimp-golm.mpg.de/responder.py?name=gene!Bdi!3143) | cell cycle control protein, putative, expressed | [ORTHO026939](http://aranet.mpimp-golm.mpg.de/responder.py?name=fam!fam!ORTHO026939) [HOM000848](http://aranet.mpimp-golm.mpg.de/responder.py?name=fam!fam!HOM000848) |
| [Bradi1g10660.1](http://aranet.mpimp-golm.mpg.de/responder.py?name=gene!Bdi!8948) | [bradi1g10660](http://aranet.mpimp-golm.mpg.de/responder.py?name=gene!Bdi!8948) | histidine kinase, putative, expressed | [CHASE](http://aranet.mpimp-golm.mpg.de/responder.py?name=fam!fam!CHASE) [HATPase_c](http://aranet.mpimp-golm.mpg.de/responder.py?name=fam!fam!HATPase_c) [HisKA](http://aranet.mpimp-golm.mpg.de/responder.py?name=fam!fam!HisKA) [Response_reg](http://aranet.mpimp-golm.mpg.de/responder.py?name=fam!fam!Response_reg) [ORTHO000236](http://aranet.mpimp-golm.mpg.de/responder.py?name=fam!fam!ORTHO000236) [HOM000299](http://aranet.mpimp-golm.mpg.de/responder.py?name=fam!fam!HOM000299) |
| [Bradi3g40820.1](http://aranet.mpimp-golm.mpg.de/responder.py?name=gene!Bdi!12679) | [bradi3g40820](http://aranet.mpimp-golm.mpg.de/responder.py?name=gene!Bdi!12679) | membrane protein, putative, expressed | [Cytochrom_B561](http://aranet.mpimp-golm.mpg.de/responder.py?name=fam!fam!Cytochrom_B561) [Cytochrom_C_asm](http://aranet.mpimp-golm.mpg.de/responder.py?name=fam!fam!Cytochrom_C_asm) [DUF568](http://aranet.mpimp-golm.mpg.de/responder.py?name=fam!fam!DUF568) [ORTHO003253](http://aranet.mpimp-golm.mpg.de/responder.py?name=fam!fam!ORTHO003253) [HOM000285](http://aranet.mpimp-golm.mpg.de/responder.py?name=fam!fam!HOM000285) |
| [Bradi4g16560.1](http://aranet.mpimp-golm.mpg.de/responder.py?name=gene!Bdi!16843) | [bradi4g16560](http://aranet.mpimp-golm.mpg.de/responder.py?name=gene!Bdi!16843) | cytochrome P450, putative, expressed | [p450](http://aranet.mpimp-golm.mpg.de/responder.py?name=fam!fam!p450) [ORTHO019002](http://aranet.mpimp-golm.mpg.de/responder.py?name=fam!fam!ORTHO019002) [HOM000005](http://aranet.mpimp-golm.mpg.de/responder.py?name=fam!fam!HOM000005) |
| [Bradi1g00710.1](http://aranet.mpimp-golm.mpg.de/responder.py?name=gene!Bdi!13630) | [bradi1g00710](http://aranet.mpimp-golm.mpg.de/responder.py?name=gene!Bdi!13630) | expressed protein | [DUF547](http://aranet.mpimp-golm.mpg.de/responder.py?name=fam!fam!DUF547) [ORTHO010994](http://aranet.mpimp-golm.mpg.de/responder.py?name=fam!fam!ORTHO010994) [HOM000340](http://aranet.mpimp-golm.mpg.de/responder.py?name=fam!fam!HOM000340) |
| [Bradi2g00880.1](http://aranet.mpimp-golm.mpg.de/responder.py?name=gene!Bdi!21421) | [bradi2g00880](http://aranet.mpimp-golm.mpg.de/responder.py?name=gene!Bdi!21421) | No annotation | [HOM029547](http://aranet.mpimp-golm.mpg.de/responder.py?name=fam!fam!HOM029547) |
| [Bradi1g65530.1](http://aranet.mpimp-golm.mpg.de/responder.py?name=gene!Bdi!13711) | [bradi1g65530](http://aranet.mpimp-golm.mpg.de/responder.py?name=gene!Bdi!13711) | expressed protein | [DUF231](http://aranet.mpimp-golm.mpg.de/responder.py?name=fam!fam!DUF231) [ORTHO005860](http://aranet.mpimp-golm.mpg.de/responder.py?name=fam!fam!ORTHO005860) [HOM000050](http://aranet.mpimp-golm.mpg.de/responder.py?name=fam!fam!HOM000050) |
| [Bradi3g32180.1](http://aranet.mpimp-golm.mpg.de/responder.py?name=gene!Bdi!15474) | [bradi3g32180](http://aranet.mpimp-golm.mpg.de/responder.py?name=gene!Bdi!15474) | expressed protein | [ORTHO004958](http://aranet.mpimp-golm.mpg.de/responder.py?name=fam!fam!ORTHO004958) [HOM003925](http://aranet.mpimp-golm.mpg.de/responder.py?name=fam!fam!HOM003925) |
| [Bradi4g30540.1](http://aranet.mpimp-golm.mpg.de/responder.py?name=gene!Bdi!13203) | [bradi4g30540](http://aranet.mpimp-golm.mpg.de/responder.py?name=gene!Bdi!13203) | CESA9 - cellulose synthase, expressed | [Cellulose_synt](http://aranet.mpimp-golm.mpg.de/responder.py?name=fam!fam!Cellulose_synt) [Glycos_transf_2](http://aranet.mpimp-golm.mpg.de/responder.py?name=fam!fam!Glycos_transf_2) [ORTHO000003](http://aranet.mpimp-golm.mpg.de/responder.py?name=fam!fam!ORTHO000003) [HOM000082](http://aranet.mpimp-golm.mpg.de/responder.py?name=fam!fam!HOM000082) |
| [Bradi2g24010.1](http://aranet.mpimp-golm.mpg.de/responder.py?name=gene!Bdi!11662) | [bradi2g24010](http://aranet.mpimp-golm.mpg.de/responder.py?name=gene!Bdi!11662) | protein kinase, putative, expressed | [Pkinase](http://aranet.mpimp-golm.mpg.de/responder.py?name=fam!fam!Pkinase) [ORTHO004482](http://aranet.mpimp-golm.mpg.de/responder.py?name=fam!fam!ORTHO004482) [HOM000651](http://aranet.mpimp-golm.mpg.de/responder.py?name=fam!fam!HOM000651) |
| [Bradi4g33490.1](http://aranet.mpimp-golm.mpg.de/responder.py?name=gene!Bdi!11026) | [bradi4g33490](http://aranet.mpimp-golm.mpg.de/responder.py?name=gene!Bdi!11026) | fasciclin domain containing protein, expressed | [Fasciclin](http://aranet.mpimp-golm.mpg.de/responder.py?name=fam!fam!Fasciclin) [ORTHO010041](http://aranet.mpimp-golm.mpg.de/responder.py?name=fam!fam!ORTHO010041) [HOM000419](http://aranet.mpimp-golm.mpg.de/responder.py?name=fam!fam!HOM000419) |
| [**Bradi2g23370.1**](http://aranet.mpimp-golm.mpg.de/responder.py?name=gene!Bdi!18168) | [**bradi2g23370**](http://aranet.mpimp-golm.mpg.de/responder.py?name=gene!Bdi!18168) | **laccase precursor protein, putative, expressed** | [**Cu-oxidase**](http://aranet.mpimp-golm.mpg.de/responder.py?name=fam!fam!Cu-oxidase) [**Cu-oxidase_2**](http://aranet.mpimp-golm.mpg.de/responder.py?name=fam!fam!Cu-oxidase_2) [**Cu-oxidase_3**](http://aranet.mpimp-golm.mpg.de/responder.py?name=fam!fam!Cu-oxidase_3) [**ORTHO000023**](http://aranet.mpimp-golm.mpg.de/responder.py?name=fam!fam!ORTHO000023) [**HOM000096**](http://aranet.mpimp-golm.mpg.de/responder.py?name=fam!fam!HOM000096) |
| [Bradi1g34670.1](http://aranet.mpimp-golm.mpg.de/responder.py?name=gene!Bdi!6994) | [bradi1g34670](http://aranet.mpimp-golm.mpg.de/responder.py?name=gene!Bdi!6994) | glycosyltransferase, putative, expressed | [DUF563](http://aranet.mpimp-golm.mpg.de/responder.py?name=fam!fam!DUF563) [ORTHO021591](http://aranet.mpimp-golm.mpg.de/responder.py?name=fam!fam!ORTHO021591) [HOM000406](http://aranet.mpimp-golm.mpg.de/responder.py?name=fam!fam!HOM000406) |
| [Bradi3g39800.1](http://aranet.mpimp-golm.mpg.de/responder.py?name=gene!Bdi!750) | [bradi3g39800](http://aranet.mpimp-golm.mpg.de/responder.py?name=gene!Bdi!750) | citrate transporter, putative, expressed | [CitMHS](http://aranet.mpimp-golm.mpg.de/responder.py?name=fam!fam!CitMHS) [Na_sulph_symp](http://aranet.mpimp-golm.mpg.de/responder.py?name=fam!fam!Na_sulph_symp) [ORTHO003625](http://aranet.mpimp-golm.mpg.de/responder.py?name=fam!fam!ORTHO003625) [HOM002918](http://aranet.mpimp-golm.mpg.de/responder.py?name=fam!fam!HOM002918) |
| [Bradi1g31820.1](http://aranet.mpimp-golm.mpg.de/responder.py?name=gene!Bdi!8373) | [bradi1g31820](http://aranet.mpimp-golm.mpg.de/responder.py?name=gene!Bdi!8373) | vacuolar-sorting receptor precursor, putative, expressed | [EGF_CA](http://aranet.mpimp-golm.mpg.de/responder.py?name=fam!fam!EGF_CA) [PA](http://aranet.mpimp-golm.mpg.de/responder.py?name=fam!fam!PA) [Peptidase_M36](http://aranet.mpimp-golm.mpg.de/responder.py?name=fam!fam!Peptidase_M36) [ORTHO000113](http://aranet.mpimp-golm.mpg.de/responder.py?name=fam!fam!ORTHO000113) [HOM000457](http://aranet.mpimp-golm.mpg.de/responder.py?name=fam!fam!HOM000457) |
| [Bradi1g54420.1](http://aranet.mpimp-golm.mpg.de/responder.py?name=gene!Bdi!21657) | [bradi1g54420](http://aranet.mpimp-golm.mpg.de/responder.py?name=gene!Bdi!21657) | expressed protein | [HOM000834](http://aranet.mpimp-golm.mpg.de/responder.py?name=fam!fam!HOM000834) |
| [Bradi1g17242.1](http://aranet.mpimp-golm.mpg.de/responder.py?name=gene!Bdi!10526) | [bradi1g17242](http://aranet.mpimp-golm.mpg.de/responder.py?name=gene!Bdi!10526) | serine hydrolase domain containing protein, expressed | [ORTHO012168](http://aranet.mpimp-golm.mpg.de/responder.py?name=fam!fam!ORTHO012168) [HOM001917](http://aranet.mpimp-golm.mpg.de/responder.py?name=fam!fam!HOM001917) |
| [Bradi1g74320.1](http://aranet.mpimp-golm.mpg.de/responder.py?name=gene!Bdi!21548) | [bradi1g74320](http://aranet.mpimp-golm.mpg.de/responder.py?name=gene!Bdi!21548) | laccase-22 precursor, putative, expressed | [Cu-oxidase](http://aranet.mpimp-golm.mpg.de/responder.py?name=fam!fam!Cu-oxidase) [Cu-oxidase_2](http://aranet.mpimp-golm.mpg.de/responder.py?name=fam!fam!Cu-oxidase_2) [Cu-oxidase_3](http://aranet.mpimp-golm.mpg.de/responder.py?name=fam!fam!Cu-oxidase_3) [ORTHO000023](http://aranet.mpimp-golm.mpg.de/responder.py?name=fam!fam!ORTHO000023) [HOM000096](http://aranet.mpimp-golm.mpg.de/responder.py?name=fam!fam!HOM000096) |
| [Bradi4g05450.1](http://aranet.mpimp-golm.mpg.de/responder.py?name=gene!Bdi!21497) | [bradi4g05450](http://aranet.mpimp-golm.mpg.de/responder.py?name=gene!Bdi!21497) | isoflavone reductase homolog IRL, putative, expressed | [Epimerase](http://aranet.mpimp-golm.mpg.de/responder.py?name=fam!fam!Epimerase) [NmrA](http://aranet.mpimp-golm.mpg.de/responder.py?name=fam!fam!NmrA) [ORTHO004598](http://aranet.mpimp-golm.mpg.de/responder.py?name=fam!fam!ORTHO004598) [HOM000346](http://aranet.mpimp-golm.mpg.de/responder.py?name=fam!fam!HOM000346) |
| [Bradi3g00377.1](http://aranet.mpimp-golm.mpg.de/responder.py?name=gene!Bdi!21364) | [bradi3g00377](http://aranet.mpimp-golm.mpg.de/responder.py?name=gene!Bdi!21364) | rhodanese-like domain containing protein, putative, expressed | [ORTHO013010](http://aranet.mpimp-golm.mpg.de/responder.py?name=fam!fam!ORTHO013010) [HOM001275](http://aranet.mpimp-golm.mpg.de/responder.py?name=fam!fam!HOM001275) |
| [Bradi2g37970.1](http://aranet.mpimp-golm.mpg.de/responder.py?name=gene!Bdi!3316) | [bradi2g37970](http://aranet.mpimp-golm.mpg.de/responder.py?name=gene!Bdi!3316) | glycosyltransferase family 43 protein, putative, expressed | [Glyco_transf_43](http://aranet.mpimp-golm.mpg.de/responder.py?name=fam!fam!Glyco_transf_43) [ORTHO003069](http://aranet.mpimp-golm.mpg.de/responder.py?name=fam!fam!ORTHO003069) [HOM001149](http://aranet.mpimp-golm.mpg.de/responder.py?name=fam!fam!HOM001149) |
| [Bradi3g59730.1](http://aranet.mpimp-golm.mpg.de/responder.py?name=gene!Bdi!1887) | [bradi3g59730](http://aranet.mpimp-golm.mpg.de/responder.py?name=gene!Bdi!1887) | HEAT repeat family protein, putative, expressed | [Adaptin_N](http://aranet.mpimp-golm.mpg.de/responder.py?name=fam!fam!Adaptin_N) [ORTHO007509](http://aranet.mpimp-golm.mpg.de/responder.py?name=fam!fam!ORTHO007509) [HOM000971](http://aranet.mpimp-golm.mpg.de/responder.py?name=fam!fam!HOM000971) |
| [Bradi4g31130.1](http://aranet.mpimp-golm.mpg.de/responder.py?name=gene!Bdi!14462) | [bradi4g31130](http://aranet.mpimp-golm.mpg.de/responder.py?name=gene!Bdi!14462) | ferric reductase, putative, expressed | [FAD_binding_8](http://aranet.mpimp-golm.mpg.de/responder.py?name=fam!fam!FAD_binding_8) [Ferric_reduct](http://aranet.mpimp-golm.mpg.de/responder.py?name=fam!fam!Ferric_reduct) [NADPH_Ox](http://aranet.mpimp-golm.mpg.de/responder.py?name=fam!fam!NADPH_Ox) [NAD_binding_6](http://aranet.mpimp-golm.mpg.de/responder.py?name=fam!fam!NAD_binding_6) [ORTHO000035](http://aranet.mpimp-golm.mpg.de/responder.py?name=fam!fam!ORTHO000035) [HOM000327](http://aranet.mpimp-golm.mpg.de/responder.py?name=fam!fam!HOM000327) |
| [Bradi1g17830.1](http://aranet.mpimp-golm.mpg.de/responder.py?name=gene!Bdi!10165) | [bradi1g17830](http://aranet.mpimp-golm.mpg.de/responder.py?name=gene!Bdi!10165) | potassium transporter, putative, expressed | [K_trans](http://aranet.mpimp-golm.mpg.de/responder.py?name=fam!fam!K_trans) [ORTHO000022](http://aranet.mpimp-golm.mpg.de/responder.py?name=fam!fam!ORTHO000022) [HOM000120](http://aranet.mpimp-golm.mpg.de/responder.py?name=fam!fam!HOM000120) |
| [Bradi1g25117.1](http://aranet.mpimp-golm.mpg.de/responder.py?name=gene!Bdi!11855) | [bradi1g25117](http://aranet.mpimp-golm.mpg.de/responder.py?name=gene!Bdi!11855) | CSLF2 - cellulose synthase-like family F; beta1,3;1,4 glucan synthase, expressed | [ORTHO000003](http://aranet.mpimp-golm.mpg.de/responder.py?name=fam!fam!ORTHO000003) [HOM000082](http://aranet.mpimp-golm.mpg.de/responder.py?name=fam!fam!HOM000082) |
| [Bradi4g06680.1](http://aranet.mpimp-golm.mpg.de/responder.py?name=gene!Bdi!19066) | [bradi4g06680](http://aranet.mpimp-golm.mpg.de/responder.py?name=gene!Bdi!19066) | expressed protein | [ORTHO023058](http://aranet.mpimp-golm.mpg.de/responder.py?name=fam!fam!ORTHO023058) [HOM003871](http://aranet.mpimp-golm.mpg.de/responder.py?name=fam!fam!HOM003871) |
| [Bradi1g09460.1](http://aranet.mpimp-golm.mpg.de/responder.py?name=gene!Bdi!9292) | [bradi1g09460](http://aranet.mpimp-golm.mpg.de/responder.py?name=gene!Bdi!9292) | endoglucanase, putative, expressed | [Glyco_hydro_9](http://aranet.mpimp-golm.mpg.de/responder.py?name=fam!fam!Glyco_hydro_9) [ORTHO000460](http://aranet.mpimp-golm.mpg.de/responder.py?name=fam!fam!ORTHO000460) [HOM000137](http://aranet.mpimp-golm.mpg.de/responder.py?name=fam!fam!HOM000137) |
| [Bradi4g27720.1](http://aranet.mpimp-golm.mpg.de/responder.py?name=gene!Bdi!6010) | [bradi4g27720](http://aranet.mpimp-golm.mpg.de/responder.py?name=gene!Bdi!6010) | expressed protein | [bZIP_1](http://aranet.mpimp-golm.mpg.de/responder.py?name=fam!fam!bZIP_1) [bZIP_2](http://aranet.mpimp-golm.mpg.de/responder.py?name=fam!fam!bZIP_2) [ORTHO019634](http://aranet.mpimp-golm.mpg.de/responder.py?name=fam!fam!ORTHO019634) [HOM000332](http://aranet.mpimp-golm.mpg.de/responder.py?name=fam!fam!HOM000332) |
| [Bradi1g64560.1](http://aranet.mpimp-golm.mpg.de/responder.py?name=gene!Bdi!17875) | [bradi1g64560](http://aranet.mpimp-golm.mpg.de/responder.py?name=gene!Bdi!17875) | glycosyltransferase, putative, expressed | [Glyco_transf_34](http://aranet.mpimp-golm.mpg.de/responder.py?name=fam!fam!Glyco_transf_34) [ORTHO026314](http://aranet.mpimp-golm.mpg.de/responder.py?name=fam!fam!ORTHO026314) [HOM000584](http://aranet.mpimp-golm.mpg.de/responder.py?name=fam!fam!HOM000584) |
| [Bradi5g08907.1](http://aranet.mpimp-golm.mpg.de/responder.py?name=gene!Bdi!23024) | [bradi5g08907](http://aranet.mpimp-golm.mpg.de/responder.py?name=gene!Bdi!23024) | heparan-alpha-glucosaminide N-acetyltransferase, putative, expressed | [ORTHO004670](http://aranet.mpimp-golm.mpg.de/responder.py?name=fam!fam!ORTHO004670) [HOM000992](http://aranet.mpimp-golm.mpg.de/responder.py?name=fam!fam!HOM000992) |
| [Bradi2g16560.1](http://aranet.mpimp-golm.mpg.de/responder.py?name=gene!Bdi!18410) | [bradi2g16560](http://aranet.mpimp-golm.mpg.de/responder.py?name=gene!Bdi!18410) | fasciclin domain containing protein, expressed | [Fasciclin](http://aranet.mpimp-golm.mpg.de/responder.py?name=fam!fam!Fasciclin) [ORTHO026432](http://aranet.mpimp-golm.mpg.de/responder.py?name=fam!fam!ORTHO026432) [HOM000419](http://aranet.mpimp-golm.mpg.de/responder.py?name=fam!fam!HOM000419) |
| [Bradi3g16530.1](http://aranet.mpimp-golm.mpg.de/responder.py?name=gene!Bdi!13493) | [bradi3g16530](http://aranet.mpimp-golm.mpg.de/responder.py?name=gene!Bdi!13493) | O-methyltransferase, putative, expressed | [Dimerisation](http://aranet.mpimp-golm.mpg.de/responder.py?name=fam!fam!Dimerisation) [Methyltransf_2](http://aranet.mpimp-golm.mpg.de/responder.py?name=fam!fam!Methyltransf_2) [ORTHO002068](http://aranet.mpimp-golm.mpg.de/responder.py?name=fam!fam!ORTHO002068) [HOM000097](http://aranet.mpimp-golm.mpg.de/responder.py?name=fam!fam!HOM000097) |
| [Bradi2g54680.1](http://aranet.mpimp-golm.mpg.de/responder.py?name=gene!Bdi!539) | [bradi2g54680](http://aranet.mpimp-golm.mpg.de/responder.py?name=gene!Bdi!539) | laccase precursor protein, putative, expressed | [Cu-oxidase](http://aranet.mpimp-golm.mpg.de/responder.py?name=fam!fam!Cu-oxidase) [Cu-oxidase_2](http://aranet.mpimp-golm.mpg.de/responder.py?name=fam!fam!Cu-oxidase_2) [Cu-oxidase_3](http://aranet.mpimp-golm.mpg.de/responder.py?name=fam!fam!Cu-oxidase_3) [ORTHO000023](http://aranet.mpimp-golm.mpg.de/responder.py?name=fam!fam!ORTHO000023) [HOM000096](http://aranet.mpimp-golm.mpg.de/responder.py?name=fam!fam!HOM000096) |
| [Bradi4g23910.1](http://aranet.mpimp-golm.mpg.de/responder.py?name=gene!Bdi!23082) | [bradi4g23910](http://aranet.mpimp-golm.mpg.de/responder.py?name=gene!Bdi!23082) | KIP1, putative, expressed | [KIP1](http://aranet.mpimp-golm.mpg.de/responder.py?name=fam!fam!KIP1) [ORTHO014781](http://aranet.mpimp-golm.mpg.de/responder.py?name=fam!fam!ORTHO014781) [HOM000841](http://aranet.mpimp-golm.mpg.de/responder.py?name=fam!fam!HOM000841) |
| [Bradi1g06290.1](http://aranet.mpimp-golm.mpg.de/responder.py?name=gene!Bdi!1309) | [bradi1g06290](http://aranet.mpimp-golm.mpg.de/responder.py?name=gene!Bdi!1309) | fasciclin domain containing protein, expressed | [Fasciclin](http://aranet.mpimp-golm.mpg.de/responder.py?name=fam!fam!Fasciclin) [ORTHO003396](http://aranet.mpimp-golm.mpg.de/responder.py?name=fam!fam!ORTHO003396) [HOM002140](http://aranet.mpimp-golm.mpg.de/responder.py?name=fam!fam!HOM002140) |
| [Bradi5g14720.1](http://aranet.mpimp-golm.mpg.de/responder.py?name=gene!Bdi!17028) | [bradi5g14720](http://aranet.mpimp-golm.mpg.de/responder.py?name=gene!Bdi!17028) | transferase family protein, putative, expressed | [ORTHO001336](http://aranet.mpimp-golm.mpg.de/responder.py?name=fam!fam!ORTHO001336) [HOM000062](http://aranet.mpimp-golm.mpg.de/responder.py?name=fam!fam!HOM000062) |
| [Bradi1g35477.1](http://aranet.mpimp-golm.mpg.de/responder.py?name=gene!Bdi!662) | [bradi1g35477](http://aranet.mpimp-golm.mpg.de/responder.py?name=gene!Bdi!662) | STRUBBELIG-RECEPTOR FAMILY 7 precursor, putative, expressed | [ORTHO002829](http://aranet.mpimp-golm.mpg.de/responder.py?name=fam!fam!ORTHO002829) [HOM000004](http://aranet.mpimp-golm.mpg.de/responder.py?name=fam!fam!HOM000004) |
| [Bradi2g47590.1](http://aranet.mpimp-golm.mpg.de/responder.py?name=gene!Bdi!9070) | [bradi2g47590](http://aranet.mpimp-golm.mpg.de/responder.py?name=gene!Bdi!9070) | MYB family transcription factor, putative, expressed | [Actin](http://aranet.mpimp-golm.mpg.de/responder.py?name=fam!fam!Actin) [Myb_DNA-binding](http://aranet.mpimp-golm.mpg.de/responder.py?name=fam!fam!Myb_DNA-binding) [ORTHO022335](http://aranet.mpimp-golm.mpg.de/responder.py?name=fam!fam!ORTHO022335) [HOM000007](http://aranet.mpimp-golm.mpg.de/responder.py?name=fam!fam!HOM000007) |
| [Bradi3g58560.1](http://aranet.mpimp-golm.mpg.de/responder.py?name=gene!Bdi!18429) | [bradi3g58560](http://aranet.mpimp-golm.mpg.de/responder.py?name=gene!Bdi!18429) | plastocyanin-like domain containing protein, putative, expressed | [Cu_bind_like](http://aranet.mpimp-golm.mpg.de/responder.py?name=fam!fam!Cu_bind_like) [ORTHO032695](http://aranet.mpimp-golm.mpg.de/responder.py?name=fam!fam!ORTHO032695) [HOM000055](http://aranet.mpimp-golm.mpg.de/responder.py?name=fam!fam!HOM000055) |
| [Bradi4g21220.1](http://aranet.mpimp-golm.mpg.de/responder.py?name=gene!Bdi!4367) | [bradi4g21220](http://aranet.mpimp-golm.mpg.de/responder.py?name=gene!Bdi!4367) | auxin-induced protein 5NG4, putative, expressed | [EamA](http://aranet.mpimp-golm.mpg.de/responder.py?name=fam!fam!EamA) [ORTHO003362](http://aranet.mpimp-golm.mpg.de/responder.py?name=fam!fam!ORTHO003362) [HOM000059](http://aranet.mpimp-golm.mpg.de/responder.py?name=fam!fam!HOM000059) |
| [Bradi2g19160.1](http://aranet.mpimp-golm.mpg.de/responder.py?name=gene!Bdi!16701) | [bradi2g19160](http://aranet.mpimp-golm.mpg.de/responder.py?name=gene!Bdi!16701) | anthocyanidin 5,3-O-glucosyltransferase, putative, expressed | [UDPGT](http://aranet.mpimp-golm.mpg.de/responder.py?name=fam!fam!UDPGT) [ORTHO014757](http://aranet.mpimp-golm.mpg.de/responder.py?name=fam!fam!ORTHO014757) [HOM000016](http://aranet.mpimp-golm.mpg.de/responder.py?name=fam!fam!HOM000016) |
| [Bradi3g28350.1](http://aranet.mpimp-golm.mpg.de/responder.py?name=gene!Bdi!14598) | [bradi3g28350](http://aranet.mpimp-golm.mpg.de/responder.py?name=gene!Bdi!14598) | CESA7 - cellulose synthase, expressed | [Cellulose_synt](http://aranet.mpimp-golm.mpg.de/responder.py?name=fam!fam!Cellulose_synt) [Glycos_transf_2](http://aranet.mpimp-golm.mpg.de/responder.py?name=fam!fam!Glycos_transf_2) [ORTHO000003](http://aranet.mpimp-golm.mpg.de/responder.py?name=fam!fam!ORTHO000003) [HOM000082](http://aranet.mpimp-golm.mpg.de/responder.py?name=fam!fam!HOM000082) |
| [Bradi3g48730.1](http://aranet.mpimp-golm.mpg.de/responder.py?name=gene!Bdi!3694) | [bradi3g48730](http://aranet.mpimp-golm.mpg.de/responder.py?name=gene!Bdi!3694) | plant protein of unknown function DUF869 domain containing protein, expressed | [DUF869](http://aranet.mpimp-golm.mpg.de/responder.py?name=fam!fam!DUF869) [ORTHO022886](http://aranet.mpimp-golm.mpg.de/responder.py?name=fam!fam!ORTHO022886) [HOM000578](http://aranet.mpimp-golm.mpg.de/responder.py?name=fam!fam!HOM000578) |
| [Bradi2g55340.1](http://aranet.mpimp-golm.mpg.de/responder.py?name=gene!Bdi!6663) | [bradi2g55340](http://aranet.mpimp-golm.mpg.de/responder.py?name=gene!Bdi!6663) | transmembrane amino acid transporter protein, putative, expressed | [Aa_trans](http://aranet.mpimp-golm.mpg.de/responder.py?name=fam!fam!Aa_trans) [ORTHO000128](http://aranet.mpimp-golm.mpg.de/responder.py?name=fam!fam!ORTHO000128) [HOM000823](http://aranet.mpimp-golm.mpg.de/responder.py?name=fam!fam!HOM000823) |
| [Bradi4g36240.1](http://aranet.mpimp-golm.mpg.de/responder.py?name=gene!Bdi!12250) | [bradi4g36240](http://aranet.mpimp-golm.mpg.de/responder.py?name=gene!Bdi!12250) | endoglucanase, putative, expressed | [Glyco_hydro_9](http://aranet.mpimp-golm.mpg.de/responder.py?name=fam!fam!Glyco_hydro_9) [ORTHO001222](http://aranet.mpimp-golm.mpg.de/responder.py?name=fam!fam!ORTHO001222) [HOM000137](http://aranet.mpimp-golm.mpg.de/responder.py?name=fam!fam!HOM000137) |
| [Bradi1g60750.1](http://aranet.mpimp-golm.mpg.de/responder.py?name=gene!Bdi!2308) | [bradi1g60750](http://aranet.mpimp-golm.mpg.de/responder.py?name=gene!Bdi!2308) | phospho-2-dehydro-3-deoxyheptonate aldolase, chloroplast precursor, putative, expressed | [DAHP_synth_2](http://aranet.mpimp-golm.mpg.de/responder.py?name=fam!fam!DAHP_synth_2) [ORTHO000246](http://aranet.mpimp-golm.mpg.de/responder.py?name=fam!fam!ORTHO000246) [HOM000967](http://aranet.mpimp-golm.mpg.de/responder.py?name=fam!fam!HOM000967) |
| [Bradi2g11010.1](http://aranet.mpimp-golm.mpg.de/responder.py?name=gene!Bdi!16334) | [bradi2g11010](http://aranet.mpimp-golm.mpg.de/responder.py?name=gene!Bdi!16334) | TsetseEP precursor, putative, expressed | [HOM043442](http://aranet.mpimp-golm.mpg.de/responder.py?name=fam!fam!HOM043442) |
| [Bradi4g25540.1](http://aranet.mpimp-golm.mpg.de/responder.py?name=gene!Bdi!12313) | [bradi4g25540](http://aranet.mpimp-golm.mpg.de/responder.py?name=gene!Bdi!12313) | BTB9 - Bric-a-Brac, Tramtrack, Broad Complex BTB domain, expressed | [BTB](http://aranet.mpimp-golm.mpg.de/responder.py?name=fam!fam!BTB) [ORTHO007279](http://aranet.mpimp-golm.mpg.de/responder.py?name=fam!fam!ORTHO007279) [HOM002599](http://aranet.mpimp-golm.mpg.de/responder.py?name=fam!fam!HOM002599) |
| [Bradi5g09130.1](http://aranet.mpimp-golm.mpg.de/responder.py?name=gene!Bdi!17092) | [bradi5g09130](http://aranet.mpimp-golm.mpg.de/responder.py?name=gene!Bdi!17092) | expressed protein | [ORTHO008594](http://aranet.mpimp-golm.mpg.de/responder.py?name=fam!fam!ORTHO008594) [HOM001781](http://aranet.mpimp-golm.mpg.de/responder.py?name=fam!fam!HOM001781) |
| [Bradi4g28260.1](http://aranet.mpimp-golm.mpg.de/responder.py?name=gene!Bdi!9938) | [bradi4g28260](http://aranet.mpimp-golm.mpg.de/responder.py?name=gene!Bdi!9938) | hydroxyproline-rich glycoprotein family protein, putative, expressed | [HOM089020](http://aranet.mpimp-golm.mpg.de/responder.py?name=fam!fam!HOM089020) |
| [Bradi5g12460.1](http://aranet.mpimp-golm.mpg.de/responder.py?name=gene!Bdi!17544) | [bradi5g12460](http://aranet.mpimp-golm.mpg.de/responder.py?name=gene!Bdi!17544) | uncharacterized Cys-rich domain containing protein, putative, expressed | [ORTHO001933](http://aranet.mpimp-golm.mpg.de/responder.py?name=fam!fam!ORTHO001933) [HOM000387](http://aranet.mpimp-golm.mpg.de/responder.py?name=fam!fam!HOM000387) |
| [Bradi3g06480.1](http://aranet.mpimp-golm.mpg.de/responder.py?name=gene!Bdi!21912) | [bradi3g06480](http://aranet.mpimp-golm.mpg.de/responder.py?name=gene!Bdi!21912) | dehydrogenase, putative, expressed | [ADH_N](http://aranet.mpimp-golm.mpg.de/responder.py?name=fam!fam!ADH_N) [ADH_zinc_N](http://aranet.mpimp-golm.mpg.de/responder.py?name=fam!fam!ADH_zinc_N) [ORTHO009302](http://aranet.mpimp-golm.mpg.de/responder.py?name=fam!fam!ORTHO009302) [HOM000222](http://aranet.mpimp-golm.mpg.de/responder.py?name=fam!fam!HOM000222) |
| [Bradi1g57607.1](http://aranet.mpimp-golm.mpg.de/responder.py?name=gene!Bdi!2818) | [bradi1g57607](http://aranet.mpimp-golm.mpg.de/responder.py?name=gene!Bdi!2818) | Homeobox domain containing protein, expressed | [ORTHO005259](http://aranet.mpimp-golm.mpg.de/responder.py?name=fam!fam!ORTHO005259) [HOM000298](http://aranet.mpimp-golm.mpg.de/responder.py?name=fam!fam!HOM000298) |
| [Bradi1g66720.1](http://aranet.mpimp-golm.mpg.de/responder.py?name=gene!Bdi!463) | [bradi1g66720](http://aranet.mpimp-golm.mpg.de/responder.py?name=gene!Bdi!463) | laccase precursor protein, putative, expressed | [Cu-oxidase](http://aranet.mpimp-golm.mpg.de/responder.py?name=fam!fam!Cu-oxidase) [Cu-oxidase_2](http://aranet.mpimp-golm.mpg.de/responder.py?name=fam!fam!Cu-oxidase_2) [Cu-oxidase_3](http://aranet.mpimp-golm.mpg.de/responder.py?name=fam!fam!Cu-oxidase_3) [ORTHO000023](http://aranet.mpimp-golm.mpg.de/responder.py?name=fam!fam!ORTHO000023) [HOM000096](http://aranet.mpimp-golm.mpg.de/responder.py?name=fam!fam!HOM000096) |
| [Bradi4g42400.1](http://aranet.mpimp-golm.mpg.de/responder.py?name=gene!Bdi!20451) | [bradi4g42400](http://aranet.mpimp-golm.mpg.de/responder.py?name=gene!Bdi!20451) | RCN1 Centroradialis-like1 homogous to TFL1 gene; contains Pfam profile PF01161: Phosphatidylethanolamine-binding protein, expressed | [ORTHO010160](http://aranet.mpimp-golm.mpg.de/responder.py?name=fam!fam!ORTHO010160) [HOM000381](http://aranet.mpimp-golm.mpg.de/responder.py?name=fam!fam!HOM000381) |
| [Bradi2g21300.1](http://aranet.mpimp-golm.mpg.de/responder.py?name=gene!Bdi!18584) | [bradi2g21300](http://aranet.mpimp-golm.mpg.de/responder.py?name=gene!Bdi!18584) | cytochrome P450, putative, expressed | [p450](http://aranet.mpimp-golm.mpg.de/responder.py?name=fam!fam!p450) [ORTHO001576](http://aranet.mpimp-golm.mpg.de/responder.py?name=fam!fam!ORTHO001576) [HOM000005](http://aranet.mpimp-golm.mpg.de/responder.py?name=fam!fam!HOM000005) |
| [Bradi5g25090.1](http://aranet.mpimp-golm.mpg.de/responder.py?name=gene!Bdi!3074) | [bradi5g25090](http://aranet.mpimp-golm.mpg.de/responder.py?name=gene!Bdi!3074) | IQ calmodulin-binding motif family protein, putative, expressed | [ORTHO019770](http://aranet.mpimp-golm.mpg.de/responder.py?name=fam!fam!ORTHO019770) [HOM005563](http://aranet.mpimp-golm.mpg.de/responder.py?name=fam!fam!HOM005563) |
| [Bradi2g47330.1](http://aranet.mpimp-golm.mpg.de/responder.py?name=gene!Bdi!5753) | [bradi2g47330](http://aranet.mpimp-golm.mpg.de/responder.py?name=gene!Bdi!5753) | MDR-like ABC transporter, putative, expressed | [ABC_membrane](http://aranet.mpimp-golm.mpg.de/responder.py?name=fam!fam!ABC_membrane) [ABC_tran](http://aranet.mpimp-golm.mpg.de/responder.py?name=fam!fam!ABC_tran) [ORTHO000089](http://aranet.mpimp-golm.mpg.de/responder.py?name=fam!fam!ORTHO000089) [HOM000071](http://aranet.mpimp-golm.mpg.de/responder.py?name=fam!fam!HOM000071) |
| [Bradi3g09500.1](http://aranet.mpimp-golm.mpg.de/responder.py?name=gene!Bdi!23274) | [bradi3g09500](http://aranet.mpimp-golm.mpg.de/responder.py?name=gene!Bdi!23274) | sulfotransferase domain containing protein, expressed | [Sulfotransfer_1](http://aranet.mpimp-golm.mpg.de/responder.py?name=fam!fam!Sulfotransfer_1) [ORTHO003089](http://aranet.mpimp-golm.mpg.de/responder.py?name=fam!fam!ORTHO003089) [HOM000207](http://aranet.mpimp-golm.mpg.de/responder.py?name=fam!fam!HOM000207) |
| [Bradi2g56970.1](http://aranet.mpimp-golm.mpg.de/responder.py?name=gene!Bdi!14724) | [bradi2g56970](http://aranet.mpimp-golm.mpg.de/responder.py?name=gene!Bdi!14724) | amino acid transporter, putative, expressed | [Aa_trans](http://aranet.mpimp-golm.mpg.de/responder.py?name=fam!fam!Aa_trans) [Trp_Tyr_perm](http://aranet.mpimp-golm.mpg.de/responder.py?name=fam!fam!Trp_Tyr_perm) [ORTHO000124](http://aranet.mpimp-golm.mpg.de/responder.py?name=fam!fam!ORTHO000124) [HOM000159](http://aranet.mpimp-golm.mpg.de/responder.py?name=fam!fam!HOM000159) |
| [Bradi4g29290.1](http://aranet.mpimp-golm.mpg.de/responder.py?name=gene!Bdi!7211) | [bradi4g29290](http://aranet.mpimp-golm.mpg.de/responder.py?name=gene!Bdi!7211) | serine/threonine-protein kinase, putative, expressed | [Pkinase](http://aranet.mpimp-golm.mpg.de/responder.py?name=fam!fam!Pkinase) [Pkinase_Tyr](http://aranet.mpimp-golm.mpg.de/responder.py?name=fam!fam!Pkinase_Tyr) [ORTHO004849](http://aranet.mpimp-golm.mpg.de/responder.py?name=fam!fam!ORTHO004849) [HOM000004](http://aranet.mpimp-golm.mpg.de/responder.py?name=fam!fam!HOM000004) |
| [Bradi1g07060.1](http://aranet.mpimp-golm.mpg.de/responder.py?name=gene!Bdi!22849) | [bradi1g07060](http://aranet.mpimp-golm.mpg.de/responder.py?name=gene!Bdi!22849) | expressed protein | [ORTHO018945](http://aranet.mpimp-golm.mpg.de/responder.py?name=fam!fam!ORTHO018945) [HOM010761](http://aranet.mpimp-golm.mpg.de/responder.py?name=fam!fam!HOM010761) |
| [Bradi3g36887.1](http://aranet.mpimp-golm.mpg.de/responder.py?name=gene!Bdi!3202) | [bradi3g36887](http://aranet.mpimp-golm.mpg.de/responder.py?name=gene!Bdi!3202) | cinnamoyl-CoA reductase, putative, expressed | [ORTHO002362](http://aranet.mpimp-golm.mpg.de/responder.py?name=fam!fam!ORTHO002362) [HOM000069](http://aranet.mpimp-golm.mpg.de/responder.py?name=fam!fam!HOM000069) |
| [Bradi1g21300.1](http://aranet.mpimp-golm.mpg.de/responder.py?name=gene!Bdi!4150) | [bradi1g21300](http://aranet.mpimp-golm.mpg.de/responder.py?name=gene!Bdi!4150) | dehydrogenase, putative, expressed | [ADH_N](http://aranet.mpimp-golm.mpg.de/responder.py?name=fam!fam!ADH_N) [ADH_zinc_N](http://aranet.mpimp-golm.mpg.de/responder.py?name=fam!fam!ADH_zinc_N) [ORTHO003203](http://aranet.mpimp-golm.mpg.de/responder.py?name=fam!fam!ORTHO003203) [HOM000247](http://aranet.mpimp-golm.mpg.de/responder.py?name=fam!fam!HOM000247) |
| [Bradi3g49260.1](http://aranet.mpimp-golm.mpg.de/responder.py?name=gene!Bdi!8486) | [bradi3g49260](http://aranet.mpimp-golm.mpg.de/responder.py?name=gene!Bdi!8486) | phenylalanine ammonia-lyase, putative, expressed | [PAL](http://aranet.mpimp-golm.mpg.de/responder.py?name=fam!fam!PAL) [ORTHO000087](http://aranet.mpimp-golm.mpg.de/responder.py?name=fam!fam!ORTHO000087) [HOM000613](http://aranet.mpimp-golm.mpg.de/responder.py?name=fam!fam!HOM000613) |
| [Bradi1g59160.1](http://aranet.mpimp-golm.mpg.de/responder.py?name=gene!Bdi!2459) | [bradi1g59160](http://aranet.mpimp-golm.mpg.de/responder.py?name=gene!Bdi!2459) | flavin-containing monooxygenase family protein, putative, expressed | [DAO](http://aranet.mpimp-golm.mpg.de/responder.py?name=fam!fam!DAO) [FMO-like](http://aranet.mpimp-golm.mpg.de/responder.py?name=fam!fam!FMO-like) [Pyr_redox_2](http://aranet.mpimp-golm.mpg.de/responder.py?name=fam!fam!Pyr_redox_2) [HOM000653](http://aranet.mpimp-golm.mpg.de/responder.py?name=fam!fam!HOM000653) |
| [Bradi4g21790.1](http://aranet.mpimp-golm.mpg.de/responder.py?name=gene!Bdi!14031) | [bradi4g21790](http://aranet.mpimp-golm.mpg.de/responder.py?name=gene!Bdi!14031) | peptide transporter PTR2, putative, expressed | [PTR2](http://aranet.mpimp-golm.mpg.de/responder.py?name=fam!fam!PTR2) [ORTHO002126](http://aranet.mpimp-golm.mpg.de/responder.py?name=fam!fam!ORTHO002126) [HOM000031](http://aranet.mpimp-golm.mpg.de/responder.py?name=fam!fam!HOM000031) |
| [Bradi2g23300.1](http://aranet.mpimp-golm.mpg.de/responder.py?name=gene!Bdi!1086) | [bradi2g23300](http://aranet.mpimp-golm.mpg.de/responder.py?name=gene!Bdi!1086) | expressed protein | [ORTHO008709](http://aranet.mpimp-golm.mpg.de/responder.py?name=fam!fam!ORTHO008709) [HOM005338](http://aranet.mpimp-golm.mpg.de/responder.py?name=fam!fam!HOM005338) |
| [Bradi1g72350.1](http://aranet.mpimp-golm.mpg.de/responder.py?name=gene!Bdi!9140) | [bradi1g72350](http://aranet.mpimp-golm.mpg.de/responder.py?name=gene!Bdi!9140) | glycosyl transferase, putative, expressed | [Glyco_transf_8](http://aranet.mpimp-golm.mpg.de/responder.py?name=fam!fam!Glyco_transf_8) [ORTHO005972](http://aranet.mpimp-golm.mpg.de/responder.py?name=fam!fam!ORTHO005972) [HOM001012](http://aranet.mpimp-golm.mpg.de/responder.py?name=fam!fam!HOM001012) |
| [Bradi3g04080.1](http://aranet.mpimp-golm.mpg.de/responder.py?name=gene!Bdi!17804) | [bradi3g04080](http://aranet.mpimp-golm.mpg.de/responder.py?name=gene!Bdi!17804) | endoglucanase, putative, expressed | [Glyco_hydro_9](http://aranet.mpimp-golm.mpg.de/responder.py?name=fam!fam!Glyco_hydro_9) [ORTHO000312](http://aranet.mpimp-golm.mpg.de/responder.py?name=fam!fam!ORTHO000312) [HOM000137](http://aranet.mpimp-golm.mpg.de/responder.py?name=fam!fam!HOM000137) |
| [Bradi3g47950.1](http://aranet.mpimp-golm.mpg.de/responder.py?name=gene!Bdi!8300) | [bradi3g47950](http://aranet.mpimp-golm.mpg.de/responder.py?name=gene!Bdi!8300) | expressed protein | [Ank](http://aranet.mpimp-golm.mpg.de/responder.py?name=fam!fam!Ank) [ORTHO001122](http://aranet.mpimp-golm.mpg.de/responder.py?name=fam!fam!ORTHO001122) [HOM002074](http://aranet.mpimp-golm.mpg.de/responder.py?name=fam!fam!HOM002074) |
| [Bradi2g49040.1](http://aranet.mpimp-golm.mpg.de/responder.py?name=gene!Bdi!16017) | [bradi2g49040](http://aranet.mpimp-golm.mpg.de/responder.py?name=gene!Bdi!16017) | respiratory burst oxidase, putative, expressed | [FAD_binding_8](http://aranet.mpimp-golm.mpg.de/responder.py?name=fam!fam!FAD_binding_8) [Ferric_reduct](http://aranet.mpimp-golm.mpg.de/responder.py?name=fam!fam!Ferric_reduct) [NADPH_Ox](http://aranet.mpimp-golm.mpg.de/responder.py?name=fam!fam!NADPH_Ox) [NAD_binding_6](http://aranet.mpimp-golm.mpg.de/responder.py?name=fam!fam!NAD_binding_6) [ORTHO000035](http://aranet.mpimp-golm.mpg.de/responder.py?name=fam!fam!ORTHO000035) [HOM000327](http://aranet.mpimp-golm.mpg.de/responder.py?name=fam!fam!HOM000327) |
| [Bradi5g15490.1](http://aranet.mpimp-golm.mpg.de/responder.py?name=gene!Bdi!11346) | [bradi5g15490](http://aranet.mpimp-golm.mpg.de/responder.py?name=gene!Bdi!11346) | ARPC2B, putative, expressed | [ORTHO008608](http://aranet.mpimp-golm.mpg.de/responder.py?name=fam!fam!ORTHO008608) [HOM002240](http://aranet.mpimp-golm.mpg.de/responder.py?name=fam!fam!HOM002240) |
| [Bradi5g20130.1](http://aranet.mpimp-golm.mpg.de/responder.py?name=gene!Bdi!4141) | [bradi5g20130](http://aranet.mpimp-golm.mpg.de/responder.py?name=gene!Bdi!4141) | MYB family transcription factor, putative, expressed | [ORTHO004899](http://aranet.mpimp-golm.mpg.de/responder.py?name=fam!fam!ORTHO004899) [HOM000007](http://aranet.mpimp-golm.mpg.de/responder.py?name=fam!fam!HOM000007) |
| [Bradi3g37230.1](http://aranet.mpimp-golm.mpg.de/responder.py?name=gene!Bdi!12449) | [bradi3g37230](http://aranet.mpimp-golm.mpg.de/responder.py?name=gene!Bdi!12449) | GDU1, putative, expressed | [ORTHO010986](http://aranet.mpimp-golm.mpg.de/responder.py?name=fam!fam!ORTHO010986) [HOM000875](http://aranet.mpimp-golm.mpg.de/responder.py?name=fam!fam!HOM000875) |
| [Bradi4g34300.1](http://aranet.mpimp-golm.mpg.de/responder.py?name=gene!Bdi!11151) | [bradi4g34300](http://aranet.mpimp-golm.mpg.de/responder.py?name=gene!Bdi!11151) | membrane protein, putative, expressed | [Cytochrom_B561](http://aranet.mpimp-golm.mpg.de/responder.py?name=fam!fam!Cytochrom_B561) [Cytochrom_C_asm](http://aranet.mpimp-golm.mpg.de/responder.py?name=fam!fam!Cytochrom_C_asm) [DUF568](http://aranet.mpimp-golm.mpg.de/responder.py?name=fam!fam!DUF568) [ORTHO003253](http://aranet.mpimp-golm.mpg.de/responder.py?name=fam!fam!ORTHO003253) [HOM000285](http://aranet.mpimp-golm.mpg.de/responder.py?name=fam!fam!HOM000285) |
| [Bradi2g52060.2](http://aranet.mpimp-golm.mpg.de/responder.py?name=gene!Bdi!21151) | [bradi2g52060](http://aranet.mpimp-golm.mpg.de/responder.py?name=gene!Bdi!21151) | membrane protein, putative, expressed | [TauE](http://aranet.mpimp-golm.mpg.de/responder.py?name=fam!fam!TauE) [ORTHO004911](http://aranet.mpimp-golm.mpg.de/responder.py?name=fam!fam!ORTHO004911) [HOM000500](http://aranet.mpimp-golm.mpg.de/responder.py?name=fam!fam!HOM000500) |
| [Bradi1g69870.1](http://aranet.mpimp-golm.mpg.de/responder.py?name=gene!Bdi!12232) | [bradi1g69870](http://aranet.mpimp-golm.mpg.de/responder.py?name=gene!Bdi!12232) | ATMAP70 protein, putative, expressed | [Myosin_HC-like](http://aranet.mpimp-golm.mpg.de/responder.py?name=fam!fam!Myosin_HC-like) [ORTHO021861](http://aranet.mpimp-golm.mpg.de/responder.py?name=fam!fam!ORTHO021861) [HOM001162](http://aranet.mpimp-golm.mpg.de/responder.py?name=fam!fam!HOM001162) |
| [Bradi4g30437.1](http://aranet.mpimp-golm.mpg.de/responder.py?name=gene!Bdi!13127) | [bradi4g30437](http://aranet.mpimp-golm.mpg.de/responder.py?name=gene!Bdi!13127) | methyladenine glycosylase, putative, expressed | [ORTHO023180](http://aranet.mpimp-golm.mpg.de/responder.py?name=fam!fam!ORTHO023180) [HOM000675](http://aranet.mpimp-golm.mpg.de/responder.py?name=fam!fam!HOM000675) |
| [Bradi1g22980.1](http://aranet.mpimp-golm.mpg.de/responder.py?name=gene!Bdi!3972) | [bradi1g22980](http://aranet.mpimp-golm.mpg.de/responder.py?name=gene!Bdi!3972) | formin, putative, expressed | [FH2](http://aranet.mpimp-golm.mpg.de/responder.py?name=fam!fam!FH2) [ORTHO021498](http://aranet.mpimp-golm.mpg.de/responder.py?name=fam!fam!ORTHO021498) [HOM000319](http://aranet.mpimp-golm.mpg.de/responder.py?name=fam!fam!HOM000319) |
| [Bradi3g56787.1](http://aranet.mpimp-golm.mpg.de/responder.py?name=gene!Bdi!10643) | [bradi3g56787](http://aranet.mpimp-golm.mpg.de/responder.py?name=gene!Bdi!10643) | polygalacturonase, putative, expressed | [ORTHO001780](http://aranet.mpimp-golm.mpg.de/responder.py?name=fam!fam!ORTHO001780) [HOM000297](http://aranet.mpimp-golm.mpg.de/responder.py?name=fam!fam!HOM000297) |
| [Bradi1g51960.1](http://aranet.mpimp-golm.mpg.de/responder.py?name=gene!Bdi!4690) | [bradi1g51960](http://aranet.mpimp-golm.mpg.de/responder.py?name=gene!Bdi!4690) | RGH2B, putative, expressed | [Myb_DNA-binding](http://aranet.mpimp-golm.mpg.de/responder.py?name=fam!fam!Myb_DNA-binding) [HOM000007](http://aranet.mpimp-golm.mpg.de/responder.py?name=fam!fam!HOM000007) |
| [Bradi2g55730.1](http://aranet.mpimp-golm.mpg.de/responder.py?name=gene!Bdi!22708) | [bradi2g55730](http://aranet.mpimp-golm.mpg.de/responder.py?name=gene!Bdi!22708) | cytochrome P450, putative, expressed | [AATase](http://aranet.mpimp-golm.mpg.de/responder.py?name=fam!fam!AATase) [Condensation](http://aranet.mpimp-golm.mpg.de/responder.py?name=fam!fam!Condensation) [Transferase](http://aranet.mpimp-golm.mpg.de/responder.py?name=fam!fam!Transferase) [ORTHO007979](http://aranet.mpimp-golm.mpg.de/responder.py?name=fam!fam!ORTHO007979) [HOM003488](http://aranet.mpimp-golm.mpg.de/responder.py?name=fam!fam!HOM003488) |
| [Bradi3g05750.1](http://aranet.mpimp-golm.mpg.de/responder.py?name=gene!Bdi!8338) | [bradi3g05750](http://aranet.mpimp-golm.mpg.de/responder.py?name=gene!Bdi!8338) | AMP-binding domain containing protein, expressed | [AMP-binding](http://aranet.mpimp-golm.mpg.de/responder.py?name=fam!fam!AMP-binding) [ORTHO000228](http://aranet.mpimp-golm.mpg.de/responder.py?name=fam!fam!ORTHO000228) [HOM000212](http://aranet.mpimp-golm.mpg.de/responder.py?name=fam!fam!HOM000212) |
| [Bradi2g54240.1](http://aranet.mpimp-golm.mpg.de/responder.py?name=gene!Bdi!9318) | [bradi2g54240](http://aranet.mpimp-golm.mpg.de/responder.py?name=gene!Bdi!9318) | respiratory burst oxidase, putative, expressed | [FAD_binding_8](http://aranet.mpimp-golm.mpg.de/responder.py?name=fam!fam!FAD_binding_8) [Ferric_reduct](http://aranet.mpimp-golm.mpg.de/responder.py?name=fam!fam!Ferric_reduct) [NADPH_Ox](http://aranet.mpimp-golm.mpg.de/responder.py?name=fam!fam!NADPH_Ox) [NAD_binding_6](http://aranet.mpimp-golm.mpg.de/responder.py?name=fam!fam!NAD_binding_6) [ORTHO000035](http://aranet.mpimp-golm.mpg.de/responder.py?name=fam!fam!ORTHO000035) [HOM000327](http://aranet.mpimp-golm.mpg.de/responder.py?name=fam!fam!HOM000327) |
| [Bradi2g55720.1](http://aranet.mpimp-golm.mpg.de/responder.py?name=gene!Bdi!13518) | [bradi2g55720](http://aranet.mpimp-golm.mpg.de/responder.py?name=gene!Bdi!13518) | hemerythrin family protein, expressed | [Hemerythrin](http://aranet.mpimp-golm.mpg.de/responder.py?name=fam!fam!Hemerythrin) [ORTHO007983](http://aranet.mpimp-golm.mpg.de/responder.py?name=fam!fam!ORTHO007983) [HOM003674](http://aranet.mpimp-golm.mpg.de/responder.py?name=fam!fam!HOM003674) |
| [Bradi1g32850.1](http://aranet.mpimp-golm.mpg.de/responder.py?name=gene!Bdi!18682) | [bradi1g32850](http://aranet.mpimp-golm.mpg.de/responder.py?name=gene!Bdi!18682) | RIC10, putative, expressed | [PBD](http://aranet.mpimp-golm.mpg.de/responder.py?name=fam!fam!PBD) [HOM001247](http://aranet.mpimp-golm.mpg.de/responder.py?name=fam!fam!HOM001247) |
| [Bradi1g14050.1](http://aranet.mpimp-golm.mpg.de/responder.py?name=gene!Bdi!12471) | [bradi1g14050](http://aranet.mpimp-golm.mpg.de/responder.py?name=gene!Bdi!12471) | uncharacterized protein At4g06744 precursor, putative, expressed | [LRRNT_2](http://aranet.mpimp-golm.mpg.de/responder.py?name=fam!fam!LRRNT_2) [ORTHO018969](http://aranet.mpimp-golm.mpg.de/responder.py?name=fam!fam!ORTHO018969) [HOM000308](http://aranet.mpimp-golm.mpg.de/responder.py?name=fam!fam!HOM000308) |
| [Bradi1g17680.1](http://aranet.mpimp-golm.mpg.de/responder.py?name=gene!Bdi!15815) | [bradi1g17680](http://aranet.mpimp-golm.mpg.de/responder.py?name=gene!Bdi!15815) | B3 DNA binding domain containing protein, putative, expressed | [B3](http://aranet.mpimp-golm.mpg.de/responder.py?name=fam!fam!B3) [zf-CW](http://aranet.mpimp-golm.mpg.de/responder.py?name=fam!fam!zf-CW) [ORTHO000950](http://aranet.mpimp-golm.mpg.de/responder.py?name=fam!fam!ORTHO000950) [HOM001382](http://aranet.mpimp-golm.mpg.de/responder.py?name=fam!fam!HOM001382) |
| [Bradi2g58490.1](http://aranet.mpimp-golm.mpg.de/responder.py?name=gene!Bdi!2518) | [bradi2g58490](http://aranet.mpimp-golm.mpg.de/responder.py?name=gene!Bdi!2518) | rho GDP-dissociation inhibitor 1, putative, expressed | [Rho_GDI](http://aranet.mpimp-golm.mpg.de/responder.py?name=fam!fam!Rho_GDI) [ORTHO019378](http://aranet.mpimp-golm.mpg.de/responder.py?name=fam!fam!ORTHO019378) [HOM001151](http://aranet.mpimp-golm.mpg.de/responder.py?name=fam!fam!HOM001151) |
| [Bradi5g15527.1](http://aranet.mpimp-golm.mpg.de/responder.py?name=gene!Bdi!15491) | [bradi5g15527](http://aranet.mpimp-golm.mpg.de/responder.py?name=gene!Bdi!15491) | Os4bglu14 - monolignol beta-glucoside homologue without catalytic acid/base, expressed | [ORTHO023384](http://aranet.mpimp-golm.mpg.de/responder.py?name=fam!fam!ORTHO023384) [HOM000064](http://aranet.mpimp-golm.mpg.de/responder.py?name=fam!fam!HOM000064) |
| [Bradi1g28366.1](http://aranet.mpimp-golm.mpg.de/responder.py?name=gene!Bdi!6193) | [bradi1g28366](http://aranet.mpimp-golm.mpg.de/responder.py?name=gene!Bdi!6193) | alpha-1,2-fucosidase, putative, expressed | [ORTHO002054](http://aranet.mpimp-golm.mpg.de/responder.py?name=fam!fam!ORTHO002054) [HOM003010](http://aranet.mpimp-golm.mpg.de/responder.py?name=fam!fam!HOM003010) |
| [Bradi3g49250.2](http://aranet.mpimp-golm.mpg.de/responder.py?name=gene!Bdi!23487) | [bradi3g49250](http://aranet.mpimp-golm.mpg.de/responder.py?name=gene!Bdi!23487) | phenylalanine ammonia-lyase, putative, expressed | [PAL](http://aranet.mpimp-golm.mpg.de/responder.py?name=fam!fam!PAL) [ORTHO000087](http://aranet.mpimp-golm.mpg.de/responder.py?name=fam!fam!ORTHO000087) [HOM000613](http://aranet.mpimp-golm.mpg.de/responder.py?name=fam!fam!HOM000613) |
| [Bradi2g34790.1](http://aranet.mpimp-golm.mpg.de/responder.py?name=gene!Bdi!15702) | [bradi2g34790](http://aranet.mpimp-golm.mpg.de/responder.py?name=gene!Bdi!15702) | GDSL-like lipase/acylhydrolase, putative, expressed | [Lipase_GDSL](http://aranet.mpimp-golm.mpg.de/responder.py?name=fam!fam!Lipase_GDSL) [HOM000086](http://aranet.mpimp-golm.mpg.de/responder.py?name=fam!fam!HOM000086) |
| [Bradi1g59880.2](http://aranet.mpimp-golm.mpg.de/responder.py?name=gene!Bdi!23320) | [bradi1g59880](http://aranet.mpimp-golm.mpg.de/responder.py?name=gene!Bdi!23320) | COBRA-like protein precursor, putative, expressed | [COBRA](http://aranet.mpimp-golm.mpg.de/responder.py?name=fam!fam!COBRA) [ORTHO001111](http://aranet.mpimp-golm.mpg.de/responder.py?name=fam!fam!ORTHO001111) [HOM000604](http://aranet.mpimp-golm.mpg.de/responder.py?name=fam!fam!HOM000604) |
| [Bradi5g15850.1](http://aranet.mpimp-golm.mpg.de/responder.py?name=gene!Bdi!19937) | [bradi5g15850](http://aranet.mpimp-golm.mpg.de/responder.py?name=gene!Bdi!19937) | transporter-related, putative, expressed | [ORTHO008332](http://aranet.mpimp-golm.mpg.de/responder.py?name=fam!fam!ORTHO008332) [HOM000490](http://aranet.mpimp-golm.mpg.de/responder.py?name=fam!fam!HOM000490) |
| [Bradi2g47600.1](http://aranet.mpimp-golm.mpg.de/responder.py?name=gene!Bdi!15143) | [bradi2g47600](http://aranet.mpimp-golm.mpg.de/responder.py?name=gene!Bdi!15143) | zinc finger, C3HC4 type domain containing protein, expressed | [zf-C3HC4](http://aranet.mpimp-golm.mpg.de/responder.py?name=fam!fam!zf-C3HC4) [ORTHO009670](http://aranet.mpimp-golm.mpg.de/responder.py?name=fam!fam!ORTHO009670) [HOM000242](http://aranet.mpimp-golm.mpg.de/responder.py?name=fam!fam!HOM000242) |
| [Bradi1g10470.1](http://aranet.mpimp-golm.mpg.de/responder.py?name=gene!Bdi!3920) | [bradi1g10470](http://aranet.mpimp-golm.mpg.de/responder.py?name=gene!Bdi!3920) | MYB family transcription factor, putative, expressed | [Myb_DNA-binding](http://aranet.mpimp-golm.mpg.de/responder.py?name=fam!fam!Myb_DNA-binding) [ORTHO006933](http://aranet.mpimp-golm.mpg.de/responder.py?name=fam!fam!ORTHO006933) [HOM000007](http://aranet.mpimp-golm.mpg.de/responder.py?name=fam!fam!HOM000007) |
| [Bradi2g08790.1](http://aranet.mpimp-golm.mpg.de/responder.py?name=gene!Bdi!8323) | [bradi2g08790](http://aranet.mpimp-golm.mpg.de/responder.py?name=gene!Bdi!8323) | Cupin domain containing protein, expressed | [Cupin_1](http://aranet.mpimp-golm.mpg.de/responder.py?name=fam!fam!Cupin_1) [ORTHO000182](http://aranet.mpimp-golm.mpg.de/responder.py?name=fam!fam!ORTHO000182) [HOM000084](http://aranet.mpimp-golm.mpg.de/responder.py?name=fam!fam!HOM000084) |
| [Bradi2g46410.1](http://aranet.mpimp-golm.mpg.de/responder.py?name=gene!Bdi!5130) | [bradi2g46410](http://aranet.mpimp-golm.mpg.de/responder.py?name=gene!Bdi!5130) | glycosyltransferase family 43 protein, putative, expressed | [Glyco_transf_43](http://aranet.mpimp-golm.mpg.de/responder.py?name=fam!fam!Glyco_transf_43) [ORTHO012201](http://aranet.mpimp-golm.mpg.de/responder.py?name=fam!fam!ORTHO012201) [HOM001149](http://aranet.mpimp-golm.mpg.de/responder.py?name=fam!fam!HOM001149) |
| [Bradi4g44860.1](http://aranet.mpimp-golm.mpg.de/responder.py?name=gene!Bdi!11487) | [bradi4g44860](http://aranet.mpimp-golm.mpg.de/responder.py?name=gene!Bdi!11487) | PMR5, putative, expressed | [ORTHO013293](http://aranet.mpimp-golm.mpg.de/responder.py?name=fam!fam!ORTHO013293) [HOM000050](http://aranet.mpimp-golm.mpg.de/responder.py?name=fam!fam!HOM000050) |
| [Bradi1g68710.2](http://aranet.mpimp-golm.mpg.de/responder.py?name=gene!Bdi!16624) | [bradi1g68710](http://aranet.mpimp-golm.mpg.de/responder.py?name=gene!Bdi!16624) | microtubule associated protein, putative, expressed | [MAP65_ASE1](http://aranet.mpimp-golm.mpg.de/responder.py?name=fam!fam!MAP65_ASE1) [ORTHO006227](http://aranet.mpimp-golm.mpg.de/responder.py?name=fam!fam!ORTHO006227) [HOM000492](http://aranet.mpimp-golm.mpg.de/responder.py?name=fam!fam!HOM000492) |
| [Bradi3g37530.1](http://aranet.mpimp-golm.mpg.de/responder.py?name=gene!Bdi!4201) | [bradi3g37530](http://aranet.mpimp-golm.mpg.de/responder.py?name=gene!Bdi!4201) | ferric reductase, putative, expressed | [FAD_binding_8](http://aranet.mpimp-golm.mpg.de/responder.py?name=fam!fam!FAD_binding_8) [Ferric_reduct](http://aranet.mpimp-golm.mpg.de/responder.py?name=fam!fam!Ferric_reduct) [NADPH_Ox](http://aranet.mpimp-golm.mpg.de/responder.py?name=fam!fam!NADPH_Ox) [NAD_binding_6](http://aranet.mpimp-golm.mpg.de/responder.py?name=fam!fam!NAD_binding_6) [ORTHO000035](http://aranet.mpimp-golm.mpg.de/responder.py?name=fam!fam!ORTHO000035) [HOM000327](http://aranet.mpimp-golm.mpg.de/responder.py?name=fam!fam!HOM000327) |
| [Bradi1g04750.1](http://aranet.mpimp-golm.mpg.de/responder.py?name=gene!Bdi!16356) | [bradi1g04750](http://aranet.mpimp-golm.mpg.de/responder.py?name=gene!Bdi!16356) | expressed protein | [ORTHO021331](http://aranet.mpimp-golm.mpg.de/responder.py?name=fam!fam!ORTHO021331) [HOM000834](http://aranet.mpimp-golm.mpg.de/responder.py?name=fam!fam!HOM000834) |
| [Bradi5g01737.1](http://aranet.mpimp-golm.mpg.de/responder.py?name=gene!Bdi!17692) | [bradi5g01737](http://aranet.mpimp-golm.mpg.de/responder.py?name=gene!Bdi!17692) | peptide transporter PTR2, putative, expressed | [ORTHO014217](http://aranet.mpimp-golm.mpg.de/responder.py?name=fam!fam!ORTHO014217) [HOM000031](http://aranet.mpimp-golm.mpg.de/responder.py?name=fam!fam!HOM000031) |
| [Bradi3g05860.1](http://aranet.mpimp-golm.mpg.de/responder.py?name=gene!Bdi!9666) | [bradi3g05860](http://aranet.mpimp-golm.mpg.de/responder.py?name=gene!Bdi!9666) | flavin-containing monooxygenase family protein, putative, expressed | [FMO-like](http://aranet.mpimp-golm.mpg.de/responder.py?name=fam!fam!FMO-like) [Pyr_redox_2](http://aranet.mpimp-golm.mpg.de/responder.py?name=fam!fam!Pyr_redox_2) [ORTHO047108](http://aranet.mpimp-golm.mpg.de/responder.py?name=fam!fam!ORTHO047108) [HOM000653](http://aranet.mpimp-golm.mpg.de/responder.py?name=fam!fam!HOM000653) |
| [Bradi2g59410.1](http://aranet.mpimp-golm.mpg.de/responder.py?name=gene!Bdi!3856) | [bradi2g59410](http://aranet.mpimp-golm.mpg.de/responder.py?name=gene!Bdi!3856) | exostosin family domain containing protein, expressed | [Exostosin](http://aranet.mpimp-golm.mpg.de/responder.py?name=fam!fam!Exostosin) [ORTHO000379](http://aranet.mpimp-golm.mpg.de/responder.py?name=fam!fam!ORTHO000379) [HOM001006](http://aranet.mpimp-golm.mpg.de/responder.py?name=fam!fam!HOM001006) |
| [Bradi1g65430.1](http://aranet.mpimp-golm.mpg.de/responder.py?name=gene!Bdi!14412) | [bradi1g65430](http://aranet.mpimp-golm.mpg.de/responder.py?name=gene!Bdi!14412) | chloroplast unusual positioning protein, putative, expressed | [DUF729](http://aranet.mpimp-golm.mpg.de/responder.py?name=fam!fam!DUF729) [ORTHO009842](http://aranet.mpimp-golm.mpg.de/responder.py?name=fam!fam!ORTHO009842) [HOM000174](http://aranet.mpimp-golm.mpg.de/responder.py?name=fam!fam!HOM000174) |
| [Bradi4g22250.1](http://aranet.mpimp-golm.mpg.de/responder.py?name=gene!Bdi!16482) | [bradi4g22250](http://aranet.mpimp-golm.mpg.de/responder.py?name=gene!Bdi!16482) | dirigent, putative, expressed | [Dirigent](http://aranet.mpimp-golm.mpg.de/responder.py?name=fam!fam!Dirigent) [ORTHO016006](http://aranet.mpimp-golm.mpg.de/responder.py?name=fam!fam!ORTHO016006) [HOM000170](http://aranet.mpimp-golm.mpg.de/responder.py?name=fam!fam!HOM000170) |
| [Bradi2g49912.1](http://aranet.mpimp-golm.mpg.de/responder.py?name=gene!Bdi!18830) | [bradi2g49912](http://aranet.mpimp-golm.mpg.de/responder.py?name=gene!Bdi!18830) | CESA4 - cellulose synthase, expressed | [ORTHO000003](http://aranet.mpimp-golm.mpg.de/responder.py?name=fam!fam!ORTHO000003) [HOM000082](http://aranet.mpimp-golm.mpg.de/responder.py?name=fam!fam!HOM000082) |
| [Bradi2g12150.2](http://aranet.mpimp-golm.mpg.de/responder.py?name=gene!Bdi!8965) | [bradi2g12150](http://aranet.mpimp-golm.mpg.de/responder.py?name=gene!Bdi!8965) | S-adenosylmethionine synthetase, putative, expressed | [S-AdoMet_synt_C](http://aranet.mpimp-golm.mpg.de/responder.py?name=fam!fam!S-AdoMet_synt_C) [S-AdoMet_synt_M](http://aranet.mpimp-golm.mpg.de/responder.py?name=fam!fam!S-AdoMet_synt_M) [S-AdoMet_synt_N](http://aranet.mpimp-golm.mpg.de/responder.py?name=fam!fam!S-AdoMet_synt_N) [ORTHO000163](http://aranet.mpimp-golm.mpg.de/responder.py?name=fam!fam!ORTHO000163) [HOM000932](http://aranet.mpimp-golm.mpg.de/responder.py?name=fam!fam!HOM000932) |
| [Bradi1g67870.1](http://aranet.mpimp-golm.mpg.de/responder.py?name=gene!Bdi!20520) | [bradi1g67870](http://aranet.mpimp-golm.mpg.de/responder.py?name=gene!Bdi!20520) | expressed protein | [ORTHO008245](http://aranet.mpimp-golm.mpg.de/responder.py?name=fam!fam!ORTHO008245) [HOM004952](http://aranet.mpimp-golm.mpg.de/responder.py?name=fam!fam!HOM004952) |
| [Bradi2g45090.1](http://aranet.mpimp-golm.mpg.de/responder.py?name=gene!Bdi!11725) | [bradi2g45090](http://aranet.mpimp-golm.mpg.de/responder.py?name=gene!Bdi!11725) | zinc finger C-x8-C-x5-C-x3-H type family protein, expressed | [zf-CCCH](http://aranet.mpimp-golm.mpg.de/responder.py?name=fam!fam!zf-CCCH) [ORTHO002667](http://aranet.mpimp-golm.mpg.de/responder.py?name=fam!fam!ORTHO002667) [HOM002080](http://aranet.mpimp-golm.mpg.de/responder.py?name=fam!fam!HOM002080) |
